# *BCL11A* is a triple-negative breast cancer gene with critical functions in stem and progenitor cells

**DOI:** 10.1038/ncomms6987

**Published:** 2015-01-09

**Authors:** Walid T. Khaled, Song Choon Lee, John Stingl, Xiongfeng Chen, H. Raza Ali, Oscar M. Rueda, Fazal Hadi, Juexuan Wang, Yong Yu, Suet-Feung Chin, Mike Stratton, Andy Futreal, Nancy A. Jenkins, Sam Aparicio, Neal G. Copeland, Christine J. Watson, Carlos Caldas, Pentao Liu

**Affiliations:** 1 Wellcome Trust Sanger Institute, Hinxton, Cambridge CB10 1HH, UK; 2 Department of Pharmacology, University of Cambridge, Cambridge CB2 1PD, UK; 3 Cancer Research UK Cambridge Institute, and Department of Oncology, University of Cambridge, Li Ka Shing Centre, Robinson Way, Cambridge CB2 0RE, UK; 4 SAIC-Frederic, National Cancer Institute-Frederick, Frederick, Maryland 21701, USA; 5 Cambridge Experimental Cancer Medicine Centre, Cambridge CB2 0RE, UK; 6 The Methodist Hospital Research Institute, 6670 Bertner Street, Houston, Texas 77030, USA; 7 Molecular Oncology Department, BC Cancer Agency Research Centre, 675 West 10th Avenue, Vancouver, British Columbia V5Z 1L3, Canada; 8 Department of Pathology, University of Cambridge, Cambridge CB2 1QP, UK; 9 Addenbrooke’s Hospital, Cambridge University Hospital NHS Foundation Trust and NIHR Cambridge Biomedical Research Centre, Cambridge CB2 2QQ, UK; 10 These authors contributed equally to this work

## Abstract

Triple-negative breast cancer (TNBC) has poor prognostic outcome compared with other types of breast cancer. The molecular and cellular mechanisms underlying TNBC pathology are not fully understood. Here, we report that the transcription factor *BCL11A* is overexpressed in TNBC including basal-like breast cancer (BLBC) and that its genomic locus is amplified in up to 38% of BLBC tumours. Exogenous *BCL11A* overexpression promotes tumour formation, whereas its knockdown in TNBC cell lines suppresses their tumourigenic potential in xenograft models. In the DMBA-induced tumour model, *Bcl11a* deletion substantially decreases tumour formation, even in p53-null cells and inactivation of *Bcl11a* in established tumours causes their regression. At the cellular level, *Bcl11a* deletion causes a reduction in the number of mammary epithelial stem and progenitor cells. Thus, BCL11A has an important role in TNBC and normal mammary epithelial cells. This study highlights the importance of further investigation of *BCL11A* in TNBC-targeted therapies.

## Abstract (web summary)

Triple-negative breast cancers (TNBCs) tend to have poor prognosis; however, the mechanisms underlying TNBC pathology are not well understood. Here the authors utilize epidemiologic data and animal models to demonstrate an important role for BCL11A in the genesis and propagation of TNBCs.

## Graphical abstract

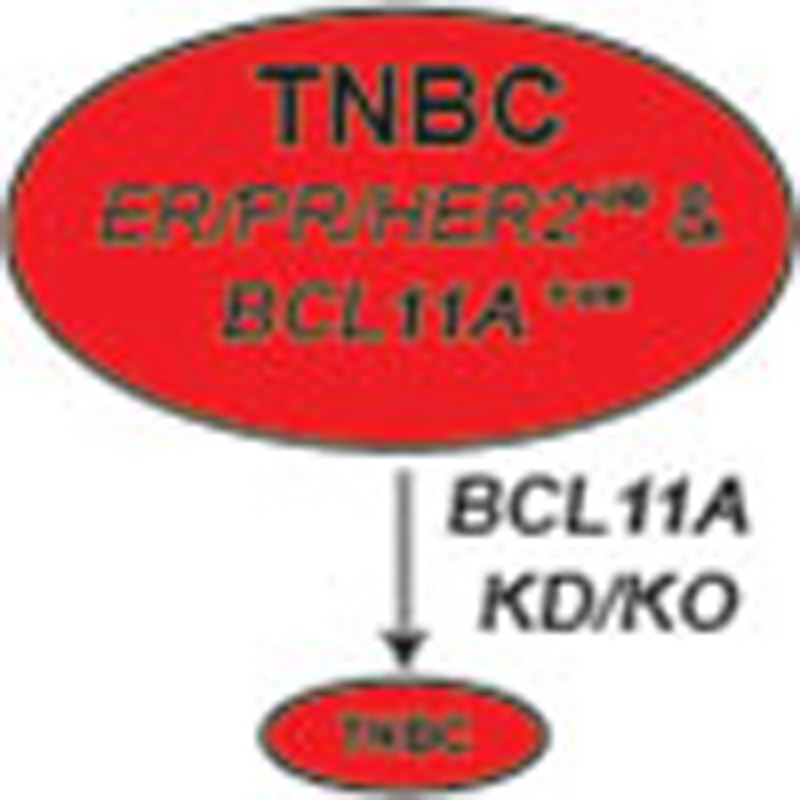

One of the major challenges in treating breast cancer is the heterogeneous nature of the disease^1^. TNBC accounts for around 15% of all breast cancer cases and in the absence of effective targeted therapies, TNBC patients tend to have a poor prognosis^2^,^3^,^4^. At the molecular level, several distinct subtypes of breast cancer have been identified based on the gene expression profiling^3^,^5^,^6^. The most commonly used classification describes six subtypes: luminal A, luminal B, Her2, claudin low, basal-like breast cancer (BLBC) and normal^3^,^6^. More recently, analysis of large numbers of tumour samples as part of the METABRIC study identified 10 pathologically distinct subtypes known as integrative cluster (IC) 1–10 (ref. 5). The majority of TNBC cases (80%) have a BLBC^7^ or IC10 (ref. 5) gene expression signatures. In addition, cancer sequencing studies have identified mutations of *p53, PTEN* and *BRCA1* in TNBC^2^,^4^,^8^,^9^. However, driver oncogenic genomic aberrations in TNBC have not been comprehensively identified.

The developmental hierarchies of the mammary epithelium and hematopoietic lineages share many similarities^10^ in that stem cells progressively give rise to lineage-restricted progenitors, which ultimately differentiate and generate all functional cells. A number of key hematopoiesis transcription factors have important roles in mouse mammary gland development and are human breast cancer genes^11^,^12^,^13^,^14^,^15^. For example, the key regulator of T-helper-2 cell development, GATA3, is critical in luminal mammary cell development^12^,^13^ and is a luminal breast cancer marker gene^16^. In this study we interrogated cancer genomics data focusing on a subset of important hematopoiesis factors and identified *BCL11A* as a novel TNBC oncogene.

## Results

### BCL11A is highly expressed in triple-negative breast cancer

In an attempt to identify potential TNBC oncogenes, we selected a list of genes known to have important roles in hematopoiesis and investigated their expression across the major molecular subtypes of breast cancer^3^. We first re-analysed a publically available microarray data set^6^ and found that out of the examined genes, *BCL11A* was differentially and highly expressed in BLBC (Supplementary Fig. 1a). This is in sharp contrast to *GATA3*, which is highly expressed only in luminal subtypes (Supplementary Fig. 1a) and is a known prognostic marker for these tumours 16.

**Figure 1:**
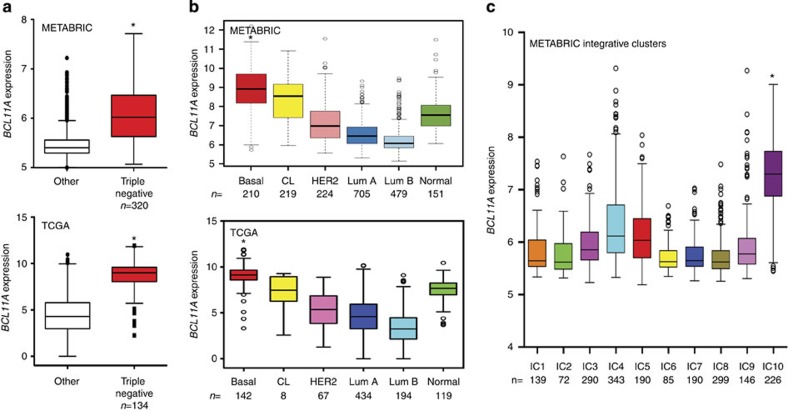
*BCL11A* is highly expressed in TNBC. (**a**) Significant correlation between *BCL11A* expression and the TNBC type of breast cancer in both METABRIC (*n*=2,000) and TCGA (*n*=1,100) data sets—* indicates *t*-test *P* value<0.005. (**b**) *BCL11A* expression across the six molecular subtypes of breast cancer (‘Normal’ refers to the PAM50 subtype) in both METABRIC and TCGA data sets—* indicates *t*-test *P* value<0.005. (**c**) The METABRIC samples distributed according to the ICs 1–10, showing the correlation between *BCL11A* expression and IC10—* indicates *t*-test *P* value<0.005.

We then investigated the expression of *BCL11A* in other patient data sets including METABRIC^5^ and TCGA^8^, which between them have curated gene expression, copy number (CN) variation and clinical data from close to 3,000 patients^5^. Pathologically, we found that high *BCL11A* expression significantly correlated with TNBC pathology (Fig. 1a). At the molecular level, high *BCL11A* expression was also found to significantly correlate with the BLBC subtype in the METABRIC, TCGA and six other microarray data sets (Fig. 1b and Supplementary Fig. 1b). Quantitative reverse transcription PCR (qRT–PCR) analysis of *BCL11A* expression on a randomly selected subset of METABRIC tumours (all subtypes, *n*=230) validated the above expression data (Supplementary Fig. 2a). In addition, we also found that high *BCL11A* expression in METABRIC samples correlated with the recently described IC10 cluster of tumours (Fig. 1c), thus further supporting the concordance between the BLBC and IC10 classifications. Consistent with TNBC cases, high *BCL11A* expression was significantly correlated with a high histological grade (Supplementary Fig. 2b).

**Figure 2:**
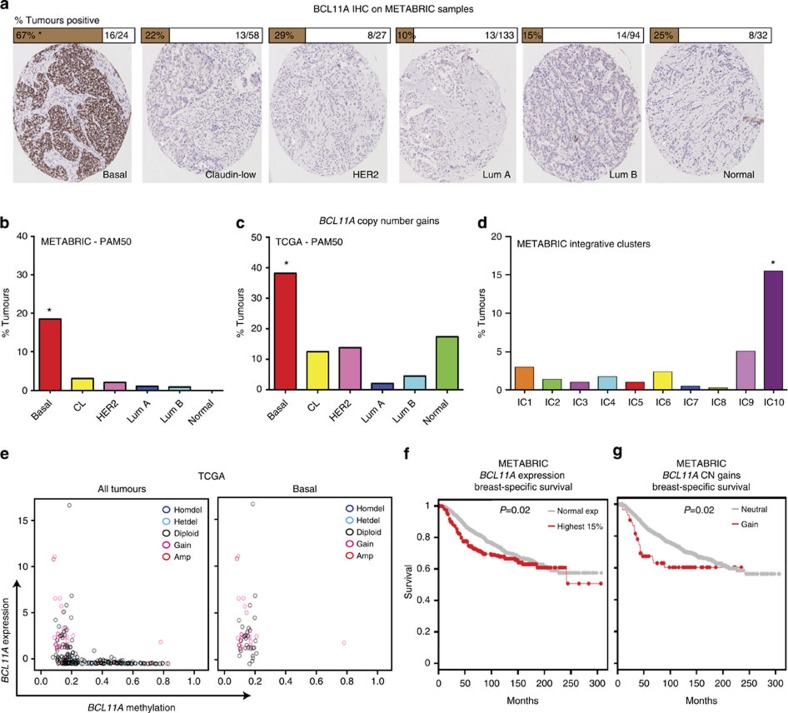
Genomic alterations at the *BCL11A* locus. (**a**) Images and scoring of BCL11A IHC staining on a subset of tumours from the METABRIC study (see Methods for scoring)—* indicated *χ*^2^-test *P*<0.0001. (**b**–**d**) Bar chart depicting the percentage of samples that harbour *BCL11A* CN gains in each subtype in both METABRIC and TCGA data sets—* indicates *χ*^2^-test *P*<0.0001. (**e**) Scatter plots showing the methylation status of the *BCL11A* locus in all tumours or basal only from the TCGA data set. The colour-coded legend indicated the BCL11A CN status for each tumour. (**f**–**g**) Kaplan–Meir plots showing the survival rate comparison between patients who have normal or high levels of *BCL11A* expression, or between patients with CN gains or without (neutral).

Furthermore, high *BCL11A* expression in BLBC cases was further validated by immunohistochemistry (IHC) on a subset of the METABRIC tumours (all subtypes, *n*=368. BLBC, *n*=24). Strong BCL11A immunostaining was predominantly found in BLBC (Fig. 2a). Out of 24 BLBC samples examined from this subset, 16 scored positive for BCL11A (Fig. 2a; details in Methods). In addition, samples stained positively in IHC also had higher RNA levels compared with those scored as negative (Supplementary Fig. 2c).

One mechanism for the induction of high *BCL11A* expression in BLBC cases could be CN aberrations. From ~2,000 breast cancer cases in METABRIC^5^, CN gains at the *BCL11A* genomic locus were identified in 62 patients (Supplementary Fig. 3a), which also correlates with high *BCL11A* expression (Supplementary Fig. 3b). Importantly, out of these 62 patients with CN gains, 39 were classified as BLBC, which account for 18.6% (39/210) of the total BLBC cases in METABRIC (Fig. 2b). Examination of the TCGA data set revealed that 38% (31/81) of BLBC samples have *BCL11A* CN gains, which is again significantly correlated with higher gene expression (Fig. 2c and Supplementary Fig. 3c). A similar result was also found when the METABRIC data was analysed using the integrative clustering, with 15.6% of IC10 samples having BCL11A CN gains (Fig. 2d).

Further analysis of the TGCA data set revealed that in BLBCs, the *BCL11A* locus is almost exclusively hypomethylated and this is correlated with high expression levels (Fig. 2e). There was also no correlation between *BCL11A* CNs and the methylation status. This result suggests that epigenetic changes at the *BCL11A* locus could be another mechanism that contributes to its high expression in BLBC. Given the strong correlation with TNBC, patients with either high expression or CN gains of *BCL11A* had poor survival rates compared with the rest of the cohort (Fig. 2f, g). A similar trend was also observed in four other patient data sets^17^,^18^,^19^,^20^ (Supplementary Fig. 3 f-i). In particular, patients with CN gains of *BCL11A* had a higher rate of relapse and metastasis and a lower rate of survival (Supplementary Fig. 3d-e). The utility of *BCL11A* expression/CN as a biomarker in the clinic thus warrants further investigation. Indeed, the future release of patient outcome for the complete TCGA cohort will aid in clarifying this finding.

### High levels of *BCL11A* promote tumour development

**Figure 3:**
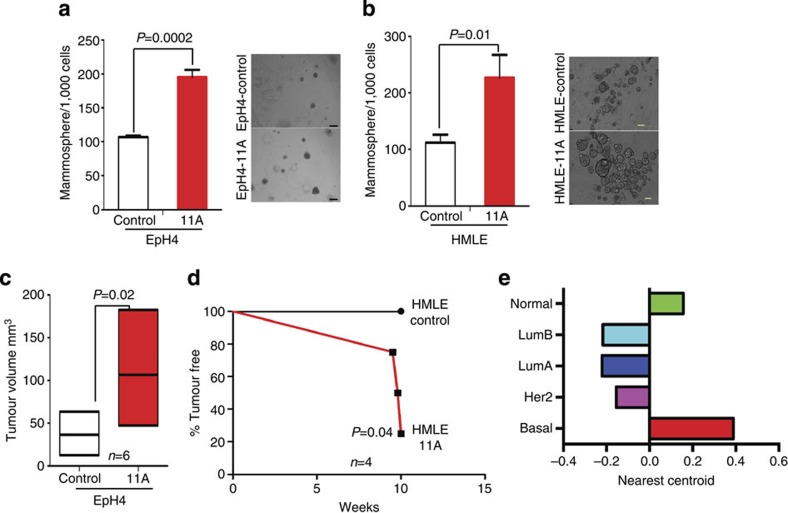
High levels of BCL11A enhance clonogenicity of mammary epithelial cells and promote tumourigenesis. (**a**) Comparison of colony numbers in matrigel from EpH4-11A cells or from the control cells. Data are presented as mean±s.d. (*n*=3). Image on the right are depicting EpH4-control and EpH4-11A mammospheres grown in Matrigel (scale bar, 100 μm). *P* value indicates student’s *t*-test. (**b**) Comparison of the number of floating mammospheres formed from human HMLE-11A cells or the control cells. Data are presented as mean±s.d. (*n*=3). Images on the right are of floating mammospheres formed by HMLE-control and HMLE-11A-expressing cell (scale bar, 200 μm). *P* value indicates student’s *t*-test. (**c**) Graph depicting the size difference between tumours at 6 weeks after injection of EpH4-control and EpH4-11A cells orthotopicaly into contralateral mammary fat pads. Data are presented as mean±s.d. (*n*=6). *P* value indicates student’s *t*-test. (**d**) Kaplan–Meier survival curve depicting the percentage of tumour-free mice injected with either HMLE-control or HMLE-11A cells (*n*=4). (**e**) Unsupervized clustering of the HMLE-11A tumours in the mouse with human tumours from the METABRIC study based on the PAM50 (ref. 3) gene expression. Nearest centroid correlation score is plotted against the various subtypes for all three tumours.

Although BCL11A is involved in rare B-cell lymphomas and is able to transform fibroblast cells *in vitro*^21^,^22^, the cellular and molecular mechanisms of BCL11A-mediated tumourigenesis remains unclear. To address this, we first tested whether *BCL11A* overexpression could promote the colony formation or tumour development in mammary epithelial cells. We overexpressed *BCL11A* in immortalized non-tumourigenic mouse EpH4 (ref. 23) or human HMLE^24^,^25^ cells (Supplementary Fig. 4a) and performed Matrigel and suspension mammosphere assays. Forced BCL11A expression in both EpH4 and HMLE (EpH4-11A and HMLE-11A) cells resulted in double the number of spheres compared with their respective control cells (Fig. 3a-b). Furthermore, mouse EpH4-11A cells injected orthotopically in cleared mammary fat pads of immune-compromized *NOD/SCID/IL2rγ*^*−/−*^ (NSG) mice^26^ formed larger and palpable tumours compared with control cells (*n*=6) (Fig. 3c and Supplementary Fig. 4b). Similarly, three out of four mice injected with HMLE-11A cells developed tumours within 8 weeks of injection (Fig. 3d and Supplementary Fig. 4c) suggesting that elevated levels of BCL11A promote tumour development. Moreover, gene expression analysis of these three tumours along with the 2,000 tumours from the METABRIC study clustered them with the BLBC subgroup (Fig. 3e).

### Knockdown of *BCL11A* reduces tumourigenicity of TNBC cells

**Figure 4:**
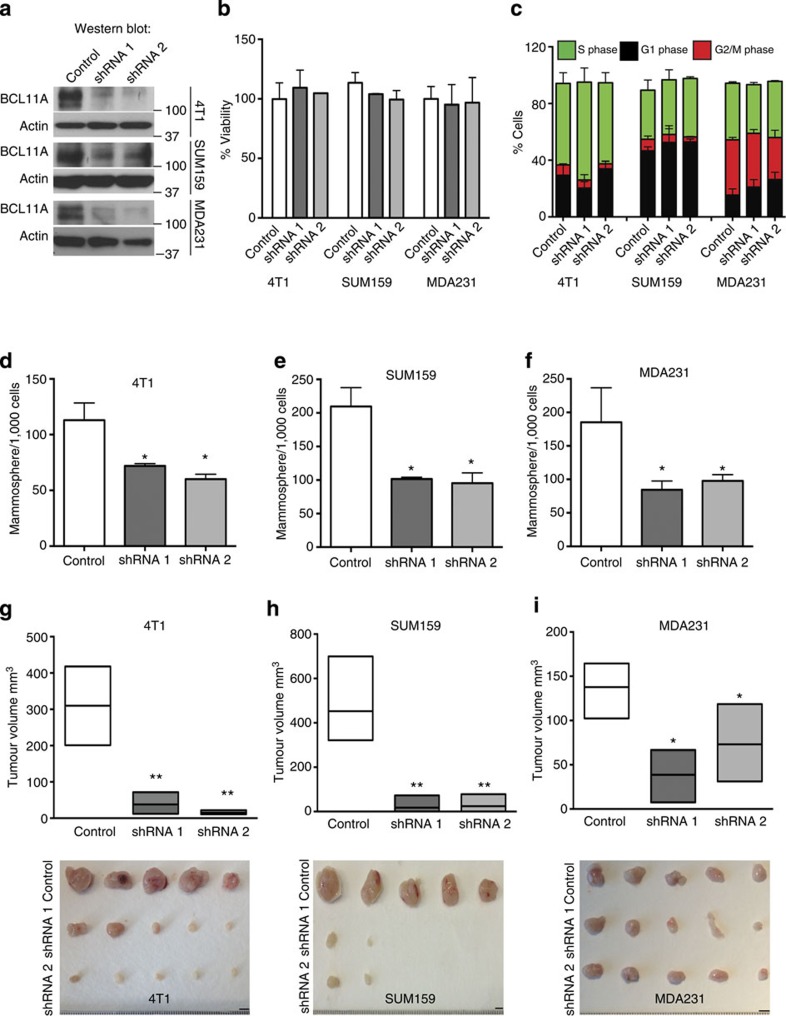
*BCL11A* knockdown in TNBC cells reduces tumour development. (**a**) Western blot showing BCL11A knockdown efficiency in 4T1, SUM159 and MDA231 cells transfected with control (scramble), shRNA1 or shRNA2 vectors. (**b**) MTS cell viability assay (see Methods) shows that BCL11A knockdown does not affect cell viability in culture. (**c**) EdU cell cycle analysis showing that Bcl11a knockdown does not affect cell cycle kinetics in all tested cell lines. Data are presented as mean±s.d. (*n*=3). (**d**–**f**) Comparison of colony numbers from control, shRNA1 and shRNA2 in 4T1, SUM159 and MDA231 cells. Data are presented as mean±s.d. (*n*=3). *P* value indicates student’s *t*-test. (**g**–**i**) Graph depicting the reduction in tumour size observed when shRNA1 or shRNA 2 transfected 4T1, SUM159 or MDA231 cells are injected subcutaneously into mice compared with control. Image under graph shows the actual tumours measured (scale bar, 5 mm). Unpaired *T*-test was performed on **d**–**i** and * indicates *P*<0.05 and ** indicates *P*<0.005.

Analysis of *BCL11A* expression in a panel of breast cancer cell lines revealed that *BCL11A* is highly expressed in TNBC lines but is undetectable in any of the luminal cell lines tested (Supplementary Fig. 5a). Next, we assessed if disrupting *BCL11A* expression could affect the clonogenic and oncogenic potential of the TNBC cell lines. To inactivate *BCL11A* in these cells, we performed shRNA knockdown experiments (Supplementary Fig. 5b) in the TNBC cell lines 4T1 (mouse), MDA231, SUM159 and HMLER (human). Knockdown of *BCL11A* had no significant impact on cell viability, cell cycle kinetics or cell death (Fig. 4a-c and Supplementary Fig. 5b,d). However, *BCL11A* knockdown significantly reduced the clonogenic capacity of all four cell lines (Fig. 4d-f and Supplementary Fig. 5c). To assess tumourigeneic potential, *BCL11A* knockdown cells were injected subcutaneously into NSG recipients. Robust tumours developed from the control 4T1, MDA231, SUM159 and HMLER cells within 25 days. In contrast, the *BCL11A* knockdown cells produced tumours of significantly reduced sizes (Fig. 4g-i and Supplementary Fig. 5c). Furthermore, primary and secondary limiting dilution transplantations of MDA231 control or shRNA1 cells revealed a reduction in the number of tumour-initiating cells during the secondary transplants from 1/123 to 1/667 (Supplementary Fig. 5e).

### *Bcl11a* is required for the development of DMBA tumours

**Figure 5:**
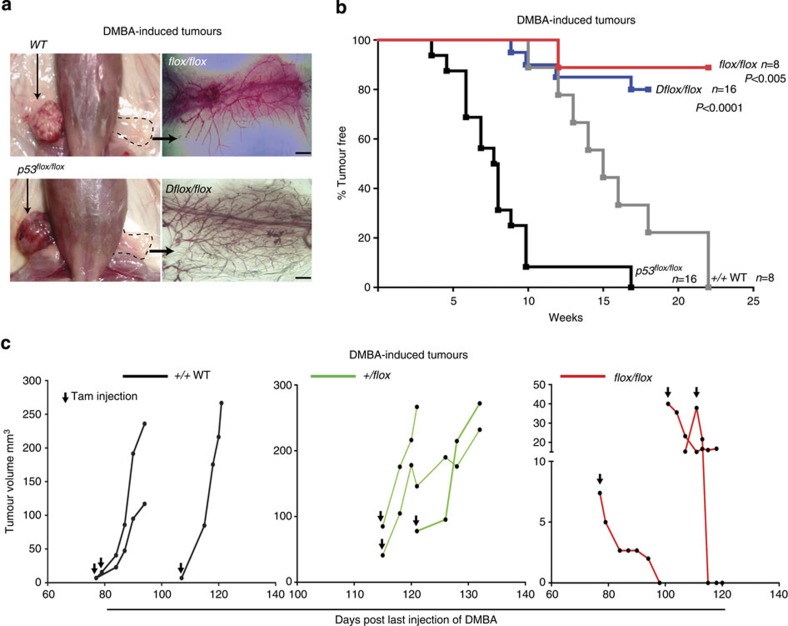
*Bcl11a* is required in DMBA-mediated tumourigenesis. (**a**) Mouse images and fat pads whole-mount fat pads of either WT, *flox/flox, p53flox/flox* or *Dflox/flox* mammary cells. (scale bar, 500 μm) (**b**) Quantification of the tumours with DMBA-mediated tumour development from the four depicted groups of engrafted cells over a period of 26 weeks. A log-rank (Mantel–Cox) test was used to compare the two groups and calculate the *P* value. (**c**) Tumour size quantification of DMBA-mediated tumours in WT, *+/flox* and *flox/flox* mice. Mice were checked regularly for tumour development and once detected, Cre activation was induced using three injections of tamoxifen (first day of injection is indicated by black arrow). Tumours size was then monitored for up to 20 days or until they reach a critical size.

To examine the role of BCL11A in mammary tumour development *in vivo*, we generated *Bcl11a* conditional knockout (cko) mice (referred to as *flox/flox*; Supplementary Fig. 6a), as germline deletion of *Bcl11a* causes neonatal lethality^27^ and crossed them to the inducible *Rosa26-CreERT2.* As a tumour model, we used the potent carcinogen DMBA (7,12-dimethylbenz(a)anthracene) in combination with medroxyprogesterone acetate (MPA) to promote TNBC-like tumours in the mouse^28^,^29^. To minimize the effects of *Bcl11a* deletion on non-mammary tissues, we transplanted mammary tissue from 8- to 12-week-old control (wild type) or *flox/flox* virgin female mice into contralateral cleared fat pads of female NSG mice followed by DMBA mutagenesis as illustrated in Supplementary Fig. 6b. By week 15, after the last dose of DMBA was administered, palpable tumours were visible in the mammary glands engrafted with the control mammary cells, but not with the *flox/flox* cells (Fig. 5a). By week 22 post DMBA treatment, all control cell engraftments (8/8) developed tumours compared with only one from *flox/flox* mammary cells (1/8) (Fig. 5b). qRT–PCR analysis of this tumour revealed expression of *Bcl11a* probably owing to incomplete Cre-*lox*P recombination (Supplementary Fig. 6c, sample T1). Also, qRT–PCR and IHC results revealed that tumours upregulated *Bcl11a* expression in response to DMBA-induced carcinogenesis (Supplementary Fig. 6c-d). These data thus reveal a requirement for *Bcl11a* in DMBA-induced mammary tumourigenesis.

To investigate Bcl11a oncogenic activity in the DMBA model further, we performed the DMBA mutagenesis experiment using *Trp53flox/flox*^30^ (*p53* single cko) or *Bcl11aflox/flox/p53flox/flox* (cko alleles for both *p53* and *Bcl11a* or *Dflox/flox*) mammary tissues. In the recipients transplanted with *Trp53flox/flox* cells, palpable tumours were detectable as early as 4 weeks after the last injection of DMBA, and most tumours were detectable by week 10 (Fig. 5b; *n*=16). However, deletion of *Bcl11a* together with *p53* in *Dflox/flox* mice severely delayed tumour development with only 4 out of 16 mice developeing tumours by week 17 (Fig. 5b). This result indicates that BCL11A is a potent oncogene and is required in concert with p53 for tumour development.

### *Bcl11a* is required for the maintenance of DMBA tumours

Although *Bcl11a* is important for DMBA-induced mammary tumour formation, it is more clinically relevant if it has functions in mammary tumour progression and maintenance. We thus performed the DMBA mutagenesis on WT, *flox/+* and *flox/flox* mammary epithelial cells before the induction of *Bcl11a* deletion. Only when mammary tumours were detected and measured, the mice were then injected with tamoxifen to induce *Bcl11a* deletion. As shown in Fig. 5c, deletion of *Bcl11a* caused a significant reduction in tumour size as soon as 5 days post deletion. On contrary, tumours from the control heterozygous donor cells continued to grow post tamoxifen injection (Fig. 5c). The requirement of *Bcl11a* in the established mouse mammary tumours is consistent with the decreased tumourigensis of *BCL11A* knockdown breast cancer cells and underscores its candidature for therapeutic development.

### *Bcl11a* is required for mammary stem and progenitor cells

**Figure 6:**
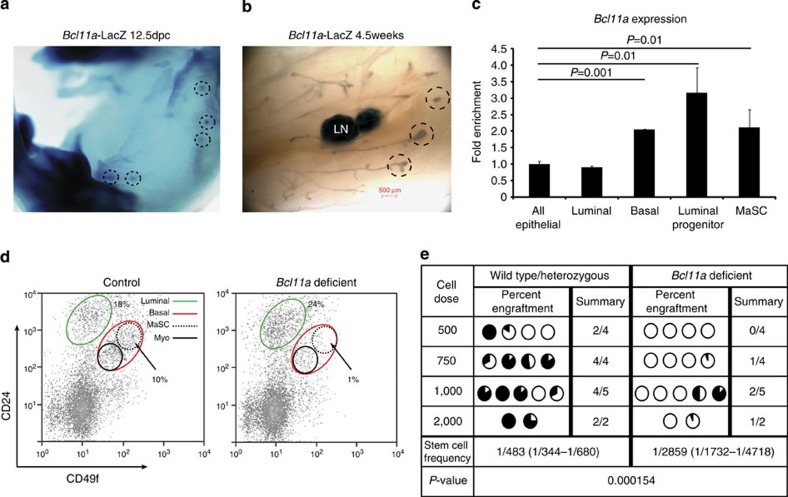
Expression and critical roles of Bcl11a in mouse MaSCs and progenitors. (**a**,**b**) X-gal staining of a 12.5dpc embryo and whole mount of the mammary gland from a 5-week-old *Bcl11a*-^*lacZ/+*^ female virgin mouse. The dashed circles highlight the mammary placodes and the terminal end buds. LN: lymph node. (scale bar, 500 μm) (**c**) qRT–PCR for *Bcl11a* in different mammary epithelial cell compartments that were fluorescence-activated cell sorting-purified using antibodies for CD24, CD49f and CD49b. Data are presented as mean±s.d. (*n*=3) and *t*-test was performed and the *P* values are displayed on the plot. (**d**) Depletion of the MaSCs-enriched population (CD24^med^CD49f^hi^, dashed lines) in the *Bcl11a*-deficient mammary gland detected by flow cytometric analysis. (**e**) Limiting dilution transplant (fat pad) assay showing severely compromized engraftment of *Bcl11a*-deficient MaSCs. Stem cell frequency calculation is described in the Methods.

To understand the biological function of Bcl11a in healthy mammary epithelial cells, we generated a *Bcl11a*-*lacZ* knock-in mouse to determine the temporal and spatial expression of *Bcl11a* in the mammary gland (Supplementary Fig. 7a). X-gal staining of the reporter embryos revealed that *Bcl11a* was expressed in the mammary placodes from 12.5dpc (Fig. 6a). At puberty, *Bcl11a* was expressed in the cap cells of the terminal end buds, a region thought to harbour stem cells^31^ (Fig. 6b). During adult mammary gland development, *Bcl11a* exhibited a dynamic expression pattern with a marked increase at early gestation and a gradual decline towards lactation and involution (Supplementary Fig. 7b). qRT–PCR analysis of RNA samples from several mammary epithelial compartments^32^,^33^ detected higher levels of *Bcl11a* expression in the luminal progenitors (CD49b^+^/CD24^hi^), the basal cells (CD49F^hi^/CD24^+^) and the mammary stem cell (MaSC) (CD49F^hi^/CD24^med^)-enriched population (Fig. 6c).

We next induced *Bcl11a* deletion and analysed the mammary epithelial fluorescence-activated cell sorting profile 3 weeks post deletion. The basal mammary epithelial cells from the *flox*/*flox* mice appeared to be depleted, and in particular the MaSC fraction (Fig. 6d). In addition, *Bcl11a* deletion caused a significant decrease in the number of luminal colony-forming cells (CFCs) (Supplementary Fig. 7c). To functionally demonstrate loss of MaSC activities upon *Bcl11a* deletion and to determine that the defects are cell-autonomous, we transplanted control and *flox/flox* cells at limiting dilution into cleared fat pads of NSG mice (see Methods). We found approximately sixfold reduction in stem cell frequency from 1/483 to 1/2859, in the *Bcl11a*-deficient mammary gland (Fig. 6e). Reduction of MaSCs and progenitors in the *Bcl11a*-deficient mammary gland was also reflected in the altered expression of the MaSC gene expression signature^34^ (Supplementary Table 1) (Supplementary Fig. 7e).

## Discussion

We have demonstrated here that the transcription regulator BCL11A is a novel breast cancer gene. By investigating cancer genomics data from ~3,000 patients (METABRIC and TCGA), *BCL11A* was significantly expressed at higher levels in TNBC and particularly in BLBC/IC10 tumours both at RNA and protein levels. Experimentally, we have shown that disrupting BCL11A expression in TNBC cell lines and in the mouse significantly reduced tumour development and maintenance. At the cellular level, *Bcl11a* is expressed and required in both MaSCs and luminal progenitor cells in the mammary gland. Lineage tracing experiments in the future will determine if *Bcl11a* is expressed in the recently identified lineage-restricted luminal and basal progenitor cells^35^ or in the bipotent MaSCs^36^. Importantly, given the recent implication of luminal progenitors as the ‘cell of origin’ of BLBC^37^,^38^, it will be important to ascertain if *Bcl11a* upregulation in luminal progenitor cells is one of the earliest steps in TNBC development.

In addition, it will be important to identify how *BCL11A* is transcriptionally regulated and what are its downstream targets in TNBC. In erythrocytes, KLF1 has been shown to affect *BCL11A* expression^39^, while in non-small cell lung cancer *MIR30A* has been suggested to regulate *BCL11A* expression^40^. We found no correlation between *KLF1* or *MIR30A* and *BCL11A* expression in the TCGA data set (Supplementary Fig. 8), suggesting that *BCL11A* regulation could be context dependent. In terms of downstream targets, in leukaemia, it has been shown that *BCL11A* abrogates p21 transcription possibly via direct regulation of *SIRT1* (refs 41, 42). Previous work from our lab also showed that in B cells, BCL11A induces *MDM2* expression, which is a negative regulator of p53 (ref. 43). However, the TCGA data does not indicate a strong correlation between *BCL11A* and *SIRT1* or *MDM2* expression at least in the tumour context (Supplementary Fig. 8). Therefore, identifying the putative *BCL11A* regulators and its downstream targets in the breast epithelial cells should clarify its molecular and cellular roles in TNBC.

In conclusion, through cancer genomics, *in vitro* assays, experimental xenograft models and mouse genetics, we have demonstrated in this study that *BCL11A* is a new breast cancer gene and a critical regulator in normal mammary epithelial development. These results warrant further investigation of *BCL11A* as a potential candidate for TNBC-targeted therapy.

## Methods

### Mouse strains and breeding

All experimental animal work was performed in accordance to the Animals (Scientific Procedures) Act 1986, UK and approved by the Ethics Committee at the Sanger Institute. *Bcl11a* bacterial artificial chromosomes (BACs) were identified from the 129/SvJ mouse BAC library (Sanger Institute) and used to generate the *Bcl11a-lacZ*- and *Bcl11a* cko-targeting vectors. For *Bcl11a-lacZ* reporter, targeting construct (Supplementary Fig. 7a) was generated based on the recently published strategy^44^. For the *Bcl11a* cko mouse, targeting construct (Supplementary Fig. 6a) was generated based on the original recombineering strategy^45^. Gene targeting in embryonic stem (ES) cells and chimera production were performed according to the standard procedures. The *Bcl11a* cko line was then crossed to the Rosa26-Cre-ERT2 mouse line described previously^46^. The *p53* cko line was described previously^30^. Homozygous *p53* cko mice were crossed to the *Bcl11a/Cre-ERT* line described above and the F1 generation was mated to generate mice doubly conditional for *Bcl11a* and *p53*. Genotyping was confirmed using the primers listed in Supplementary Table 2. Cre activation was mediated by three injections of 1 mg tamoxifen per mouse over 3 days.

### Mammary epithelial cell isolation and analysis

Mammary epithelial cells were dissociated using a mixture of collagenase (Roche) and hyaluronidase (Sigma), and cells were stained using the following primary antibodies: biotinylated anti-CD45 (clone 30-F11; eBioscience, 1:500), anti-Ter119 (clone Ter119; eBioscience, 1:500) and anti-CD31 (clone 390; eBioscience, 1:500); anti-CD24-R-phycoerythrin (PE; clone M1/69, eBioscience, 1:500), anti-CD49f-Alexa Fluor 647 (AF647; clone GoH3, eBioscience, 1:100), anti-CD49b-Alexa Fluor 488 (AF488; clone HMa2; eBioscience, 1:500) and Sca1-Alexa Fluor 647 (AF647; clone D7, eBioscience, 1:500). Secondary antibodies used: Strepavidin-PE-Texas Red (PE-TR; Molecular Probes, 1:500). Apoptotic cells were excluded by elimination of propidium iodide-positive cells. Flow cytometric analysis was done using CyAN ADP (DakoCytomation) and all sorts were performed using MoFlo (DakoCytomation) and gates were set to exclude >99.9% of cells labelled with isoform-matched control antibodies conjugated with the corresponding fluorochromes. For whole-mount analysis, abdominal glands (no. 4) were spread out using forceps on a glass slide and incubated in Carnoy’s fixative overnight. The slide was washed in water and placed in carmine alum (Sigma) stain overnight. The slide was again washed with ethanol and cleared in Xylene for 1 day before documentation. For histological analysis, abdominal glands were fixed in 4% formaldehyde in PBS for 24 h at room temperature. The glands were transferred to 70% ethanol and stored at −20 °C until embedding and sectioning. All tissues were embedded in wax and sectioned at 5 μm before being stained with haematoxylin and eosin.

### Mammary CFC assay

For colony-forming assays, the medium used was (human) NeuroCult NS-A Proliferation Medium (StemCell) supplemented with 5% fetal bovine serum, 10 ηg ml^−1^ epidermal growth factor (Sigma), 10 ng ml^−1^ basic fibroblast growth factor (Peprotech) and N2 Supplement (Invitrogen); the cultures were maintained at 37 °C/5% CO_2_ for 7 days; then fixed using ice-cold acetone/methanol (1:1) and visualized using Giemsa staining (Merck). Lin^−^CD24^hi^CD49b^+^ luminal progenitors from the *flox/flox* mammary gland were sorted and plated with irradiated feeders in colony-forming assay medium for 6 days before the number of mammary CFCs was enumerated.

### Transplantation of mammary epithelium

Mammary epithelial cells (basal fraction) from tamoxifen-injected and non-injected *flox*/*flox* or *flox/+* mice were sorted based on CD24/CD49f and transplanted in limiting doses (500/750/1,000/2,000 cells) into cleared fat pads of 3-week-old NSG females. In each case, non-injected and tamoxifen-injected epithelial cells were engrafted into contralateral glands of the same recipient mice. The recipient mice were impregnated 3–6 weeks after transplant and outgrowths produced were dissected, stained with carmine and scored. Stem cell frequency was calculated using L-Calc (StemCell Technologies).

### DMBA/MPA tumourigenesis protocol

Mammary fragments were transplanted into cleared fat pads of 3-week-old NSG mice. At the time of surgery, the MPA slow release pellet (Innovative Research of America) was also implanted subcutaneously. The mice were allowed to recover for 2 weeks and then *Bcl11a* deletion was induced using three injections of tamoxifen. One week after deletion of *Bcl11a*, 1 mg of DMBA (Sigma) was administered orally; this was followed by three further doses of 1 mg of DMBA over 3 weeks. Mice were then examined weekly for tumour incidence and killed when tumours reached the legal limit.

### Transfection and mammosphere assays

EpH4 (gift from Professor Christine Watson) and MDA231 (ATCC) cells were cultured to confluence in 1:1 DMEM:F12 (Invitrogen) media containing 10% fetal calf serum (FCS; FetalcloneIII, Clonetech). 4T1 (ATCC) cells were cultured in Roswell Park Memorial Institute (RPMI) media (Invitrogen) containing 10% FCS, and SUM159 cells (gift from Dr Charlotte Kuperwasser) were cultured in Ham's F12 (Sigma), 5% FCS, insulin (5 μg ml^−1^, Sigma) hydrocortisone (1 μg ml^−1^, Sigma) and 1 × Penicillin Streptomycin Glutamine (PSG) (Gibco). HMLE and HMLER cells (gift from Professor Robert Weinberg) were cultured in complete HuMEC media (Invitrogen). The control or the *Bcl11a* overexpression piggyBac vectors were delivered into cells using the Amaxa Basic Nucleofactor Kit for primary mammalian epithelial cells (Lonza) according to the manufacturer’s recommendations. Transfected cells were maintained at 37 °C/5% CO_2_ for 48 h. Cells were then cultured in puromycin (1–5 μg ml^−1^) for 48 h to allow for selection. To induce BCL11A expression in EpH4 and HMLE cells, doxycycline (Clonetech) was used at a final concentration of 1.0 μg ml^−1^. Floating or Matrigel-embedded mammosphere were cultured and passaged as previously described^47^ in ultra-low attachment plates (Corning).

### RNA knockdown

*BCL11A* shRNA sequences were obtained from the TRC consortium^48^ (TRCN0000033449 and TRCN0000033453) were cloned into piggyBac transposon vector (PB-H1-shRNA-GFP). 4T1, SUM159, MDA231 and HMLER cells were transfected with 4.0 μg of piggyBac vector using Amaxa Basic Nucleofactor Kit for primary mammalian epithelial cells (Lonza) and GFP^+^ cells were sorted/analysed 24–48 h later.

### RNA extraction and real-time PCR analysis

RNA from sorted cells was extracted using PicoPure RNA isolation kit (Molecular Devices) according to the manufacturer’s instructions. RNA from mammary tissue and cell lines was extracted using Tri-Reagent (Invitrogen) according to the manufacturer’s instructions. Complementary DNA (cDNA) was synthesized from 1 to 2 μg of total RNA using the Transcriptor Reverse Transcription cDNA Synthesis Kit (Roche). RT–PCR was performed using Hi-Fidelity Extensor mix (Thermo) using primers listed in Supplementary Table 2. Quantitative real-time PCR detection of cDNA was performed using SYBR Green Master Mix (Sigma, ABI and Invitrogen) according to supplier’s recommendations. The real-time PCR reactions were run in ABI-7900HT (Applied Biosystems) in triplicate. Primers used for real-time PCR on mouse samples were designed using PrimerBank^49^ website ( http://pga.mgh.harvard.edu/primerbank/) and listed in Supplementary Table 2. All primers were purchased from Sigma-Aldrich. For real-time PCR on human samples Taqman gene expression probes (Life Technologies) were used.

### Cell cycle analysis

A total of 150,000 control or *BCL11A* knockdown cells were seeded in six-well plates and allowed to recover for 48 h. Cells were then incubated with 5 μM Edu (Invitrogen) for 1 h. Cells were fixed and assayed using the EdU flow cytometery detection kit (Invitrogen) following the manufacturer’s instructions.

### Annexin v assays

A total of 100,000 control or *BCL11A* knockdown cells (in triplicates) were seeded in six-well plates and allowed to recover for 48 h. Cell were then collected and stained using the Annexin-V-AF647 (BioLegend) following the manufacturer’s instructions, and cells were then quantified using fluorescence-activated cell sorting.

### Cell viability assay

A total of 1,000 control or *BCL11A* knockdown cells (in triplicates) were seeded in 96-well plates and allowed to recover for 48 h. Cells were then incubated with CellTiter Aqueous One Solution (Promega) for 4 h following the manufacturer’s instructions. Absorbance was then measured at 490 nm using a plate reader (Bio-Rad).

### Western blotting and IHC

Protein samples were prepared as described previously^14^ and probed using anti-Bcl11a (Abcam, Clone 14B5, 1:1000) and Actin (Cell Signalling, 1:10000). For IHC analysis, BCL11A (Abcam (14B5, 1:50); CK14 (Abcam; 1:100) and ERα (SCBT; 1:50) were used. Staining was detected using AF488- or Cy3-conjugated secondary (Sigma) and bisbenzimide-Hoechst 33342 (Sigma). Fluorescence microscopy was carried out using a Zeiss Axiophot microscope equipped with a Hamamatsu Orca 285 camera, with images visualized, captured and manipulated using Simple PCI 6 (C Imaging Systems). The hematoxylin- and eosin-stained samples were visualized on a LEICA light microscope, while the mouse mammary gland whole mounts were visualized using the LEICA MZ75 light microscope.

### Microarray analysis

The intensity value for each probe set was calculated and the average of each gene was computed before the data analysis. For the quality control (QC) step, a set of intensity value of control genes were examined. All data were normalized and scaled by Partek Genomic Suite 6.4. Principal components analysis was performed to show the distribution of samples, eliminating outliers. Differentially expressed genes were selected by one-way analysis of variance by the factor of KO versus wild type, *P* value<0.08. Hierarchical clustering of selected genes was performed to show the expression pattern. The resulting genes then underwent a pathway analysis (GeneGO: http://www.genego.com) to determine the biological significance of the data.

### Xenograft tumourigenesis assays

One hundred thousand EpH4, HMLE, 4T1, MDA231, SUM159 or HMLER cells were suspended in 25% Matrigel (BD Biosciences) and HBSS, and injected into either cleared contralateral number 4 mammary fat pads of 3-week-old female mice or subcutaneously in 6–12-week-old female NSG mice. For secondary transplants, tumours were dissociated using collagenase/hyloronase mix (Roche) for 16 h and viable cells were counted and injected into NSG recipient mice at the indicated doses.

### METABRIC analysis

Matched DNA and RNA were extracted for tumours. CN analysis was performed using the Affymetrix SNP 6.0 platform. The arrays were pre-processed and normalized using CRMAv2 (ref. 50) method from aroma.affymetrix. Allelic-crosstalk calibration, probe sequence effects normalization, probe-level summarization and PCR fragment length normalization were performed for each array. The intensities obtained were normalized against a pool of 473 normals for the samples with no matched pair or against their matched normal when available (258 samples). The log-ratios were then segmented using the circular binary segmentation algorithm^51^ in the DNAcopy Bioconductor package^52^. Then, callings into five groups (homozygous deletion, heterozygous deletion, neutral CN, gain (>2) and amplification >3) were made using thresholds based on the variability of each sample and their proportion of tumoural cells. RNA analysis was performed using Illumina HT-12 v3 platform and analysed using beadarray package^53^. BASH^54^ was used to correct for spatial artifacts. The bead-level data were summarized and a selection of suitable probes based on their quality was done using the re-annotation of the Illumina HT-12v3 platform^55^. The samples were classified into the five breast cancer subtypes using PAM50 (ref. 56), but only those genes with a probe with perfect annotation on the chip were considered. A mixture model was used to classify BCL11A expression into low and high values^57^,^58^.

### TCGA data analysis

All TCGA data and figures were accessed, analysed and generated using the cBio Cancer Genomics Portal^59^. All data included in this manuscript is in agreement with the TCGA publication guidelines.

### METABRIC IHC analysis

A subset of patients enroled in the METABRIC study with tumour samples represented in tissue microarrays (TMAs) were included for the detection of BCL11A protein expression by IHC. TMAs were constructed from formalin-fixed paraffin-embedded tumour blocks as previously described^60^. Each tumour was represented by a single 0.6-mm tissue core. A total of 439 tumours were included arising from 436 patients (three were synchronous tumours arising in the contralateral breast). CN and gene expression data was available for 368 of these tumours for correlative analyses. Three micrometre TMA sections were dewaxed in xylene and rehydrated through graded alcohols. IHC was conducted using a BondMaX Autoimmunostainer (Leica, Bucks, UK). Antigen retrieval was achieved by heating TMA sections in pH 6 citrate buffer for 20 min. Primary mouse monoclonal (clone 14B5) antibody bound to BCL11A (ab19487, AbCam) was diluted to 1:200 and detected using a BOND Polymer detection kit (Leica) and signal developed with 3,3′-diaminobenzidine (DAB). Stained TMA sections were digitized using the Ariol (Genetix Ltd, Hampshire, UK) platform for scoring by a pathologist (H.R.A.). The ordinal Allred scoring system was used for assessing the amount of staining present in tumour cells accounting for the intensity (0=no staining, 1=weak, 2=moderate and 3=strong) and proportion (0=0%, 1=<1%, 2=1–10%, 3=11–33%, 4=34–66% and 5=>66%) of stained cells, finally producing a summed score (intensity+proportion=Allred score) between zero and eight. Analogous to clinical practice for estrogen receptor (ER), tumours with an Allred score of >2 were deemed positive for BCL11A expression and comparison with molecular subtypes was made using Pearson’s *χ*^2^ test.

### Statistical significance

All *P* values were calculated using Student’s *t*-test unless otherwise indicated in the figure legends.

## Additional information

**How to cite this article:** Khaled, W.T. *et al*. *BCL11A* is a triple-negative breast cancer gene with critical functions in stem and progenitor cells. *Nat. Commun.* 6:5987 doi: 10.1038/ncomms6987 (2015).

**Accession codes**: Microarray data have been deposited in the Gene Expression Omnibus database with the accession number GSE63386.

## Supplementary Material

Supplementary Information Supplementary Figures 1-9 and Supplementary Tables 1-2
