# Supplementary material for: BCL11A is a triple-negative breast cancer gene with critical functions in stem and progenitor cells
Source: Nat Commun. 2015 Jan 9;6:5987. doi: 10.1038/ncomms6987 (PMC4338552; doi:10.1038/ncomms6987)
Supplement: Supplementary Information — Supplementary Figures 1-9 and Supplementary Tables 1-2 [file ncomms6987-s1.pdf]

Supplementary Figure. 1

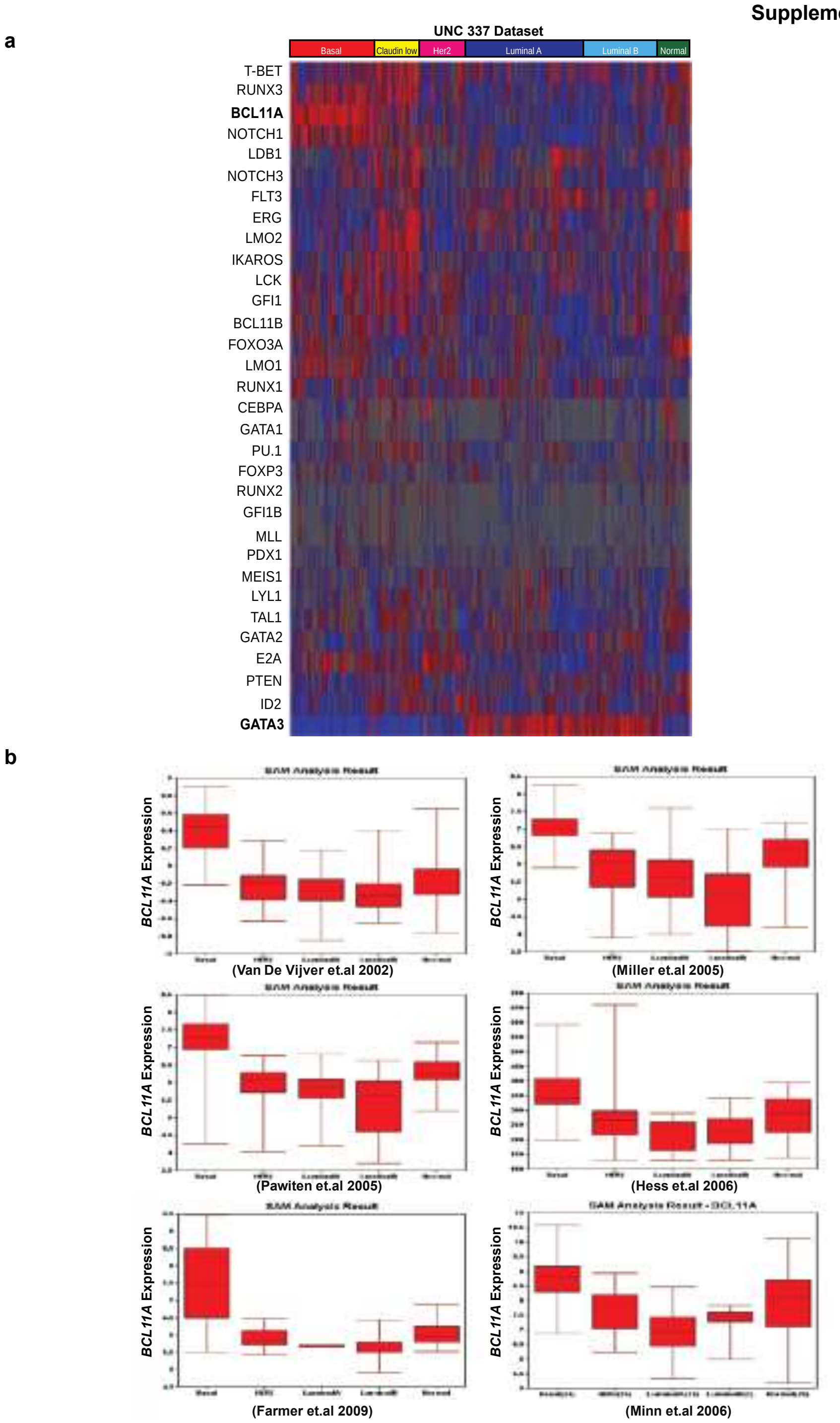

Supplementary Figure 1. Expression analysis of *BCL11* in various published microarray datasets
(a) Heatmap showing the expression analysis of a list of selected key hematopoiesis genes in 337 breast cancer patients [6] across different breast cancer molecular subtypes. **(b)** Analysis of *BCL11A* expression in the six microarray datasets from the ICR ROCK database. Box plots showing high levels of *BCL11A* in the basal subtype of breast cancer. All 6 data sets had *BCL11A* expression called at the basal subgroup and had a q-value less than 0.05.

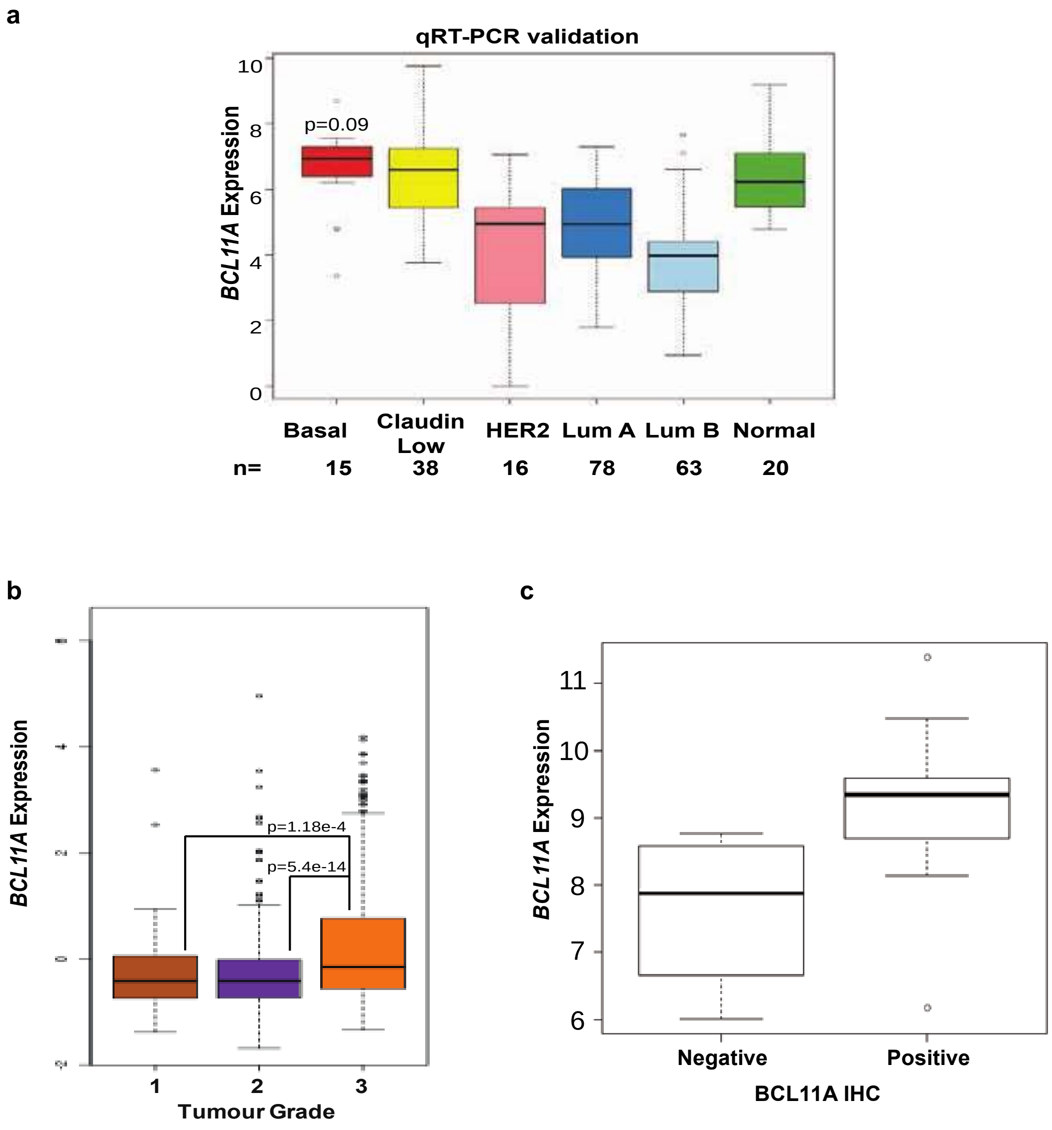

Supplementary Figure 2. Systematic analysis of *BCL11A* expression in the METABRIC dataset

(a) qRT-PCR analysis of *BCL11A* in 230 randomly selected patients from the METABRIC dataset. p-value refers to t-test comparing the Basal subtype vs. all other subtypes. (**b**) Correlation of *BCL11A* expression and the high clinical grades (1, 2, and 3) in patients from the METABRIC study **(c)** Correlation between BCL11A protein staining and the RNA levels. T-test was performed and the p-values are displayed on each panel.

Supplementary Figure. 3

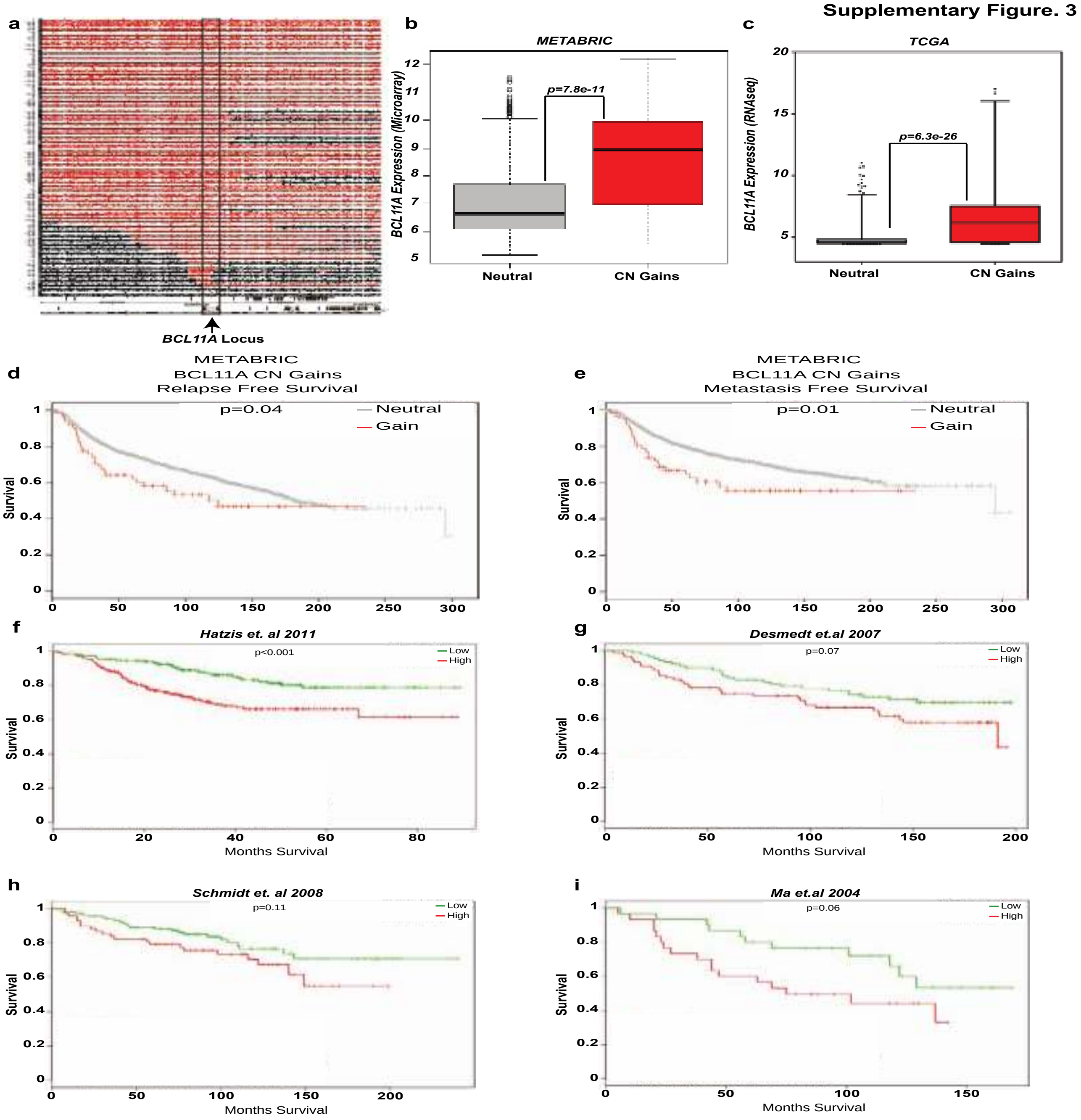

Supplementary Figure 3. Analysis of *BCL11A* copy number alterations in the METABRIC and TCGA datasets **(a)** Array comparative genomic hybridization (aCGH) plots showing CN gains at the *BCL11A* locus. A minimum region of CN gains can be defined around the *BCL11A* locus. **(b-c)** High *BCL11A* expression in patients with CN gains at the *BCL11A* locus in both the METABRIC and TCGA datasets. T-test was performed and the p-value is displayed on the plots. **(d-e)** Kaplan-Meir plots comparing relapse or metastasis survival rates between patients with *BCL11A* CN gains or those without. **(f-i)** Kaplan-Meier plots depicting the survival of *BCL11A* high vs. low patients in four independent datasets. Data was generated using the ICR ROCK database.

Supplementary Figure. 4

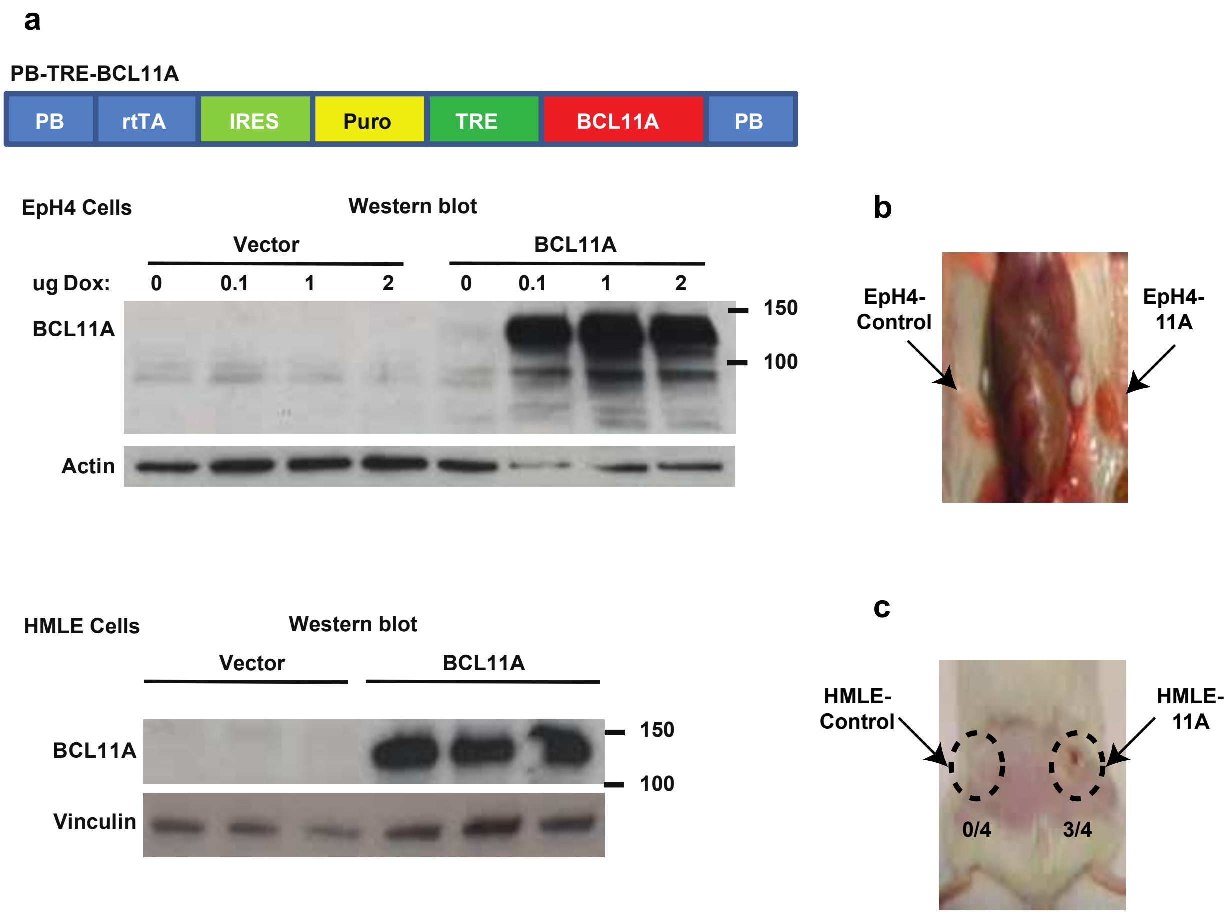

Supplementary Figure 4. *BCL11A* overexpression promotes colony formation and induces tumour development **(a)** Schematic diagram of the *BCL11A* overexpression vector used in this study. The expression cassette is cloned in a *piggyBac* transposon and is delivered to cells by the *piggybac* (PB) transposition. Western blot shows *BCL11A* transgene expression in EpH4 and HMLE cells. **(b-c)** Images of tumours formed by EpH4-11A and HMLE-11A cells.

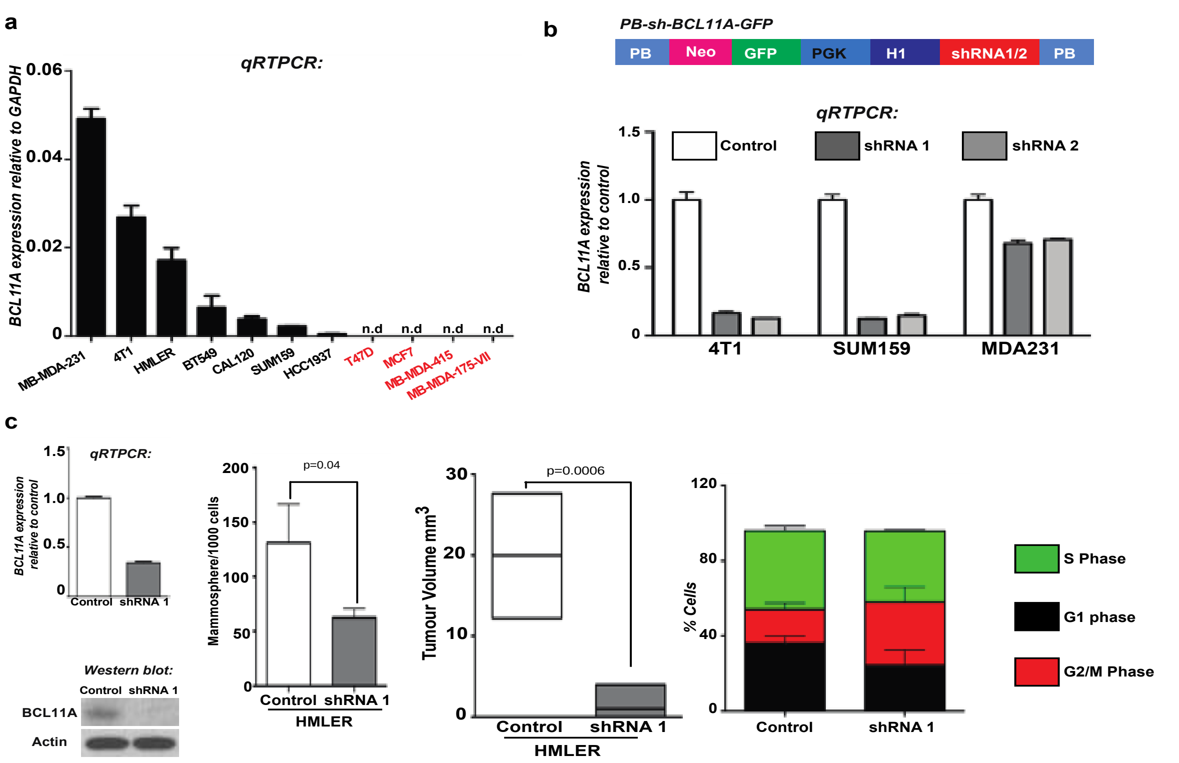

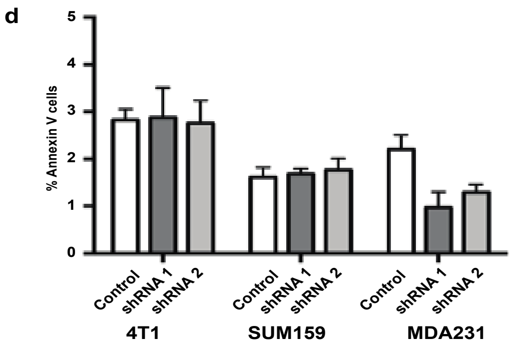

e

MDA231

Cell dose	Primary Control	Primary shRNA1	Secondary Control	Secondary shRNA1
10000	4/4	4/4	3/3	3/3
1000	4/4	4/4	4/4	4/4
500	4/4	4/4	5/5	4/5
100			5/5	1/5
TIC frequency			1/123	1/667
p value			*0.00102*	

Supplementary Figure 5. Knockdown of *BCL11A* in breast cancer cells suppresses tumour development
(a) qRT-PCR analysis of *BCL11A* expression in a panel of breast cancer cell lines. The cell lines in black font are TNBC and the cell lines in red font are luminal. n.d. indicates "not detectable". **(b)** Top: Schematic of the *BCL11A* shRNA knockdown vector in the PB transposon. Bottom: qRT-PCR on 4T1, SUM159 and MDA231 cells transfected with either the control (Scramble shRNA) or the *BCL11A* shRNA 1 or 2 vectors, Data are presented as mean ± SD (n=3). **(c)** *BCL11A* knockdown efficiency, mammosphere quantification, EdU cell cycle analysis and tumour data from HMLER cells transfected with control and shRNA1 vectors. Data are presented as mean ± SD (n=3). (**d)** Annexin V analysis of control or shRNA cells show that BCL11A knockdown has no impact on cell death in tissue culture. **(e)** Primary and secondary limiting dilution analysis of MDA231 control or shRNA1 cells reveals a reduction in the number of TIC in knockdown cells during secondary transplants. TIC frequency and p-value were calculated using calculated using L-Calc (see methods).

Supplementary figure 6

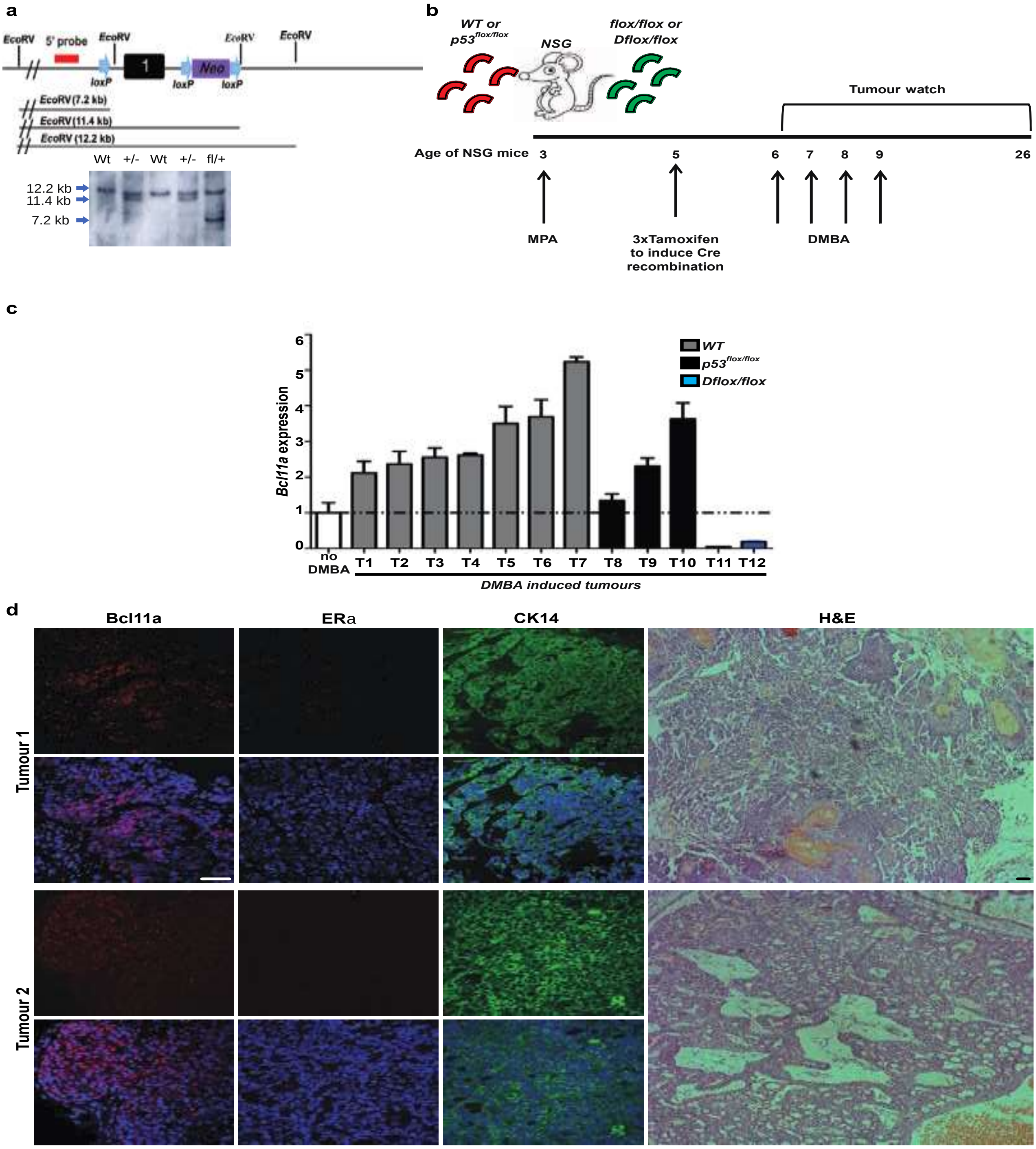

Supplementary Figure 6. Generation of *Bcl11a* conditional knockout mouse

Schematic diagram of the *Bcl11a* conditional knockout allele used in this study. Exon 1 of *Bcl11a* is flanked by *lox*P sites for deletion. Southern analysis on the right shows detection of the conditional knockout allele in mice. WT: wild type allele. fl: conditional knockout allele. **(b)** Experimental plan for the DMBA/MPA mediated tumourigenesis protocol. Mammary fragments from WT, *flox/flox, p53flox/flox and Dflox/flox* mice were transplanted into cleared fat pads of 3-weeks old NSG mice. At the time of surgery the medroxyprogesterone acetate (MPA) slow-release-pellet was also implanted subcutaneously. The mice were allowed to recover for two weeks and then *Bcl11a* deletion was induced using three injections of tamoxifen. One week after deletion of *Bcl11*a, DMBA was administered orally; this was followed by three further doses of DMBA over three weeks. Mice were then examined weekly for tumour incidence. **(c)** qRT-PCR showing the expression levels of *Bcl11a* in DMBA induced tumours on WT, *p53flox/flox* and *Dflox/flox* genetic backgrounds. *Bcl11a* expression is normalised to total mammary epithelial cell expression not treated with DMBA. Data are presented as mean ± SD (n=3). **(d)** IHC analysis of two DMBA induced tumours showing positive staining for BCL11A, CK14 and negative staining for ERα. (Scale bar = 40μm)

Supplementary figure 7

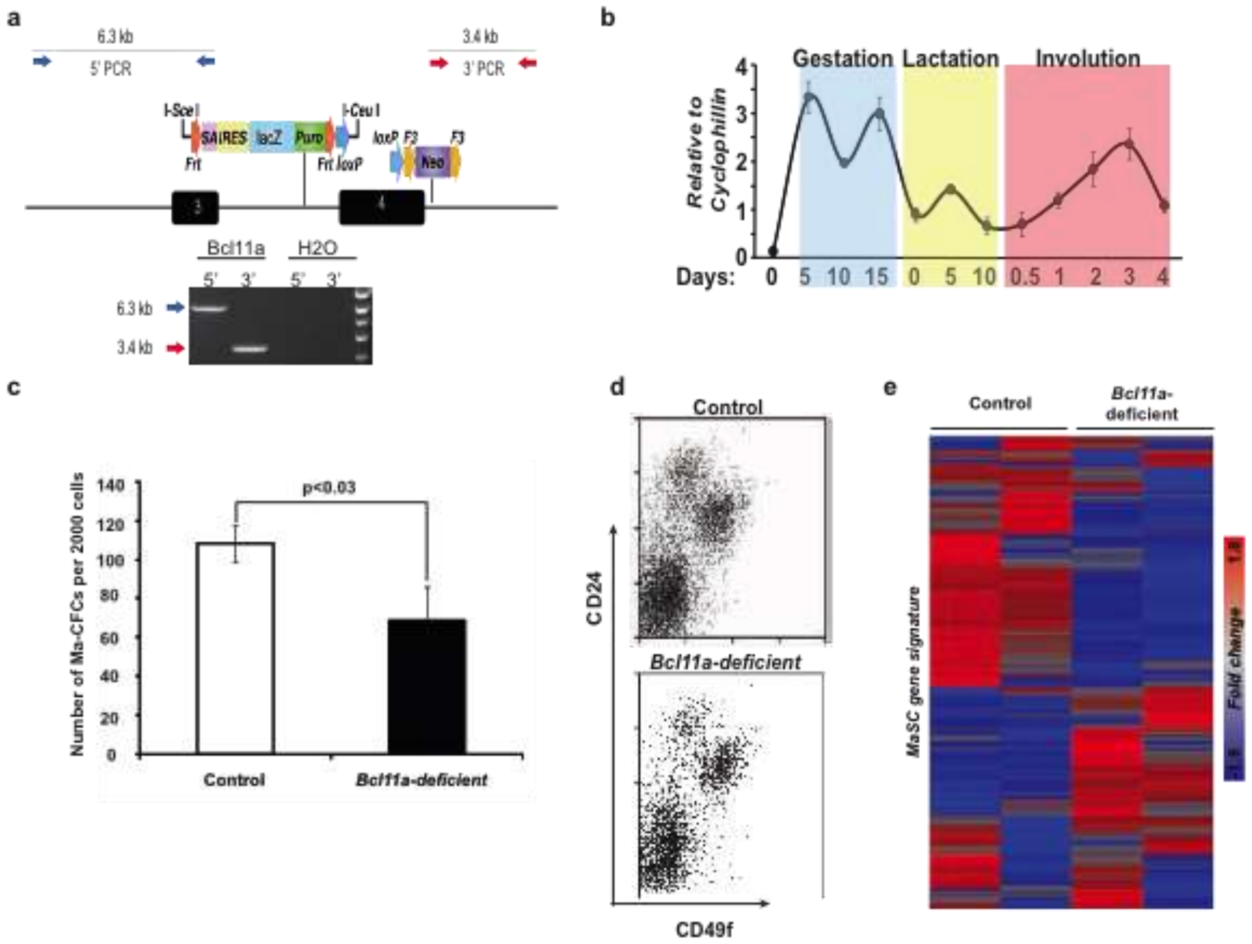

Supplementary Figure 7. Expression of *Bcl11a* in mouse mammary epithelial cells

(a) Schematic diagram of the *Bcl11a-lacZ* allele. The *SA-IRES-lacZ* cassette was targeted to intron 3 of the *Bcl11a* locus. (b) Expression of Bcl11a at different developmental stages of mouse mammary gland development detected by qRT-PCR. Data are presented as mean ± SD (n=3). **(c)** Significant decrease of Ma-CFCs upon *Bcl11a* deletion. Error bars denote standard deviation. Data are presented as mean ± SD (n=3). T-test was performed and the p-values are displayed on the graph. **(d)** FACS profiles of the control (*flox/+*) and the mutant (*flox/flox*) mammary epithelial cells one week post tamoxifen injection, the time point at which cells were sorted for the fat-pad transplantation and microarray experiments. Most epithelial cell populations are still present at this stage. **(e)** Gene expression analysis of *Bcl11a*-deficient MaSCs focusing on stem cell signature genes. The full gene lists are in Supplementary Tables 1. *Bcl11a* deletion in the mouse reduces expression of genes that are normally expressed in stem cells whereas *BCL11A* overexpression upregulates these genes

Supplementary Figure. 8

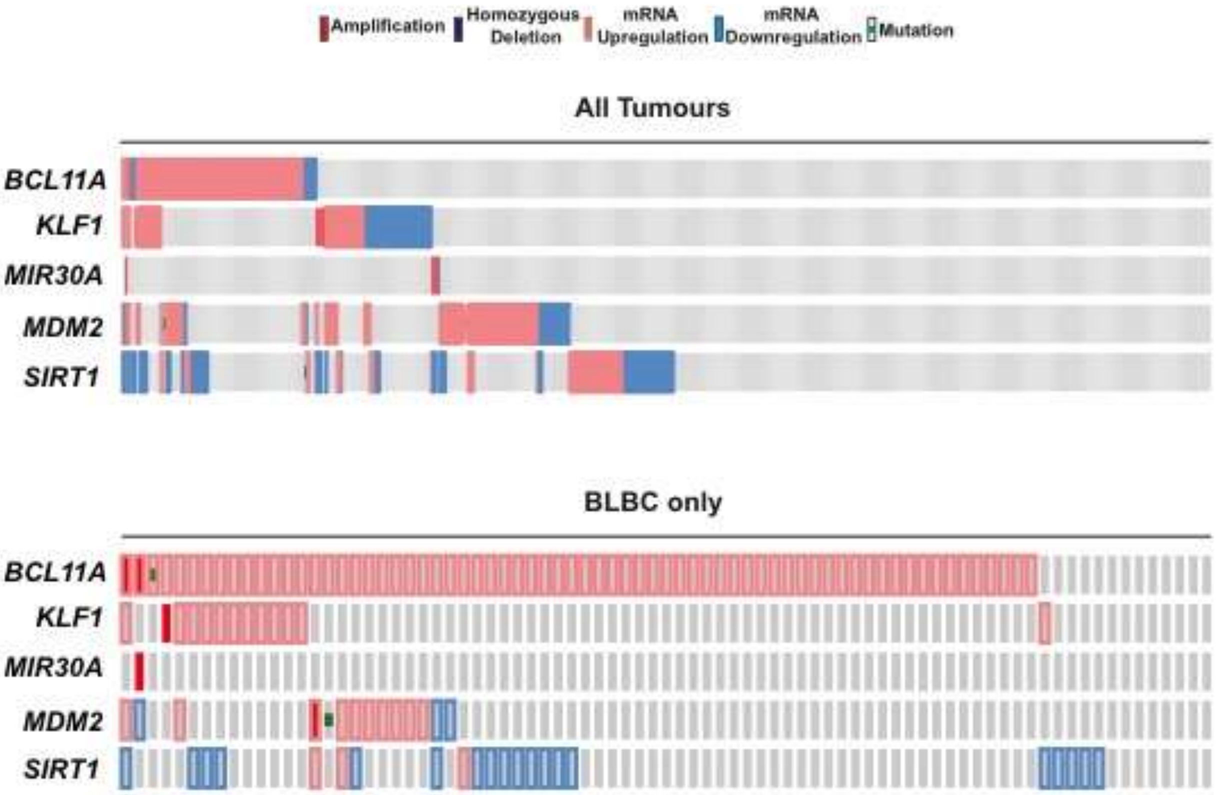

Supplementary Figure 8. TCGA olancoprint data depicting the lack of correlation between *BCL11A* expression and known BCL11A targets and in regulators in other cell types

Supplementary Figure. 9

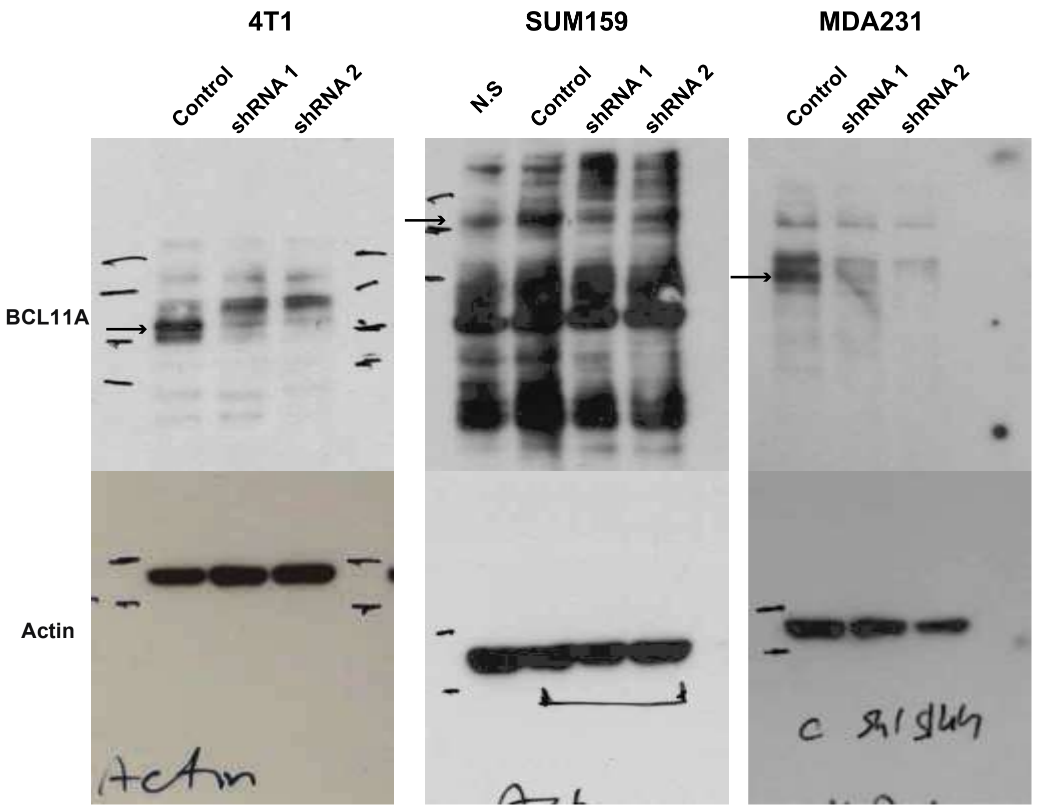

Supplementary Figure 9. Un-cropped Western blots corresponding to the cropped images in Figure 4a. N.S. indicates a non-specific lane. Arrows indicate BCL11A band.

Supplementary Table 1		
Column ID	p-value(KO vs. WT)	Fold-Change(KO vs. WT)
GLYCAM1	0.0115838	1046.9
SYVN1	0.0436103	37.7442
NDRG1	0.0380901	13.5986
ETS2	0.0494484	5.46734
TCFAP2C	0.0163037	4.536
VEGFA	0.00740769	3.2764
LMO4	0.0281296	1.5472
ETF1	0.0402482	1.51407
GSK3B	0.0196512	1.26023
TNFRSF11A	0.00204748	1.22492
MPP6	0.0477028	1.13048
NFAT5	0.0305385	1.0906
MYSM1	0.0254741	1.06206
PDIA3	0.0715047	53.0613
HERPUD1	0.0719123	47.5875
LDHA	0.020959	13.9667
IER3	0.0550579	8.0296
OAT	0.0180899	7.65035
MBNL2	0.0177637	7.63077
HSPA9	0.0493653	6.8779
RN18S	0.0736481	6.08801
2810423A18RIK	0.0591128	5.63919
SLC35B1	0.0371218	5.54558
ALDH1L2	0.0652081	5.3942
H47	0.0615762	4.90228
RPN2	0.0684499	4.45927
SERPINH1	0.0665414	4.39015
PTTG1IP	0.035419	4.28898
PDLIM1	0.0670925	4.11028
MYD116	0.0286962	4.06994
DEB1	0.0496911	4.05518
TMBIM6	0.0195185	3.8657
LOC100041569	0.0710527	3.76456
4732462B05RIK	0.0121286	3.60503
NSEP1	0.0392899	3.33886
LOC666676	0.0331728	3.29366
LOC100042427	0.0704957	3.24452
ATF4	0.0256431	3.22727
VCP	0.0612251	3.10911
DNAJB9	0.0424506	3.04215
GARS	0.0343635	3.02095
LOC677551	0.0748016	3.00498
TMED9	0.0671342	2.9145
D3UCLA1	0.0594446	2.88822
SERINC1	0.0575128	2.8635
LOC673501	0.0292459	2.83348
CYB5R1	0.0519013	2.83232
NPM1	0.0404045	2.78736
KLF9	0.014108	2.74291
MTHFD2	0.0100631	2.68264
LOC100047619	0.0503871	2.64645
CXADR	0.0178336	2.63263
ARL1	0.0416219	2.61082
DDIT4	0.055177	2.53846

**TES**	0.00207262	2.53139
**GNL3**	0.0338031	2.49353
**BAG1**	0.0762813	2.48734
**HSPA5**	0.0212666	2.42345
**MAGED1**	0.0748936	2.42273
**SURF4**	0.0265892	2.42065
**LOC632230**	0.0630886	2.41706
**ATF5**	0.0627761	2.36497
**TMEM66**	0.051648	2.35549
**EMP2**	0.0753342	2.33577
**GTPBP2**	0.0792519	2.30018
**RAB2A**	0.0255304	2.23765
**ASNS**	0.0257098	2.16522
**FRMD6**	0.000852198	2.15957
**ARRDC3**	0.00222955	2.15749
**PPID**	0.0226155	2.14159
**PSMC2**	0.0327666	2.13677
**4930564D15RIK**	0.0176103	2.136
**AARS**	0.00731448	2.0994
**LOC100047184**	0.0338157	2.06222
**LEPROTL1**	0.0388277	2.03284
**DAG1**	0.0657733	2.0142
**UNC5B**	0.020868	1.99822
**2310016C08RIK**	0.0390916	1.93367
**CAR9**	0.0668378	1.9326
**PHF10**	0.0307945	1.92007
**ZFP131**	0.00138053	1.90832
**AMOTL1**	0.0140476	1.90574
**MATR3**	0.0662897	1.90277
**EIF4A1**	0.0630459	1.89525
**XBP1**	0.0517098	1.845
**ANGPTL6**	0.067795	1.83673
**CLIC4**	0.0230876	1.82725
**DNAJB1**	0.0153923	1.82499
**ATP6AP1**	0.0628326	1.82357
**LSM14A**	0.0369352	1.81944
**PHF17**	0.0250547	1.81786
**5430434G16RIK**	0.0285426	1.80986
**PDAP1**	0.00136208	1.79807
**PTP4A2**	0.0256614	1.79342
**5830407P18RIK**	0.0696165	1.7851
**LARS**	0.0358683	1.78468
**APP**	0.00752626	1.77956
**MAP3K7IP2**	0.0134131	1.77925
**MAPRE1**	0.0817428	1.7565
**SGPP1**	0.0170613	1.73542
**EIF1A**	0.0198072	1.73532
**C230075M21RIK**	0.0527679	1.72364
**KLF7**	0.065102	1.71812
**TDE2**	0.00853208	1.69764
**ACBD3**	0.0818107	1.69487
**NPC1**	0.046168	1.6948
**ARPC2**	0.0449064	1.68939
**CHD7**	0.0419693	1.68736
**B230350I06RIK**	0.00278371	1.6814
**ATXN1**	0.0459388	1.67093
**TMEM11**	0.0570433	1.6698
**SEPT2**	0.0282158	1.66913

**EIF5**	0.0747865	1.65601
**HDLBP**	0.0384094	1.63695
**RHBDD1**	0.0263829	1.62166
**CDR2**	0.00503554	1.60396
**SEC11C**	0.0747305	1.60273
**SFRS1**	0.0118548	1.59675
**PDLIM7**	0.0662166	1.57859
**PLA2G12A**	0.0307543	1.57598
**SRR**	0.0250287	1.57565
**YWHAZ**	0.00296551	1.57409
**SCL0002975.1_346**	0.0323048	1.56854
**GDF15**	0.0175754	1.55388
**NFE2L1**	0.0380368	1.54195
**LOC100044779**	0.0429152	1.53997
**SLC39A6**	0.0514506	1.53002
**PALLD**	0.0155917	1.52999
**ZBTB7A**	0.0161306	1.52203
**PIGA**	0.0424155	1.51847
**2310004N11RIK**	0.0198673	1.5155
**LMAN1**	0.0808293	1.49971
**TBC1D20**	0.0331891	1.49174
**SERTAD2**	0.0167245	1.48949
**LOC385825**	0.00967628	1.48925
**LOC100040573**	0.00297983	1.48685
**TRIM39**	0.00136037	1.48316
**GOT2**	0.0788918	1.48068
**B230363H02RIK**	0.0197226	1.48016
**NOL5**	0.00090104	1.47976
**TRIB3**	0.00509689	1.46915
**ATP6V1A**	0.0156408	1.46387
**UBE2L3**	0.00718441	1.46192
**4833414E09RIK**	0.0558558	1.45836
**DDX3X**	0.0148139	1.45768
**LOC386218**	0.0801434	1.45513
**2600005C20RIK**	0.00524429	1.45263
**CCT4**	0.0511784	1.45207
**ZFC3H1**	0.00199781	1.45182
**LOC100041388**	0.0332207	1.44971
**TRIO**	0.0405929	1.43583
**PJA2**	0.0372816	1.43147
**FNDC3B**	0.0206864	1.42638
**AI450540**	0.0112198	1.42455
**GHITM**	0.0338714	1.42416
**SLAIN2**	0.0222799	1.42162
**ZFP292**	0.0634885	1.4214
**ACTR3**	0.0246466	1.41916
**3300001P08RIK**	0.0702191	1.4114
**AHCYL1**	0.0136768	1.40613
**RNF11**	0.00483154	1.40033
**BRD2**	0.00235381	1.398
**RNF103**	0.0798836	1.39745
**GORASP2**	0.035533	1.39319
**AMPD3**	0.0430843	1.38945
**RHBDL2**	0.070058	1.38845
**SLC30A5**	0.0363352	1.38714
**MAT2A**	0.0186903	1.3806
**LOC628101**	0.0706715	1.37652
**A830055I09RIK**	0.0236358	1.37426

**NDEL1**	0.00655472	1.37324
**LOC386005**	0.0112833	1.37321
**DNAJC5**	0.0377733	1.37006
**B930008G03RIK**	0.0400428	1.3696
**PHXR4**	0.0385397	1.36858
**RAP1B**	0.0760382	1.36687
**C330049H01RIK**	0.028841	1.3646
**HYOU1**	0.0296663	1.3614
**DAZAP2**	0.0638268	1.361
**SEC63**	0.0810866	1.36087
**CTAGE5**	0.0126316	1.36076
**4933407C03RIK**	0.014583	1.35353
**XPOT**	0.0574847	1.35342
**AARD**	0.0385003	1.35306
**LOC386021**	0.0361196	1.35305
**B930090K24RIK**	0.00992503	1.3529
**CDKN1A**	0.0451696	1.35016
**BC004022**	0.02455	1.34862
**BTAF1**	0.0145028	1.34695
**A530089A20RIK**	0.0129494	1.34634
**IMP3**	0.0667546	1.33763
**2810003C17RIK**	0.0299259	1.33563
**WASL**	0.0390466	1.33314
**LOC382157**	0.0809253	1.33162
**LOC632684**	0.0142012	1.33152
**CCT8**	0.00148574	1.32523
**BC003940**	0.0338723	1.3243
**RNF145**	0.0564885	1.32331
**TMEM68**	0.0304339	1.32297
**SLC25A33**	0.0183183	1.31955
**TIMM8A1**	0.0359779	1.31748
**RASA1**	0.0437328	1.31729
**LOC100045882**	0.00166876	1.31444
**RBM13**	0.0726117	1.31037
**SLC4A7**	0.0267135	1.30995
**BC030476**	0.0480239	1.30921
**MCL1**	0.00350473	1.30289
**STARD5**	0.0564875	1.30258
**LOC386078**	0.0240107	1.30182
**MRPL50**	0.0196983	1.29935
**CALU**	0.0662393	1.29437
**SUPT5H**	0.024837	1.29063
**E030027H19RIK**	0.0560051	1.28855
**FCHO2**	0.0821821	1.28372
**UBE2O**	0.0110508	1.27679
**C530050E15RIK**	0.00414077	1.27367
**ZFP263**	0.0661815	1.27175
**NAP1L4**	0.00343848	1.27164
**SEC24D**	0.0176635	1.27019
**TFG**	0.0743444	1.26857
**FAM171B**	0.016221	1.26728
**SCL0004190.1_3**	0.0815645	1.26688
**5330401F18RIK**	0.00885043	1.26512
**CCRN4L**	0.0314148	1.2631
**GNA13**	0.0681361	1.25904
**C230070D10RIK**	0.0228396	1.25894
**2700092H06RIK**	0.0132383	1.25804
**EIF4A2**	0.0440495	1.25632

**2810403A07RIK**	0.0792377	1.25478
**LOC385068**	0.0347998	1.25456
**M6PR**	0.0411173	1.25438
**C730026J16**	0.0254506	1.25402
**IQGAP1**	0.0381867	1.25247
**TRUB2**	0.0268411	1.25179
**LOC622404**	0.0555303	1.24914
**SLC38A2**	0.0617957	1.24883
**2010009J12RIK**	0.0321995	1.24539
**LOC639931**	0.0681379	1.24512
**SMEK2**	0.0355666	1.24503
**SLC39A7**	0.0693955	1.24047
**LOC100041797**	0.0726652	1.23998
**GALNT2**	0.0618391	1.23923
**CCNT1**	0.0835955	1.23839
**RBM9**	0.0113845	1.23562
**IRF2**	0.0252141	1.23533
**0610007L01RIK**	0.027303	1.23233
**ZFP639**	0.0797507	1.23219
**SPCS3**	0.0763736	1.23184
**PNRC2**	0.0708981	1.2299
**STRAP**	0.0779072	1.22911
**KCTD4**	0.0611754	1.22871
**WDR75**	0.0408621	1.22818
**PTBP1**	0.0311989	1.22808
**BC031781**	0.0164027	1.228
**AHCTF1**	0.015383	1.22675
**NRD1**	0.0514927	1.22529
**6720458F09RIK**	0.0468486	1.22383
**TSHZ1**	0.069827	1.22379
**RCOR1**	0.041274	1.22339
**PIP5K1A**	0.0186022	1.22155
**GPT2**	0.0748073	1.22075
**THYN1**	0.0655867	1.22057
**TFRC**	0.0532706	1.21564
**D130077B20RIK**	0.0109502	1.21515
**KRT15**	0.0585891	1.21381
**AHI1**	0.00183642	1.21276
**HNRPM**	0.0637759	1.21228
**D2ERTD391E**	0.0240117	1.20955
**1500001L15RIK**	0.0639766	1.20918
**NKTR**	0.0140592	1.20829
**2810426P10RIK**	0.0827519	1.20746
**ELAVL1**	0.0172692	1.20464
**2310007O11RIK**	0.0460993	1.20272
**C630030A18RIK**	0.0199023	1.20201
**SILG111**	0.0372419	1.20169
**5330403J18RIK**	0.0298877	1.20167
**EIF2S2**	0.00033657	1.20002
**5031436O03RIK**	0.0183185	1.19835
**ZC3H18**	0.00157172	1.19821
**CCND2**	0.0644247	1.19487
**SIAH1A**	0.0120281	1.19486
**DDX1**	0.0311899	1.19378
**WFS1**	0.0802049	1.19072
**RRP1B**	0.000245274	1.18898
**5033413D16RIK**	0.0418341	1.18759
**LOC216443**	0.0248742	1.18638

**2810407C02RIK**	0.0450203	1.18534
**RBMS1**	0.0840315	1.18378
**G3BP2**	0.0301857	1.18185
**GAPVD1**	0.00752808	1.18162
**AI314180**	0.0293066	1.18042
**RAB1**	0.00496573	1.17871
**PTK2**	0.00826469	1.17834
**PRDM2**	0.00218927	1.17676
**PPP3CA**	0.0622103	1.17667
**E130318A13RIK**	0.0418311	1.17635
**VPS54**	0.0486565	1.1739
**4930461P20RIK**	0.0349111	1.17198
**LOC100044376**	0.00341812	1.17196
**BRD3**	0.0747018	1.17169
**CCNL1**	0.0631192	1.17048
**ISCA1**	0.011329	1.17023
**2310051N18RIK**	0.0199717	1.16994
**SERBP1**	0.0747354	1.16993
**ZFP148**	0.00421232	1.16921
**CBFB**	0.0423377	1.16825
**NIPBL**	0.0775885	1.16788
**ANKHD1**	0.0535946	1.1668
**TUT1**	0.0412818	1.16578
**D0KIST3**	0.0368873	1.16573
**YTHDF1**	0.0067695	1.16571
**RAP2C**	0.020734	1.16486
**CRIM1**	0.0452301	1.16442
**ZRANB2**	0.076342	1.16164
**DHX15**	0.0265177	1.16135
**9630032J03RIK**	0.0505558	1.1606
**NPM3**	0.0697828	1.16042
**LOC386360**	0.0116986	1.15903
**ZXDA**	0.011716	1.15806
**USO1**	0.079429	1.15804
**EDNRB**	0.0783654	1.15758
**NFKBIZ**	0.00904545	1.15578
**PDPK1**	0.0167721	1.15258
**RSL1D1**	0.076662	1.15257
**D0HXS9928E**	0.0842822	1.15164
**ARHGAP17**	0.0294607	1.15102
**6720477C19RIK**	0.0229761	1.15086
**HTATSF1**	0.0272365	1.1508
**ARL8B**	0.0690676	1.1506
**OSR1**	0.0834256	1.15047
**5430417L22RIK**	0.068416	1.14985
**ARMCX1**	0.0772276	1.1495
**NOL14**	0.026541	1.14929
**LOC100046817**	0.0124591	1.14927
**DUSP4**	8.96E-05	1.14755
**IER3IP1**	0.0688004	1.14712
**5830406C17RIK**	0.00401435	1.14629
**EIF3A**	0.0218889	1.14609
**SCL0002315.1_12**	0.0177747	1.14443
**CHKA**	0.0650491	1.14417
**A730098D12RIK**	0.0779526	1.14376
**LOC100039607**	0.00241932	1.14318
**USP39**	0.0569984	1.14268
**2310037I24RIK**	0.0444974	1.14155

ERO1LB	0.077709	1.14148
9130011J15RIK	0.0189891	1.14009
1110001P04RIK	0.0453299	1.13963
C80913	0.0397766	1.13957
LHFPL2	0.00565402	1.13951
RARS	0.0517262	1.13944
TAF15	0.0183504	1.13828
ARF2	0.0294974	1.13793
HS3ST3B1	0.0147083	1.13789
HSPB6	0.063335	1.13767
IGF1R	0.0342507	1.13723
ZWINT	0.0178771	1.13716
DDX26	0.0565768	1.13707
TCOF1	0.000350485	1.13674
SYNCRIP	0.00465206	1.13632
SRA1	0.0274233	1.13592
EXOSC1	0.0742061	1.13542
2610101N10RIK	0.0696579	1.13475
LRRC59	0.0382503	1.13446
MED26	0.0680082	1.13396
9430008C03RIK	0.0582019	1.1334
ODZ2	0.0347469	1.13332
HSPD1	0.0452864	1.13326
5730494N06RIK	0.0449191	1.13323
C730026O12RIK	0.0175504	1.13283
CEBPG	0.0496107	1.13272
FASTKD5	0.0758444	1.13185
A630082K20RIK	0.0357983	1.13167
ANP32E	0.0325609	1.13074
UCK2	0.0531995	1.13025
ENSMUSG00000074075	0.0201671	1.12988
AI115600	0.0277822	1.12979
2610020C11RIK	0.000704179	1.12815
LUC7L	0.0288471	1.12813
TCERG1	0.0213594	1.12789
TMEM63B	0.0426256	1.12717
TMEM97	0.0820599	1.12695
2510012J08RIK	0.0357656	1.12599
C330006D17RIK	0.0100217	1.12585
TSSC8	0.000886579	1.12526
5730405I09RIK	0.0188509	1.12477
CCDC86	0.0377796	1.1245
A730021C13RIK	0.00382688	1.12422
RUVBL2	0.0712747	1.12405
C530036F05RIK	0.0196028	1.1235
PATL1	0.0163141	1.12188
ASCC2	0.0486624	1.12146
RABGEF1	0.0801622	1.12093
LOC100043821	0.0546252	1.12046
DAD1	0.0594016	1.12043
1110029L17RIK	0.0522243	1.12016
NAPB	0.00310464	1.11972
A630056H20RIK	0.00647165	1.1182
ZDHHC21	0.014315	1.11806
B230387C07RIK	0.065464	1.11801
HUWE1	0.00631252	1.11755
YTHDF2	0.0654789	1.11729
HS3ST3A1	0.0702617	1.11542

**JMJD3**	0.0721425	1.11507
**B230386D16RIK**	0.0425729	1.11471
**EPHB1**	0.0668196	1.11469
**D17WSU104E**	0.0707713	1.11332
**6030458P06RIK**	0.00714127	1.11265
**6430540M20RIK**	0.0111407	1.11225
**EPRS**	0.0786996	1.11167
**LOC100044172**	0.0372759	1.11084
**C230043G09RIK**	0.078572	1.11078
**AFTPH**	0.0523372	1.11065
**DDX26B**	0.0623933	1.1106
**EIF3S8**	0.0705829	1.11022
**ANKRD11**	0.0123771	1.10995
**E330027P06RIK**	0.0549151	1.1091
**UBE2E2**	0.0120468	1.10895
**TCP1**	0.0375911	1.10852
**PGRMC2**	0.00119574	1.10808
**CREB5**	0.0396605	1.10759
**C330023M02RIK**	0.043827	1.10732
**DUSP11**	0.0514681	1.10725
**HSD3B2**	0.0775332	1.10725
**2010007K12RIK**	0.0211633	1.1072
**SPG20**	0.000575066	1.10685
**B930011A14RIK**	0.0550849	1.10608
**TESK1**	0.0707215	1.10562
**RAB33B**	0.0340133	1.10519
**ABI2**	0.0079958	1.10445
**2610528B01RIK**	0.0253706	1.10396
**HECTD1**	0.00818801	1.10326
**A930004J17RIK**	0.0229614	1.10288
**SARS**	0.0487723	1.10286
**9330180L10RIK**	0.0632532	1.10277
**B230208H17RIK**	0.000493433	1.10221
**PUM1**	0.0500008	1.10197
**UBE4B**	0.0692614	1.10179
**CSNK2A1-RS3**	0.0470877	1.10164
**NHLRC2**	0.0171242	1.10163
**MSH6**	0.0615565	1.10093
**ATG2B**	0.0296601	1.1009
**R3HDM1**	0.0496784	1.10087
**2410002F23RIK**	0.063688	1.10038
**IPO5**	0.014224	1.10025
**B230213E18RIK**	0.0757012	1.10015
**FOSL2**	0.0744274	1.10006
**GOLGA3**	0.0311349	1.0995
**PRKRIR**	0.0656386	1.09945
**CHUK**	0.0107028	1.09882
**CSDA**	0.00489669	1.09855
**LRRC28**	0.0111325	1.09767
**UGP2**	0.0154432	1.09734
**SULT1A1**	0.0807816	1.09699
**STT3A**	0.0390678	1.09677
**MFAP1B**	0.0630782	1.0963
**E430033B07RIK**	0.0446631	1.09532
**BBX**	0.0175432	1.09498
**MAST2**	0.06025	1.09484
**SCL0004020.1_31**	0.0148467	1.09471
**SEC31A**	0.073395	1.09456

**ADAM9**	0.00513106	1.09436
**SSFA2**	0.041822	1.09428
**ARL5B**	0.0667491	1.09393
**CLDND1**	0.0108185	1.09373
**FAM129A**	0.0348774	1.09366
**HMGB2L1**	0.00937019	1.09349
**RIOK1**	0.0140593	1.09335
**LOC640972**	0.0832942	1.09283
**LOC100046891**	0.0812128	1.09249
**4933432B13RIK**	0.0117666	1.09242
**TAF13**	0.000345728	1.09228
**SAR1A**	0.0236314	1.09222
**VGLL4**	0.0278014	1.09182
**6430562A12RIK**	0.00578776	1.09131
**EIF4EBP2**	0.0277484	1.09105
**GRPEL1**	0.0701818	1.09039
**PA2G4**	0.0125248	1.09005
**C730046C01RIK**	0.012863	1.08987
**LOC100046483**	0.0207642	1.08967
**TMEM93**	0.0306805	1.08955
**ANKRD49**	0.0473284	1.08927
**2410016O06RIK**	0.00832156	1.08904
**LOC100041567**	0.000224179	1.08902
**2310079P03RIK**	0.0584297	1.0882
**A530082L16RIK**	0.0103266	1.08792
**UBQLN1**	0.0471097	1.08737
**TXNDC12**	0.0595766	1.0868
**SFRS4**	0.0433524	1.0865
**GATAD2B**	0.0554915	1.0863
**SCL0002785.1_49**	0.0471539	1.08518
**LOC100046841**	0.0590711	1.08487
**PLAA**	0.0225664	1.08363
**E030040G24RIK**	0.0459056	1.0833
**MAPK6**	0.0790551	1.08315
**A130039H09RIK**	0.0285578	1.08292
**LIMS2**	0.0615164	1.08274
**ARPC5L**	0.0765401	1.08235
**A730094H17RIK**	0.0450112	1.08225
**A430096A09RIK**	0.00350776	1.08119
**NMU**	0.0632197	1.08118
**CITED2**	0.0364196	1.08114
**GBE1**	0.0319895	1.08042
**1810055E12RIK**	0.0441545	1.08042
**SHC1**	0.0666061	1.08021
**MTERFD1**	0.0527988	1.08008
**LOC545056**	0.0132827	1.07997
**SACM1L**	0.0335637	1.07991
**PEX11A**	0.0148735	1.07977
**STXBP3**	0.0756435	1.07938
**BIRC2**	0.0217221	1.07915
**CASC3**	0.00822219	1.07901
**BIRC1CL**	0.0814493	1.0788
**CDC2L5**	0.0299354	1.07863
**PAPPA**	0.0711519	1.07862
**9630021O20RIK**	0.0583959	1.07792
**9130005N14RIK**	0.0336476	1.07789
**TNS1**	0.00669315	1.07743
**HPBRII4**	0.0632245	1.07742

**NDUFA10**	0.0676991	1.07718
**WIPF2**	0.00223778	1.07711
**DCUN1D5**	0.000731884	1.07705
**ATG16L1**	0.0580533	1.07681
**TLK2**	0.0576693	1.07576
**ARMCX3**	0.0327027	1.07515
**NAMPT**	0.0413757	1.07497
**HAX1**	0.0734338	1.07483
**A130047F11RIK**	0.0588331	1.07463
**OSBP**	0.061821	1.07443
**HYAL1**	0.0495127	1.07442
**9530062M13RIK**	0.0511659	1.07433
**BAG2**	0.0215762	1.07421
**LUC7L2**	0.076081	1.07381
**NCRNA00117**	0.0713186	1.07371
**PARD6A**	0.0519627	1.0735
**FIN14**	0.0222183	1.07332
**MTAP1B**	0.000913543	1.07327
**LOC100047794**	0.0668872	1.07276
**DTNB**	0.0316582	1.07205
**RRP12**	0.0291556	1.07188
**GDF10**	0.0306525	1.07169
**1110007M04RIK**	0.0611767	1.0715
**LOC100047323**	0.0543415	1.07135
**BCAT2**	0.0442628	1.07116
**LOC381739**	0.0431577	1.07048
**9130422G05RIK**	0.0336779	1.07006
**BAG5**	0.0239648	1.06932
**6530401O14RIK**	0.0256452	1.06865
**2400010D15RIK**	0.0284985	1.06861
**0610009J05RIK**	0.0787182	1.06819
**CNOT4**	0.033067	1.068
**LOC100048645**	0.0121439	1.06782
**LOC386246**	0.0559469	1.06756
**DDX27**	0.083415	1.06754
**RRAS2**	0.00401464	1.06697
**A230013K13RIK**	0.0588476	1.06644
**IMPAD1**	0.0410716	1.06632
**ECEL1**	0.0550143	1.06622
**EG433182**	0.0270497	1.06614
**EP300**	0.0646713	1.06583
**LOC100044566**	0.0292019	1.06561
**PREP**	0.0793014	1.06558
**RAD23B**	0.00797853	1.06512
**9130020K20RIK**	0.0442333	1.06511
**LOC100047419**	0.0492304	1.06462
**SLC25A39**	0.0319843	1.06453
**D330001F19RIK**	0.00883507	1.06437
**TOR1B**	0.0261033	1.06422
**IL6ST**	0.0525715	1.06369
**AUTS2**	0.00161096	1.0634
**D230047F17RIK**	0.021436	1.06339
**AI847670**	0.0554138	1.06326
**A130004B21RIK**	0.028701	1.06324
**2810008M24RIK**	0.00928179	1.06291
**GOLGB1**	0.045854	1.06263
**IDE**	0.0627551	1.06249
**COG8**	0.073108	1.06246

**LOC100046343**	0.0127738	1.06243
**APH1B**	0.0262149	1.06217
**C230055K21RIK**	0.00449773	1.06205
**9530005P22RIK**	0.0718825	1.0618
**NIBAN**	0.0802005	1.06174
**COBLL1**	0.0668264	1.06157
**FAM168B**	0.0466494	1.06152
**ADORA2B**	0.0206988	1.06098
**KIF3A**	0.067353	1.06088
**9630005B22RIK**	0.0134901	1.06071
**CDH23**	0.0101631	1.06068
**6430510M02RIK**	0.0455131	1.06064
**PURB**	0.0279173	1.06061
**LOC671878**	0.0122314	1.06028
**SNUPN**	0.0749016	1.05986
**LEO1**	0.0505432	1.05982
**B130008O17RIK**	0.0415895	1.0595
**PAPOLA**	0.00101661	1.05934
**USP33**	0.0446985	1.05919
**CREB3L2**	0.0834707	1.05916
**E330031M19RIK**	0.0699788	1.05888
**ZFYVE20**	0.0401408	1.05877
**9030425E11RIK**	0.0416945	1.05869
**LIN54**	0.0566464	1.05848
**XAB1**	0.0598041	1.05838
**TXNDC13**	0.0319223	1.05765
**WHSC2**	0.0392252	1.05741
**D230046F09RIK**	0.0334314	1.05736
**TRNT1**	0.0274379	1.05701
**ADAMTS18**	0.0474681	1.0567
**TGFBR1**	0.00888551	1.05651
**4921506J03RIK**	0.0144873	1.05613
**HIGD1A**	0.0150046	1.056
**TTC14**	0.000316498	1.05591
**SFRS11**	0.0844056	1.05542
**ZC3H6**	0.0156883	1.05531
**PK3**	0.0805574	1.05529
**BAT2D**	0.0845103	1.05524
**TNPO3**	0.00544093	1.05498
**4921513D23RIK**	0.0627338	1.05493
**ZMIZ1**	0.038093	1.05483
**CHD1**	0.0249425	1.05466
**NOL8**	0.0216216	1.05456
**VAMP2**	0.0447594	1.05436
**MLL3**	0.0192111	1.05431
**FOXJ3**	0.00516726	1.05422
**LOC383077**	0.00816754	1.05408
**DDX50**	0.060691	1.05387
**A930007B11RIK**	0.00593028	1.05361
**GMPPB**	0.0758665	1.05352
**SLTM**	0.0363391	1.05342
**OXR1**	0.0318853	1.05327
**KCNN1**	0.0734885	1.05323
**MIST1**	0.0575795	1.05313
**B3GALNT2**	0.072001	1.05306
**SRPRB**	0.0409259	1.05304
**9330177P18RIK**	0.0558781	1.05296
**NCOR1**	0.0267168	1.05278

ARL10C	0.0358503	1.0527
CREBBP	0.0606916	1.05265
NXF1	0.0447015	1.05252
LOC386144	0.0552565	1.05211
C130067A03RIK	0.0483025	1.05177
A430023D23RIK	0.00896896	1.0517
6430548O12RIK	0.0636226	1.0515
4931403I22RIK	0.00562659	1.05142
A330084H10RIK	0.00135936	1.05137
TMEM57	0.0288484	1.05125
CDC14B	0.055966	1.05088
RAB40C	0.0568365	1.05077
A530025E09RIK	0.0227938	1.05071
ZFP180	0.00726798	1.05055
GOLGA4	0.0341208	1.05054
2610319H10RIK	0.0750431	1.05047
BHLHB8	0.0773172	1.05043
1110001C20RIK	0.0554894	1.05019
CHK	0.017706	1.05016
USP16	0.0543123	1.04964
RAB5B	0.00669928	1.04953
XPO6	0.0399919	1.0494
TPD52L2	0.000788565	1.04934
DIS3	0.0435882	1.04905
A730086L23RIK	0.000937123	1.04903
UQCRB	0.0490721	1.04898
4833444G19RIK	0.0285489	1.04895
BC016608	0.0641053	1.04894
2610207I05RIK	0.00538366	1.04892
ARFGAP3	0.0222861	1.04887
ZRANB3	0.0737259	1.04828
PIAS1	0.0393118	1.0482
CWF19L2	0.00351195	1.04798
LOC219106	0.0371972	1.04795
XPNPEP3	0.0435422	1.0478
STK40	0.0313008	1.04779
E030003O11RIK	0.00877331	1.0477
SWAP70	0.0704615	1.04758
5830431A10RIK	0.0337864	1.0475
PLEKHA3	0.012946	1.04745
RWDD4A	0.00811141	1.04738
AMMECR1L	0.0689945	1.04706
ARCN1	0.067398	1.04694
D130060C09RIK	0.0148913	1.04685
C030046E11RIK	0.0616207	1.04673
A930019L04RIK	0.00181864	1.04642
C130040J09RIK	0.0247894	1.04636
LOC100047762	0.0386644	1.04632
ZFX	0.0216443	1.04625
D030069G17RIK	0.0389515	1.04614
SEMA3C	0.033918	1.04593
TARS	0.0144493	1.04587
GIGYF1	0.059949	1.04582
STARD3NL	0.0269799	1.04581
1110007L15RIK	0.0527111	1.04579
GTF2E1	0.0587876	1.04578
NRF1	0.0323685	1.04564
USP36	0.000324605	1.04558

**SOS2**	0.0709155	1.04556
**5430440L12RIK**	0.0451831	1.0454
**A530024C08RIK**	0.0513409	1.04529
**DNAJC1**	0.044571	1.04518
**NAT12**	0.0621189	1.04517
**SBF1**	0.0334752	1.04497
**HEG1**	0.024587	1.04492
**ERN1**	0.05001	1.04464
**PRKX**	0.0496579	1.04457
**LOC382128**	0.0566627	1.04413
**IBTK**	0.0718751	1.04343
**UBE2D3**	0.0768009	1.04338
**LOC233771**	0.047641	1.04332
**ZBTB38**	0.00591782	1.04275
**ZFP143**	0.053251	1.0425
**A430087B05RIK**	0.0348918	1.04248
**FARSA**	0.0357447	1.0423
**ZFP318**	0.0407883	1.0422
**D630041L13RIK**	0.0699446	1.0422
**FOXP1**	0.0065887	1.04187
**VMN2R-PS14**	0.0530873	1.04185
**PCYT1A**	0.0154448	1.04169
**B430305P08RIK**	0.0013163	1.04143
**LTV1**	0.0532955	1.04063
**ZUBR1**	0.0133611	1.0406
**LOC386270**	0.00299238	1.04052
**H13**	0.0342683	1.04043
**C87860**	0.0375579	1.04042
**VHL**	0.0786351	1.04018
**ZFP655**	0.0579708	1.03994
**TMEM23**	0.0155357	1.03992
**CSNK1G3**	0.000183637	1.03991
**BRPF1**	0.0725568	1.03989
**POGK**	0.0346007	1.03976
**D630040I23RIK**	0.0478692	1.03971
**STK39**	0.0567904	1.03971
**BC038156**	0.0168926	1.03966
**A230067E15RIK**	0.0560517	1.03949
**FERT2**	0.0656079	1.03945
**KCMF1**	0.0719232	1.03943
**GNL2**	0.00931313	1.03939
**9630015D15RIK**	0.052709	1.03933
**MAP2K1**	0.0497732	1.03927
**4832404P21RIK**	0.00313548	1.03921
**D430007A19RIK**	0.0190039	1.03918
**AFF4**	0.0445214	1.03909
**E130016E03RIK**	0.0306483	1.03908
**1600021P15RIK**	0.000885614	1.03907
**1700109H08RIK**	0.0049231	1.03905
**5730455O13RIK**	0.0772817	1.03887
**4921505C17RIK**	0.00343175	1.03883
**N6AMT1**	0.0440464	1.03881
**TXNRD3**	0.0307985	1.03867
**A730004F22RIK**	0.00489134	1.03864
**B930046C15RIK**	0.0449461	1.03861
**ZSWIM1**	0.0318779	1.03853
**MYST4**	0.071996	1.03829
**9830169C09RIK**	0.0650264	1.03828

**SEC61A**	0.0351235	1.0379
**D030034I04RIK**	0.0451531	1.03788
**D330011G23RIK**	0.0110172	1.03787
**CPEB2**	0.0103482	1.03776
**MORC3**	0.00248511	1.03763
**RALGPS1**	0.0834656	1.0376
**ZMAT3**	0.0409881	1.03753
**EHBP1L1**	0.0387732	1.03739
**CSE1L**	0.00181102	1.03737
**MAPK1IP1**	0.0099415	1.03733
**E130216C05RIK**	0.0452743	1.03722
**2310045K21RIK**	0.0210109	1.03717
**A330017A19RIK**	0.0226348	1.03696
**B230345P09RIK**	0.0768838	1.03684
**IL6RA**	0.0376357	1.03674
**MASP2**	0.0464832	1.03651
**2010203O07RIK**	0.058147	1.03651
**SRFBP1**	0.0311329	1.0365
**RAI1**	0.0127066	1.03646
**C230009C22RIK**	0.0194253	1.03636
**LOC240672**	0.0827984	1.03634
**C230064E07RIK**	0.0502024	1.0363
**6030458C11RIK**	0.00223868	1.03629
**MARK3**	0.0554764	1.03624
**FBXO42**	0.0172887	1.03621
**3632431M01RIK**	0.00223541	1.03613
**9330171D12RIK**	0.0433417	1.03604
**SLC25A16**	0.0117659	1.03595
**ERGIC2**	0.0663396	1.03586
**5530401J07RIK**	0.034997	1.0358
**MOSPD1**	0.081935	1.03568
**NARG1L**	0.00182543	1.03566
**E030040L08RIK**	0.0409582	1.03558
**MADH1**	0.0775623	1.03558
**RBM26**	0.014438	1.03556
**A830006J06RIK**	0.000786775	1.03537
**IKZF5**	0.0486748	1.03532
**RPP38**	0.0485561	1.03531
**ALKBH**	0.0658259	1.03521
**AI551093**	0.0359483	1.0352
**1810073N04RIK**	0.0533558	1.0352
**EIF4ENIF1**	0.0823625	1.03508
**ING2**	0.0185592	1.03499
**5730564L20RIK**	0.0270008	1.03499
**2810405J23RIK**	0.036068	1.03499
**DDX3Y**	0.0444172	1.03498
**PRMT5**	0.0129732	1.03495
**THSD4**	0.0446834	1.03481
**DYSF**	0.00258272	1.03472
**SKI**	0.0351318	1.03442
**D230020C06RIK**	0.046518	1.03439
**D130059P03RIK**	0.046096	1.0343
**8030474H12RIK**	0.0451872	1.03408
**USP1**	0.0374506	1.034
**4933417E08RIK**	0.0830951	1.03399
**EIF2C2**	0.0627086	1.03394
**NRIP2**	0.0566495	1.03391
**D430035B07RIK**	0.00876015	1.03387

**3230401L03RIK**	0.0726835	1.03371
**3110038B19RIK**	0.0259348	1.03357
**ZC3H13**	0.0356374	1.0333
**2310032M22RIK**	0.0345063	1.03329
**ZFP91-CNTF**	0.0231234	1.03327
**SLC31A1**	0.051535	1.03327
**9130215G10RIK**	0.0479315	1.03324
**MAGOH**	0.0350338	1.03312
**2210417D09RIK**	0.0128371	1.03308
**C030048B08RIK**	0.0174142	1.03287
**SMURF1**	0.0476468	1.03264
**LOC100045040**	0.0680264	1.03261
**RARRES1**	0.0328031	1.0326
**4432404P07RIK**	0.00297719	1.03257
**GPLD1**	0.0849321	1.03256
**CUL5**	0.0222698	1.03244
**4733401O04RIK**	0.0526338	1.03237
**WHSC1**	0.0298223	1.03219
**TAF1A**	0.0510136	1.03214
**LOC386199**	0.0560143	1.03213
**G0S2**	0.00969458	1.03204
**LOC624198**	0.0132218	1.03156
**6030419I20RIK**	0.0408862	1.03153
**ICK**	0.0496261	1.03148
**PRPF8**	0.0637097	1.03146
**RASD2**	0.0419723	1.03139
**OGFRL1**	0.0231435	1.03138
**FUBP1**	0.0306875	1.03133
**A530021E08RIK**	0.0459492	1.03131
**ADRBK2**	0.0658923	1.0313
**PUM2**	0.0284171	1.03125
**KANK1**	0.0133565	1.03119
**UBFD1**	0.0766715	1.0309
**E530004K11RIK**	0.0060592	1.03085
**NARG1**	0.0272428	1.03079
**9130416J18RIK**	0.0270334	1.03078
**ELAC1**	0.0284949	1.03063
**E130112J05RIK**	0.0187418	1.0304
**8430415N23RIK**	0.055509	1.03036
**GDAP10**	0.0216499	1.03034
**ATF2**	0.0561052	1.03033
**TRMT2A**	0.034538	1.03024
**NSUN2**	0.0615544	1.03022
**D7ERTD413E**	0.0255057	1.0301
**MAPK8**	0.0346151	1.03009
**4930402E16RIK**	0.0304574	1.02996
**B230380D07RIK**	0.0551239	1.0299
**NECAP1**	0.0429851	1.02988
**C230066H01RIK**	0.0120474	1.02983
**PIAS4**	0.0747519	1.02966
**SPRR1A**	0.046188	1.0295
**PDXDC1**	0.058757	1.02925
**D830050E09RIK**	0.0815636	1.02912
**C030034J23RIK**	0.0311329	1.02851
**VBP1**	0.0477297	1.02843
**UBR2**	0.0599329	1.02843
**C430045L21RIK**	0.0304945	1.02841
**RAB22A**	0.041827	1.0284

**RRN3**	0.0239163	1.02836
**6030434H13RIK**	0.0126445	1.02833
**4932441K18RIK**	0.00707956	1.02818
**CDK8**	0.0432287	1.02814
**5830418G11RIK**	0.0621709	1.02808
**CHERP**	0.0129765	1.02799
**COQ5**	0.0131071	1.02798
**ZFP770**	0.0660223	1.02793
**RAB7**	0.0284928	1.02789
**CD3EAP**	0.0393296	1.02777
**TRB**	0.00435373	1.02762
**ZFP91**	0.0288366	1.02759
**MARCH6**	0.0824744	1.02758
**WHDC1**	0.00551647	1.02755
**CYLD**	0.0724443	1.02752
**B130008D24RIK**	0.0466695	1.02749
**9630009A08RIK**	0.0278855	1.02707
**BDNF**	0.00171452	1.02705
**5730403B10RIK**	0.0197516	1.02687
**ZBTB39**	0.00488301	1.02684
**PLCXD1**	0.019326	1.02681
**SPAG9**	0.0391539	1.02664
**ANXA11**	0.074733	1.02651
**VPS18**	0.0111112	1.02649
**2610015J01RIK**	0.0328914	1.02648
**2410042D21RIK**	0.0439135	1.02648
**LOC381243**	0.0482481	1.02643
**5330435L01RIK**	0.0487007	1.0264
**1810007M14RIK**	0.030571	1.02639
**TASP1**	0.0274129	1.02637
**TOP3B**	0.0540368	1.02634
**PLEKHM1**	0.0237698	1.02632
**MOBK1B**	0.0726419	1.02622
**A630036P20RIK**	0.0138496	1.0261
**A430089I07RIK**	0.00494493	1.02602
**GALNT11**	0.0344969	1.026
**PTRH2**	0.0309781	1.02599
**NPHP3**	0.0338131	1.02593
**A430083I17RIK**	0.0790942	1.02579
**KITL**	0.0210403	1.02578
**LOC100048020**	0.027238	1.02577
**FBXO30**	0.0122246	1.02575
**4732471D19RIK**	0.0367326	1.02566
**NPTX2**	0.0183101	1.02556
**TPP2**	0.0258527	1.02553
**2700024H10RIK**	0.0362638	1.02545
**ZFP322A**	0.0355278	1.0254
**9030605E16RIK**	0.000389755	1.02536
**ANXA7**	0.0389979	1.02532
**A130020K16RIK**	0.00434037	1.0253
**NMD3**	0.0579215	1.0253
**CDKL5**	0.0157448	1.02528
**GMPS**	0.0688657	1.02503
**9530062G19RIK**	0.0390291	1.02498
**ZFP317**	0.0781367	1.02492
**D530007E13RIK**	0.0599098	1.02485
**HOOK3**	0.0100123	1.02475
**LOC385959**	0.0772862	1.02463

**AKAP8**	0.00883582	1.02462
**A130053G17RIK**	0.0109287	1.02456
**C330006P03RIK**	0.0145598	1.0245
**RC3H2**	0.0507115	1.0245
**4631409F12RIK**	0.00718045	1.02447
**LOC385019**	0.0408043	1.02442
**TBRG4**	0.0633097	1.02441
**SCFD1**	0.0455507	1.02425
**RBBP4**	0.0177325	1.02411
**ACOT5**	0.0522969	1.02404
**ZFP445**	0.0372643	1.02402
**TRIM56**	0.0257979	1.02388
**D930014N22RIK**	0.0130101	1.02377
**7030401E22RIK**	0.0321246	1.02377
**B230312I18RIK**	0.0196652	1.02364
**PSG23**	0.0585064	1.02361
**C230091E20RIK**	0.0129345	1.0236
**6720406L13RIK**	0.0317164	1.02359
**3110023B02RIK**	0.0545185	1.02349
**C130072A16RIK**	0.030762	1.02334
**A330049H05RIK**	0.0206633	1.02309
**GPR23**	0.0210981	1.02304
**4930518I15RIK**	0.0609838	1.02281
**D630048P19RIK**	0.0429733	1.02278
**RNMT**	0.0788181	1.02278
**LOC547150**	0.0478574	1.02277
**AA408296**	0.0631656	1.02269
**ZBTB43**	0.0825729	1.0225
**USP42**	0.00844412	1.02249
**LLPH**	0.0303291	1.02244
**C630029M13RIK**	0.00229764	1.02238
**CEP57**	0.0472976	1.02238
**SH3GL1**	0.0643029	1.02233
**LOC386179**	0.0206896	1.02231
**LOC227393**	0.0812895	1.02228
**DDX46**	0.0336371	1.02226
**E4F1**	0.0496532	1.02223
**5930437C20RIK**	0.0812568	1.02201
**SUHW4**	0.0560656	1.02198
**GM1815**	0.0478444	1.02195
**ABCC1A**	0.0478841	1.02193
**C230081A13RIK**	0.0414853	1.02188
**LOC277385**	0.0607574	1.02181
**DICER1**	0.0457071	1.02173
**B130017I01RIK**	0.0733693	1.02172
**CUEDC1**	0.0423334	1.02165
**SSR1**	0.0165322	1.02164
**SKZ1-PENDING**	0.0115851	1.02159
**PKNOX1**	0.0137748	1.02159
**9930013M16RIK**	0.0246169	1.02158
**LOC100046963**	0.0813164	1.02154
**A630020I15RIK**	0.0389729	1.02147
**6330400D04**	0.0749409	1.02143
**1200003I07RIK**	0.0647951	1.02139
**CACNA2D1**	0.0732181	1.02134
**NARS2**	0.0406144	1.02128
**CTTNBP2**	0.00949906	1.02119
**LSM12**	0.0322379	1.02119

**BRAF**	0.00531224	1.02114
**AFG3L2**	0.0192853	1.02113
**E130309L16RIK**	0.0263174	1.02112
**PIK3CA**	0.0601536	1.02109
**RAPGEF5**	0.00695914	1.02105
**SNX27**	0.0810337	1.02097
**D430021P20RIK**	0.00216232	1.02091
**SNIP1**	0.0483343	1.02091
**D130004H04RIK**	0.0805811	1.02091
**5730470C09RIK**	0.021538	1.02085
**SENP1**	0.0428873	1.02075
**BC021438**	0.0239862	1.02069
**REV1**	0.00583549	1.02067
**OVGP1**	0.0235316	1.02064
**RASA2**	0.0385461	1.0206
**TM9SF2**	0.0471081	1.02054
**TRRP4AP-PENDING**	0.028588	1.02052
**ATPI**	0.0221453	1.02049
**IFRD2**	0.0302283	1.02047
**YIPF6**	0.0193652	1.02041
**TRPV4**	0.0611553	1.02041
**9530031H08RIK**	0.060483	1.02037
**CDC25A**	0.0308489	1.02031
**YTHDF3**	0.0559852	1.02001
**A930039H18RIK**	0.0496205	1.02
**METTL6**	0.0713244	1.01999
**A130019H11RIK**	0.0317517	1.01996
**E330020K23RIK**	0.0640867	1.01995
**ZFML**	0.0125969	1.01992
**D030040K20RIK**	0.0664586	1.0199
**SLC25A4**	0.0394147	1.01988
**B930037B01RIK**	0.0107999	1.01974
**PHRF1**	0.0573099	1.01968
**D430019K11RIK**	0.0354606	1.0196
**A230067G21RIK**	0.0198252	1.01958
**1500019C06RIK**	0.00571871	1.01954
**PTEN**	0.0578439	1.01945
**ABCF3**	0.00351509	1.01944
**TERF2IP**	0.03854	1.01936
**TNRC6B**	0.0450809	1.01928
**CCDC155**	0.0554619	1.01927
**2810455B08RIK**	0.0132637	1.01925
**D030070I18RIK**	0.0142466	1.01924
**B430109P06RIK**	0.00618526	1.01923
**LOC100041103**	0.0650487	1.01921
**A430090L17RIK**	0.0735139	1.01911
**ZFP106**	0.0275739	1.01904
**PRPF40A**	0.00787931	1.01895
**B9D2**	0.0140384	1.0189
**JMJD2C**	0.0171392	1.01888
**WARS2**	0.0143238	1.01875
**C430015M08RIK**	0.0153143	1.01874
**TFB1M**	0.0747347	1.01874
**TGS1**	0.0108762	1.01871
**3930402G23RIK**	0.0777469	1.01864
**A530030B07RIK**	0.0362753	1.01863
**ZFP516**	0.0666623	1.01863
**EG434077**	0.0785642	1.01862

**PI4K2B**	0.0104906	1.01854
**SLC30A4**	0.00353749	1.01852
**ZFP800**	0.00139589	1.01852
**PHF14**	0.0356219	1.01852
**PVRL3**	0.0561438	1.01852
**D930049D10RIK**	0.0116521	1.01848
**2010305A19RIK**	0.00135759	1.01846
**BC024659**	0.0389993	1.01836
**9330174H21RIK**	0.0325069	1.01831
**ABCE1**	0.0034702	1.01828
**B930097J01RIK**	0.0389593	1.01824
**NOL11**	0.0239269	1.01814
**PTPDC1**	0.0431353	1.0181
**D330018I10RIK**	0.0504566	1.01797
**4732418C07RIK**	0.0593117	1.01789
**9330158N06RIK**	0.0361994	1.01784
**IMPDH1**	0.057804	1.01782
**2900045G02RIK**	0.0834124	1.01782
**6030410I10RIK**	0.0300881	1.01776
**TEX10**	0.0614418	1.01776
**TMEM16B**	0.0605924	1.01773
**1700009P03RIK**	0.0188606	1.01769
**ENSA**	0.00884078	1.01764
**LOC100048123**	0.000415473	1.01758
**ZFP790**	0.00366899	1.01757
**ANK2**	0.0570894	1.01755
**2600011C06RIK**	0.0427368	1.01752
**NFRKB**	0.0586286	1.01752
**SPRR2F**	0.024852	1.01748
**PARN**	0.0437173	1.01741
**RNPC2**	0.024208	1.01738
**LOC384344**	0.0128529	1.01734
**C130073O12RIK**	0.0382293	1.01731
**A130076G11RIK**	0.047087	1.01731
**2010005J08RIK**	0.00807942	1.01726
**PLP1**	0.0375471	1.01726
**9130206N08RIK**	0.0136587	1.01713
**EEG1-PENDING**	0.00988614	1.01712
**DYNC1LI1**	0.0342973	1.01712
**WWP1**	0.052597	1.01708
**FADS3**	0.0298814	1.01707
**D230007L07RIK**	0.0770563	1.01705
**4732460K03RIK**	0.0378116	1.01704
**LOC100045679**	0.0309524	1.01689
**DHX8**	0.0507591	1.01685
**SMCX**	0.0107197	1.01683
**A530082C11RIK**	0.0366368	1.01682
**RIF1**	0.0220095	1.0168
**EXTL1**	0.0742154	1.01673
**LOC100043402**	0.0322624	1.01669
**HSPA4**	0.055064	1.01667
**5330438F16RIK**	0.0641931	1.01666
**PNPO**	0.0142715	1.01663
**9230112E08RIK**	0.0744083	1.01662
**C130090G16RIK**	0.0283366	1.0166
**RECQL4**	0.0585087	1.01655
**CNPY3**	0.0365435	1.01654
**ZFP553**	0.00747907	1.01653

**E130112N23RIK**	0.0489578	1.01653
**A130027D22RIK**	0.0497598	1.01647
**LOC234374**	0.0705556	1.01642
**UTP14A**	0.0192992	1.01639
**A430071O12RIK**	0.0612354	1.01639
**C130039O16RIK**	0.0253972	1.01636
**HMG20A**	0.036259	1.0163
**A630075K04RIK**	0.0337112	1.01627
**OTUB2**	0.0754048	1.01623
**2010007L18RIK**	0.0122847	1.01621
**E330019D12RIK**	0.0591092	1.01608
**LOC386081**	0.063856	1.01606
**LOC100046905**	0.0459095	1.01597
**OTUD3**	0.0358645	1.01593
**C920016N10RIK**	0.0318113	1.01589
**ARRB1**	0.0578322	1.01583
**SYCE2**	0.071342	1.01576
**ARFGAP1**	0.00441882	1.01564
**RCN3**	0.0730752	1.01563
**0610011I19RIK**	0.0685689	1.01562
**IFNGR2**	0.0338799	1.01555
**5730419I09RIK**	0.0821827	1.01555
**GNMT**	0.00381814	1.0154
**2610027L16RIK**	0.00044252	1.01522
**PPM1L**	0.0122024	1.01521
**4932441P04RIK**	0.0476648	1.0152
**CTH**	0.0509882	1.01509
**B430114K07RIK**	0.00593319	1.01504
**2610301F02RIK**	0.0510301	1.01499
**SDCCAG1**	0.0788404	1.01499
**B230337C21RIK**	0.0126379	1.01489
**B130044D17RIK**	0.0267252	1.01484
**DLD**	0.0708449	1.01484
**E530018O14RIK**	0.0621385	1.0148
**C630002L24RIK**	0.0343689	1.01478
**1700064E03RIK**	0.0140482	1.01476
**E330013M12RIK**	0.0397127	1.01471
**MED1**	0.0529711	1.0147
**9530018I07RIK**	0.0610576	1.0147
**C630011I23**	0.0308038	1.01468
**C430014M02RIK**	0.0240155	1.01465
**LOC100047009**	0.0341913	1.01461
**D930046H04RIK**	0.060246	1.01461
**ARF4**	0.0330002	1.01459
**RASSF4**	0.0252276	1.01449
**LOC381336**	0.034537	1.01445
**PRPF4**	0.000648491	1.01444
**GOPC**	0.0149719	1.01443
**4933411D12RIK**	0.0721192	1.0144
**6030439I24RIK**	0.0340264	1.01439
**9830118G07RIK**	0.0747277	1.01434
**GLT1D1**	0.0781029	1.01424
**RBBP6**	0.0272242	1.01421
**LOC626061**	0.0382177	1.01415
**BC031575**	0.0289957	1.0141
**6820437F20RIK**	0.0157764	1.01407
**USP48**	0.049561	1.01404
**FBXW2**	0.0240901	1.014

ADSS2	0.0614792	1.014
9630044E13RIK	0.0181928	1.0139
C230094A19RIK	0.0204333	1.0139
9430043O10RIK	0.031615	1.01389
9530071H01RIK	0.00527525	1.01385
CYP20A1	0.000628452	1.01384
LOC381747	0.0411877	1.01384
EPB4.1	0.0104852	1.01378
CPEB4	0.00327353	1.01373
PTPRZ1	0.0166678	1.0137
LOC668047	0.069967	1.0137
2210010L05RIK	0.0147756	1.01365
A130099L09RIK	0.0202122	1.01364
TTC23	0.0212627	1.01358
PCNX	0.0190994	1.01356
OCRL	0.0137636	1.01355
ALG11	0.00679904	1.01354
1810008K04RIK	0.00485612	1.0135
D930026K06RIK	0.0529946	1.01349
B130039D17RIK	0.00261973	1.01346
D430007J11RIK	0.0516341	1.01341
BIRC6	0.0438821	1.0134
E130202F10RIK	0.016001	1.01335
1700040A22RIK	0.0413734	1.01335
2310047C21RIK	0.0730347	1.01335
C030046K23RIK	0.065616	1.01334
BC010584	0.0449438	1.01322
5730543M03RIK	0.0165277	1.01319
SCL000168.1_25	0.0600956	1.01317
KPNA3	0.0694361	1.01314
9430068D24RIK	0.0351358	1.01313
RPN1	0.00444598	1.0131
5730410E15RIK	0.0608519	1.01308
A730082L10RIK	0.051055	1.01305
C130072C03RIK	0.019688	1.01303
IRGQ	0.0130312	1.01296
A630024K09RIK	0.0356007	1.01286
FOXN2	0.00203542	1.01284
SP3	0.0251412	1.01284
LOC636687	0.024621	1.01283
PCDH7	0.0186217	1.01273
RAN	0.0657602	1.01271
GABPB1	0.030674	1.01267
SNX7	0.0155193	1.01254
B130018P07RIK	0.00153927	1.01249
EG226654	0.0339463	1.01248
MGA	0.0108013	1.01239
NUDEL-PENDING	0.021905	1.01234
D330016G23RIK	0.0322443	1.01229
GTL7	0.0200671	1.01228
SLC38A10	0.0558161	1.01228
KCNN4	0.00163057	1.01226
GCC2	0.0163786	1.01222
SLU7	0.060887	1.01219
C130071E11RIK	0.0131642	1.01218
C130060B01RIK	0.0340107	1.01215
TBC1D2	0.0536754	1.01215
HCFC2	0.08275	1.01214

**D230019K20RIK**	0.0278037	1.01208
**SPRED2**	0.0472716	1.01204
**ZFH4**	0.0336252	1.01203
**4833441J24RIK**	0.009669	1.01202
**6030408C04RIK**	0.0690266	1.012
**VPS39**	0.0807279	1.012
**LOC381105**	0.0233733	1.0119
**LOC381151**	0.0791336	1.01187
**D430036M17RIK**	0.042455	1.01183
**A530092L01RIK**	0.0328876	1.01177
**STX3**	0.0419809	1.01176
**STAG1**	0.0573785	1.01173
**AI447711**	0.0580281	1.01164
**2900058M19RIK**	0.0563903	1.01162
**5730408I11RIK**	0.033962	1.01161
**D030022P06RIK**	0.0388432	1.01156
**0610030E20RIK**	0.000170046	1.01155
**ASTE1**	0.0611768	1.01149
**TRAF5**	0.0178716	1.01147
**1200009F10RIK**	0.0789761	1.01144
**2210015D19RIK**	0.0789024	1.01137
**CTNND2**	0.0721662	1.01135
**RNF219**	0.018929	1.01131
**LOC624784**	0.00513373	1.01125
**A330068P14RIK**	0.0615363	1.01116
**2810409M01RIK**	0.0697388	1.01116
**TIPIN**	0.00133987	1.01112
**TAGAP1**	0.0301784	1.01112
**C130020O07RIK**	0.0291369	1.01109
**GTF3C4**	0.0119399	1.01103
**RNF121**	0.0562547	1.01094
**6430407D20RIK**	0.034877	1.01091
**MBIP**	0.0793964	1.01091
**1810020C02RIK**	0.0715178	1.01089
**D430024I10RIK**	0.0318659	1.01082
**D130067N01RIK**	0.0453786	1.0108
**4832410F06RIK**	0.0410661	1.01077
**ZYG11B**	0.077687	1.01076
**LARP2**	0.0216182	1.01074
**SCL0001923.1_57**	0.0265131	1.01069
**BTBD3**	0.0735594	1.01068
**E330009F12RIK**	0.0372664	1.01065
**4932438A13RIK**	0.0473671	1.01063
**TDP1**	0.0450169	1.01062
**2310047B19RIK**	0.0816645	1.01061
**1700001J03RIK**	0.00997392	1.01059
**C330016F22RIK**	0.015073	1.01057
**TWISTNB**	0.0628933	1.0105
**CCDC98**	0.0508384	1.01049
**6430544C07RIK**	0.0590456	1.01049
**MAD**	0.076803	1.01049
**ENPP4**	0.0134369	1.01048
**A430043M19RIK**	0.0255754	1.01047
**DCUN1D3**	0.0535564	1.01046
**D430003P20RIK**	0.0693993	1.01046
**C230055K05RIK**	0.0126059	1.01045
**4732431J01RIK**	0.0151849	1.01039
**4930438O05RIK**	0.0704534	1.01039

**SPC24**	0.031755	1.01037
**LOC380753**	0.0114541	1.01034
**D330027L06RIK**	0.0449861	1.01033
**EDC4**	0.0814037	1.01033
**9630009L01RIK**	0.0638693	1.01031
**ZFP593**	0.00615701	1.01027
**LOC384065**	0.00971614	1.01023
**TXNDC11**	0.0268672	1.01017
**BC014805**	0.0437354	1.01017
**2900075B16RIK**	0.0101867	1.01016
**MKLN1**	0.0457476	1.01014
**B230216G23RIK**	0.0507838	1.01011
**ZZEF1**	0.00965584	1.01009
**A630008E13RIK**	0.000631635	1.01003
**0610007P08RIK**	0.0154307	1.01001
**THEM4**	0.0583301	1.00992
**A830021G05RIK**	0.0213123	1.00988
**A130035O14RIK**	0.0250187	1.00987
**A930006A19RIK**	0.081983	1.00978
**CTDP1**	0.0160588	1.00975
**TNKS**	0.0434957	1.00972
**4833409N03RIK**	0.0518862	1.00972
**LOC382050**	0.07924	1.00972
**RNF20**	0.0579921	1.0097
**NUDCD2**	0.0643491	1.00968
**B4GALT7**	0.0338625	1.00967
**SRRM1**	0.0555908	1.00965
**ORC4L**	0.0734165	1.00965
**LOC386168**	0.0194886	1.0096
**2700063P19RIK**	0.0255443	1.00959
**SLC4A4**	0.00898954	1.00957
**LOC100044862**	0.0770487	1.00947
**LIG4**	0.00638069	1.00944
**D330038O06RIK**	0.0329911	1.00943
**A630042M04RIK**	0.0728805	1.00943
**C230048N02RIK**	0.0748825	1.00938
**A430106G13RIK**	0.0825211	1.00938
**TIPARP**	0.0315161	1.00936
**A930015K17RIK**	0.026982	1.00935
**3110023E09RIK**	0.0594373	1.00931
**A730062O07RIK**	0.0625772	1.00928
**6030419C18RIK**	0.0334572	1.00925
**RNF12**	0.0259638	1.0092
**BC016423**	0.0822131	1.0092
**HCCS**	0.0403072	1.00919
**LOC386298**	0.050905	1.00916
**RINT1**	0.00649261	1.00914
**A930030D01RIK**	0.00293358	1.0091
**4932441N08RIK**	0.0227116	1.0091
**F730023C13RIK**	0.0466136	1.00908
**SCL0002312.1_1**	0.0836941	1.00908
**A330081F11RIK**	0.0380831	1.00904
**D030073C20RIK**	0.0743426	1.00903
**XPO4**	0.0379081	1.00902
**4632411B12RIK**	0.0276272	1.009
**E330037I15RIK**	0.0261964	1.00898
**RAVER2**	0.0323649	1.00897
**LOC100040968**	0.0726725	1.00896

SPIRE1	0.0249011	1.00891
D130051G04RIK	0.0300017	1.00891
ECAC2	0.0523275	1.00891
KRT42	0.00116262	1.00886
EHD4	0.0172975	1.00886
A630007B06RIK	0.0331338	1.00886
MPHOSPH9	0.0700256	1.00885
1700105P06RIK	0.0800311	1.00883
AI597479	0.0817838	1.00881
9530014D17RIK	0.0614463	1.00877
LOC241593	0.0449687	1.00864
F730011C10RIK	0.0302803	1.00862
SNORA69	0.0609598	1.00855
G430079N04RIK	0.01906	1.00854
4632408O18RIK	0.0276802	1.00854
DCBLD2	0.0242933	1.00853
4833441B18RIK	0.0411813	1.00849
TDPOZ2	0.0219501	1.00847
B930066N23RIK	0.0609131	1.00847
NFATC1	0.0698867	1.00843
4933407M15RIK	0.0200953	1.00842
GM336	0.0804111	1.00842
LOC381890	0.0489472	1.00838
LOC100048439	0.0168844	1.00829
PTPRM	0.0236428	1.00829
A530016E13RIK	0.0570836	1.00825
PROSC	0.0769815	1.00824
EEA1	0.00174441	1.00818
HRB2	0.0758658	1.00818
ATP8A1	0.0340868	1.00817
N4BP2	0.0649777	1.00817
A030001A03RIK	0.0099531	1.00815
6430590A07RIK	0.0677812	1.00814
5830417I10RIK	0.0566788	1.00812
LOC242842	0.0208439	1.00811
TAF5	0.0317046	1.0081
C730037B14RIK	0.0194208	1.00807
NSCCN1	0.0203968	1.00803
CDK7	0.0265292	1.00802
D330004B08RIK	0.0779576	1.00802
LOC217591	0.0392964	1.00801
PDCL	0.0200156	1.00799
A530095B06RIK	0.0791694	1.00799
6030495H03RIK	0.0591504	1.00796
TERF2	0.07092	1.00793
4931406H21RIK	0.0609644	1.00792
5830411J07RIK	0.00698124	1.0079
9626100_224_RC	0.00343967	1.00788
3110021A11RIK	0.0606699	1.00787
LOC100039011	0.0311374	1.00785
D230032O14RIK	0.0148441	1.00784
A430103B12RIK	0.0388653	1.00784
PCDHB15	0.0123762	1.00782
LDLC	0.0316959	1.00782
YARS	0.0427415	1.00781
A630034D18RIK	0.0507157	1.00779
2200005K02RIK	0.0778254	1.00774
A930033H14RIK	0.0546111	1.00773

**A630084C02RIK**	0.0211043	1.00767
**EFNB3**	0.055074	1.00764
**A930009M04RIK**	0.00789842	1.00762
**KRR1**	0.00413376	1.00762
**LOC381539**	0.00317044	1.00761
**2310040B03RIK**	0.0420987	1.00759
**LOC236749**	0.0398673	1.00758
**B930074N15RIK**	0.027466	1.00757
**GORASP1**	0.0612277	1.00757
**A730042J05RIK**	0.0682708	1.00757
**D930022F04RIK**	0.0018738	1.00756
**6430403C09RIK**	0.0399237	1.00756
**F8**	0.0839939	1.00753
**D130054H18RIK**	0.0104359	1.0075
**ZBTB3**	0.0071152	1.00749
**1700012O15RIK**	0.0787792	1.00747
**A130094J16RIK**	0.029329	1.00746
**A430109E24RIK**	0.0761551	1.00745
**SERPINA1A**	0.0723815	1.00743
**NHLRC3**	0.0477616	1.00741
**C130020P08RIK**	0.0161403	1.0074
**C230021J06RIK**	0.0319103	1.00739
**MTDNA_ND6**	0.0385952	1.00739
**A930015C19RIK**	0.0200323	1.00738
**PITPNM3**	0.0193005	1.00736
**A930024F09RIK**	0.00130604	1.00733
**A830026L17RIK**	0.0516391	1.00733
**D130050K19RIK**	0.0197118	1.00732
**FBP1**	0.076245	1.0073
**PTGDS**	0.0640467	1.00729
**KLK10**	0.0383807	1.00728
**2810049C16RIK**	0.0500253	1.00728
**DOK5**	0.015454	1.00727
**C130005N09RIK**	0.0281078	1.00725
**ZFP184**	0.0308468	1.00716
**C530045P18RIK**	0.0357596	1.00716
**6720480F11RIK**	0.0588722	1.00716
**1500002C15RIK**	0.071321	1.00715
**B130006D01RIK**	0.0129369	1.00713
**9630050A07RIK**	0.0800557	1.00712
**ATXN7L1**	0.0687739	1.00711
**LOC207787**	0.00194813	1.00707
**C530043K16RIK**	0.0272073	1.00703
**4933437K13RIK**	0.0835296	1.00703
**BNIP3**	0.0689372	1.00702
**COX8B**	0.0827281	1.007
**FBXO41**	0.0212233	1.00697
**F730028J12RIK**	0.00993164	1.00695
**D030028A08RIK**	0.0260229	1.00695
**B230340J04RIK**	0.0303938	1.00694
**3110045I18RIK**	0.0823763	1.00692
**CPSF4**	5.86E-05	1.00691
**NT5DC3**	0.00802862	1.00688
**D19ERTD737E**	0.0246457	1.00688
**C030005D05RIK**	0.0722908	1.00687
**B930080B11RIK**	0.0321405	1.00685
**DDX23**	0.0531302	1.00685
**D930015M12RIK**	0.0243568	1.00684

**KCNMA1**	0.024551	1.00684
**UBOX5**	0.0813278	1.00682
**LOC386256**	0.0186455	1.00681
**MIA3**	0.0244647	1.00679
**LOC226439**	0.0149298	1.00678
**DDX19B**	0.0535148	1.00678
**5730405D16RIK**	0.0321558	1.00677
**2310002A08RIK**	0.00957841	1.00674
**A130028H19RIK**	0.0411291	1.00672
**ATF6**	0.00428788	1.0067
**PRPSAP2**	0.0122552	1.00669
**9430025M13RIK**	0.0283911	1.00669
**6446580_669**	0.0749812	1.00666
**E330014F24RIK**	0.0507051	1.00664
**LOC381400**	0.0398276	1.0066
**0710007C18RIK**	0.0317028	1.00657
**9330155G14RIK**	0.0427598	1.00653
**3230401M21RIK**	0.0500189	1.00651
**ETL1**	0.0539874	1.00651
**BC003993**	0.0105356	1.00645
**TRIM60**	0.0227589	1.00645
**NEK4**	0.000654828	1.00643
**SLC14A1**	0.00908298	1.00643
**1300013B24RIK**	0.0503448	1.0064
**LOC269213**	0.0634689	1.0064
**3732409C05RIK**	0.0108082	1.00639
**LOC100041194**	0.0183418	1.00638
**A730069A04RIK**	0.0767884	1.00637
**A630085E16RIK**	0.0335105	1.00636
**GABARAPL2**	0.0526189	1.00634
**D330004F16RIK**	0.0103905	1.00633
**LCE3F**	0.0745815	1.00633
**9430025L01RIK**	0.0135897	1.00631
**D930031I08RIK**	0.0534342	1.0063
**TMEM201**	0.0448017	1.00628
**B130065N20RIK**	0.0785801	1.00628
**E230017J10RIK**	0.00626852	1.00627
**5730585A16RIK**	0.021845	1.00627
**D330050H21RIK**	0.0367574	1.00627
**CYP2D12**	0.0811755	1.00625
**2610028F08RIK**	0.0651077	1.00623
**SH2D6**	0.013518	1.00622
**MASS1**	0.0276737	1.00622
**LOC381305**	0.0792035	1.00622
**6430519M23RIK**	0.0520442	1.00621
**HTR6**	0.0596764	1.0062
**UBE2A**	0.00597471	1.00619
**A530058L02RIK**	0.0673831	1.00619
**CPSF2**	0.0311212	1.00618
**A630086P05RIK**	0.0844846	1.00618
**LOC208080**	0.00285828	1.00616
**PGM3**	0.0171312	1.00616
**9430020K01RIK**	0.0229727	1.00615
**LOC383272**	0.0784993	1.00615
**APH1A**	0.0132558	1.00614
**PROSAPIP1**	0.0735192	1.00613
**TBKBP1**	0.0221469	1.00612
**9330112I12RIK**	0.0575839	1.00612

D330001K12RIK	0.0261612	1.00611
6620401C13RIK	0.0527089	1.00611
LOC382100	0.000359602	1.0061
A430059D01RIK	0.075581	1.0061
FIGLA	0.00378749	1.00609
A930026H04RIK	0.0289257	1.00608
LOC272683	0.0695297	1.00608
TSC1	0.0579482	1.00607
LOC333621	0.00673204	1.00606
G630008C18RIK	0.0403384	1.00606
C130086J11RIK	0.0523908	1.00605
LOC382091	0.000488847	1.00604
CNN1	0.0700222	1.00604
4921531D08RIK	0.0779654	1.00604
A030003K21RIK	0.0589725	1.00602
LOC100048710	0.0328485	1.00601
4930503L19RIK	0.0759599	1.00601
C230043E16RIK	0.081663	1.00599
LOC386196	0.0455529	1.00598
SLURP1	0.0424083	1.00597
D330035K16RIK	0.0164647	1.00596
CCDC57	0.0522858	1.00596
SEC22C	0.0176557	1.00595
CDC42SE2	0.0583743	1.00595
FPR-RS7	0.0267222	1.00594
RASGEF1B	0.0720794	1.00594
2510010K19RIK	0.0709361	1.00592
5031409G22RIK	0.0715013	1.00592
B230371M02RIK	0.0793973	1.00592
SCL000309.1_341	0.0615038	1.00591
LOC381380	0.0110722	1.00587
CALML4	0.0310244	1.00587
5730530J16RIK	0.00320154	1.00586
AP1GBP1	0.0291519	1.00586
B130065L01	0.0535602	1.00586
EG545124	0.0456183	1.00585
D130035M05RIK	0.070168	1.00585
LOC384496	0.00346445	1.00584
TGFA	0.0700404	1.00583
4921517D21RIK	0.0194366	1.00582
A930014B11RIK	0.0691394	1.00582
IGKV4-80_AJ231213_IG_KAPPA_VARIABLE_4-80_91	0.0205704	1.00581
1200016L19RIK	0.0725302	1.00581
D330017D17RIK	0.0309545	1.00579
BC027582	0.0771587	1.00577
D930012N15RIK	0.0185192	1.00576
DDX10	0.0842566	1.00576
LCN3	0.0731998	1.00575
D030028O16RIK	0.00781554	1.00574
2900052M09RIK	0.0183789	1.00574
SHC3	0.0302161	1.00574
C130066G14RIK	0.0806763	1.00574
C730004I03RIK	0.0567095	1.00573
LOC384086	0.00292476	1.00572
KLB	0.0153876	1.00572
A530010L13RIK	0.0636685	1.00572
5031409G23	0.0718414	1.00572
2900016B01RIK	0.0592728	1.00571

**B930008G09RIK**	0.0465116	1.0057
**TAS2R144**	0.0316419	1.00568
**LOC384847**	0.0612387	1.00568
**LOC381388**	0.06915	1.00568
**TRIM2**	0.0800647	1.00568
**LCN10**	0.0152989	1.00567
**LOC386476**	0.0476706	1.00567
**EG237009**	0.0572518	1.00567
**LOC626347**	0.0091363	1.00566
**4922502B01RIK**	0.0231753	1.00566
**B930044G13RIK**	0.0464722	1.00563
**NR5A2**	0.0273027	1.00562
**LOC668863**	0.0693668	1.00562
**RNF186**	0.0661583	1.00561
**D10627**	0.0686074	1.0056
**LOC381552**	0.0211034	1.00559
**BC052360**	0.0334585	1.00559
**CEP1**	0.0378469	1.00559
**4833427G06RIK**	0.0456853	1.00559
**A630095E13RIK**	0.0122683	1.00558
**COIL**	0.0328209	1.00558
**2610010G17RIK**	0.0322707	1.00556
**INPP4B**	0.0341567	1.00555
**A230072B04RIK**	0.0557636	1.00553
**6720407G21RIK**	0.0379962	1.00552
**BAZ2A**	0.0366202	1.00551
**1700012F10RIK**	0.0602456	1.0055
**4933413N12RIK**	0.080735	1.0055
**2310079F23RIK**	0.0410672	1.00549
**PRRG4**	0.00877329	1.00546
**OLFR477**	0.0540611	1.00546
**EG546150**	0.00209201	1.00545
**SPINK5**	0.012304	1.00545
**IGHV5S15_AF290966_IG_HEAVY_VARIABLE_5S15_49**	0.0430605	1.00545
**PYCR1**	0.0633214	1.00541
**A630008H02RIK**	0.0760884	1.00541
**H2LS**	0.0214231	1.0054
**E030040E19RIK**	0.0815833	1.0054
**D030065B14RIK**	0.0574538	1.00538
**KCTD11**	0.0574956	1.00538
**PELI2**	0.0297862	1.00536
**NEIL3**	0.0117519	1.00534
**MPP4**	0.0849504	1.00534
**AV273951**	0.0239095	1.00533
**A130071D18RIK**	0.031183	1.00533
**OLFR131**	0.0804586	1.00533
**EG386551**	0.0191256	1.00532
**LOC383074**	0.00897783	1.00531
**LOC386273**	0.0139368	1.00531
**NEK7**	0.0693452	1.00531
**TCF15**	0.0118113	1.0053
**ANK1**	0.0372429	1.0053
**NR3C1**	0.00605462	1.00529
**OLFR1263**	0.000701285	1.00529
**SERPINB1B**	0.000564435	1.00529
**OLFR689**	0.00869432	1.00528
**LOC327767**	0.0332929	1.00528
**SORBS2**	0.00633566	1.00527

SCL0002720.1_68	0.0348022	1.00524
9430002A10RIK	0.053213	1.00524
A630082N15RIK	0.0457292	1.00523
1700021A07RIK	0.0589247	1.00523
CORO7	0.059055	1.00523
4833406C17RIK	0.0699317	1.00523
G430005B15RIK	0.047265	1.00522
NFKBIB	0.0614745	1.0052
A130095C03RIK	0.0630871	1.0052
IGHV7S2_J00500_IG_HEAVY_VARIABLE_7S2_59	0.0269792	1.00519
9830134N18RIK	0.021906	1.00518
IL20	0.0267641	1.00518
TSSK5	0.0794624	1.00516
SLC25A46	0.0620987	1.00515
SPIB	0.0305958	1.00514
MAGEL2	0.071513	1.00512
SLC17A1	0.0730602	1.00511
5033424B07RIK	0.0472812	1.0051
9930005E07RIK	0.0631855	1.0051
OLFR50	0.0771642	1.00508
SCL0003381.1_13	0.0120425	1.00507
LOC381444	0.070422	1.00507
FAM107A	0.0778007	1.00507
BC021790	0.0791212	1.00507
A730027B03RIK	0.00128183	1.00506
LOC100045524	0.0587181	1.00505
LOC386117	0.0743022	1.00504
E430039K24RIK	0.0237459	1.00503
FBXO16	0.0642267	1.00503
4632419K20RIK	0.0172857	1.00502
GFM	0.0217698	1.00502
9230103K20RIK	0.0743983	1.00502
IIGP1	0.000182894	1.005
LOC383049	0.0464134	1.00499
HEATR5A	0.0622071	1.00499
FOXL2	0.0127498	1.00498
KRT79	0.0269727	1.00498
MEFV	0.00823271	1.00497
GPR150	0.0705876	1.00496
XEDAR EDA-A2R	0.0360307	1.00495
KLK1	0.072205	1.00495
D130035C10RIK	0.0343768	1.00493
LOC240761	0.00632432	1.00492
CASKIN1	0.0276887	1.00492
EMU2-PENDING	0.0533032	1.00492
LOC386442	0.0711015	1.00492
KIF21B	0.0143899	1.00491
1700124K17RIK	0.0435026	1.00491
SRD5A2L2	0.0462563	1.00491
EG433634	0.061472	1.0049
5730596B20RIK	0.0229307	1.00489
LOC277046	0.00346284	1.00488
SIX2	0.0165063	1.00488
LOC195534	0.0687897	1.00488
2010109K11RIK	0.0697838	1.00488
EIF2C3	0.0624258	1.00487
OLFR1355	0.0500545	1.00486
LOC100039829	0.032425	1.00485

**D5ERTD40E**	0.0279559	1.00484
**LANCL3**	0.0341336	1.00484
**GM73**	0.0672044	1.00484
**LOC380691**	0.0111517	1.00483
**OTTMUSG00000012631**	0.0140379	1.00483
**NANP**	0.0253573	1.00483
**3010022N24RIK**	0.0572901	1.00483
**LOC385368**	0.0653895	1.00483
**2500001N03RIK**	0.0673952	1.00483
**LOC637337**	0.0242839	1.00482
**C230059C13RIK**	0.0556214	1.00482
**2610528A11RIK**	0.0567537	1.00482
**6030447G11RIK**	0.0667444	1.00482
**LOC266459**	0.081542	1.00482
**LOC677473**	0.066477	1.00481
**LOC380869**	0.080792	1.00481
**A330040H08RIK**	0.00523696	1.0048
**LOC383856**	0.0502791	1.00479
**SLC9A9**	0.0395859	1.00478
**4933440H19RIK**	0.0841447	1.00478
**HOXD10**	0.0235112	1.00476
**RPA2**	0.0805706	1.00476
**MMP28**	0.00538002	1.00475
**GPM6B**	0.0143201	1.00475
**SLC43A3**	0.0591088	1.00474
**LOC100047923**	0.0665521	1.00473
**A130022A09RIK**	0.0699132	1.00473
**C230060F13RIK**	0.00864836	1.00472
**0610010K14RIK**	0.0282735	1.00472
**PARD3B**	0.0846703	1.0047
**CYPT6**	0.0354781	1.00469
**SPAG1**	0.0481972	1.00469
**BARD1**	0.0653003	1.00469
**BC024814**	0.0716392	1.00468
**H2-T18**	0.0250404	1.00467
**GPR142**	0.0262958	1.00467
**AI854517**	0.0288775	1.00467
**OLFR1166**	0.0346123	1.00467
**ZNF142**	0.0727249	1.00467
**4930515K21RIK**	0.0543976	1.00466
**SCL0003499.1_101**	0.000659747	1.00465
**OLFR741**	0.00161766	1.00463
**MAP3K9**	0.0343341	1.00463
**SP8**	0.035062	1.00463
**TIEG**	0.0351223	1.00463
**5730437O09RIK**	0.0653445	1.00463
**CCDC142**	0.0702712	1.00463
**ABCG5**	0.0143511	1.00462
**ZFH4-PENDING**	0.0249424	1.00462
**6330532E14RIK**	0.036012	1.00462
**5730590C14RIK**	0.0505488	1.00462
**AGXT**	0.071099	1.00462
**LOC270366**	0.0744055	1.00462
**STAR**	0.0553452	1.00461
**GIMAP5**	0.065452	1.00461
**PDE5A**	0.00782967	1.0046
**KLK1B22**	0.0625392	1.0046
**LOC384082**	0.0769467	1.0046

**A230055O04RIK**	0.0273967	1.00459
**LOC329277**	0.0318957	1.00459
**D630024I10RIK**	0.00887149	1.00458
**E130311K13RIK**	0.0470058	1.00458
**LOC637047**	0.0618495	1.00458
**LOC328820**	0.0107278	1.00456
**MUC20**	0.0793576	1.00456
**C820012K02RIK**	0.0480102	1.00455
**A130024C22RIK**	0.0553834	1.00455
**NOX1**	0.0606304	1.00455
**KLRAQ1**	0.00160915	1.00454
**KRTAP6-3**	0.059088	1.00454
**9030203C11RIK**	0.0282453	1.00453
**4930415C24RIK**	0.00672877	1.00452
**COL4A3**	0.0809952	1.00452
**CALCRL**	0.0254814	1.00451
**SOHLH2**	0.0546602	1.00451
**E230017C09RIK**	0.0482363	1.0045
**LOC232065**	0.0155654	1.00449
**4930584N22RIK**	0.0276983	1.00448
**SAST-PENDING**	0.0576549	1.00448
**B230312E22RIK**	0.0613893	1.00448
**LOC243118**	0.0289992	1.00447
**OLFR730**	0.0537088	1.00447
**GGA3**	0.0498031	1.00446
**OLFR1509**	0.065264	1.00446
**A430042F24RIK**	0.0117788	1.00445
**C130087G11RIK**	0.0251662	1.00445
**E130010M05RIK**	0.0330943	1.00445
**LOC100047738**	0.0361007	1.00444
**EMILIN1**	0.0427275	1.00444
**OLFR697**	0.0514076	1.00443
**KRTAP4-7**	0.0724485	1.00443
**CTS8**	0.0726637	1.00443
**1810018F18RIK**	0.0829344	1.00443
**LOC241962**	0.00931447	1.00442
**LOC626150**	0.0488919	1.00442
**LOC233710**	0.0817578	1.00442
**A330072B11RIK**	0.023903	1.00441
**PLA2G6**	0.0313623	1.00441
**LOC328091**	0.0686269	1.00441
**RWDD2B**	0.0719125	1.00441
**GNGT1**	0.0844747	1.00441
**NKX2-6**	0.05959	1.0044
**HCN1**	0.0375905	1.00439
**C6**	0.0693917	1.00438
**MTAP7D3**	0.0565733	1.00437
**PALM2**	0.0577289	1.00437
**FGF23**	0.00844743	1.00436
**2310058A03RIK**	0.0281115	1.00436
**ADAT1**	0.0443353	1.00436
**1700007K09RIK**	0.0218747	1.00435
**UNC13H1**	0.0272768	1.00435
**CLNK**	0.0404547	1.00435
**B230206H07RIK**	0.0352696	1.00433
**TOPBP1**	0.0840063	1.00433
**LOC628994**	0.0423014	1.00432
**CKAP4**	0.0428217	1.00432

**D930021N14**	0.0465148	1.00432
**COL28A1**	0.0829229	1.00432
**KATNAL1**	0.0570141	1.00431
**D930039D09RIK**	0.0631192	1.00431
**9530020O07RIK**	0.0608333	1.0043
**LOC385817**	0.0818781	1.0043
**RLN3**	0.0039309	1.00429
**9430041J06RIK**	0.0494628	1.00429
**LOC100039753**	0.0689455	1.00429
**1600010O03RIK**	0.0697729	1.00429
**4931422A14RIK**	0.0115805	1.00426
**QSOX2**	0.0588064	1.00426
**CD5L**	0.0708235	1.00426
**B930032P17RIK**	0.0258434	1.00425
**9530014N24RIK**	0.0574545	1.00425
**OLFR104**	0.0794391	1.00424
**LOC210633**	0.0804235	1.00424
**OLFR204**	0.0518749	1.00423
**OLFR1168**	0.0837773	1.00423
**ZPBP2**	0.0410605	1.00422
**TOX2**	0.0507339	1.00421
**INVS**	0.0105041	1.0042
**SCL0003231.1_13**	0.037939	1.0042
**OTTMUSG00000000480**	0.0833788	1.0042
**LOC242607**	0.057895	1.00419
**B230325K09RIK**	0.0355509	1.00418
**EEF1A2**	0.0831434	1.00418
**TEX101**	0.0759289	1.00417
**MIXL1**	0.0842436	1.00417
**LOC382183**	0.0276931	1.00416
**A730091E08RIK**	0.0370235	1.00416
**D830007B15RIK**	0.0181818	1.00415
**TNFRSF1B**	0.0411195	1.00415
**HMBOX1**	0.0433938	1.00415
**TYMP**	0.054423	1.00415
**KIFC5C**	0.0685617	1.00415
**4933402G07RIK**	0.0843493	1.00414
**C430002M09RIK**	0.0071347	1.00413
**SCL0003936.1_5**	0.0205323	1.00413
**LOC381867**	0.0464177	1.00413
**F730005M08RIK**	0.0468353	1.00413
**D130067I07RIK**	0.0693562	1.00413
**2410137M14RIK**	0.0789006	1.00413
**B630006N21RIK**	0.00756073	1.00412
**ERMN**	0.0249605	1.00412
**8430404H04RIK**	0.0708833	1.00412
**A530046M15**	0.0770587	1.00412
**C230060D12RIK**	0.0537743	1.00411
**LOC383001**	0.0505014	1.0041
**ITIH5L**	0.0839699	1.0041
**CPT1A**	0.00221673	1.00409
**ERAS**	0.0182861	1.00409
**9845300_5604**	0.0445206	1.00409
**FTMT**	0.0701861	1.00409
**OTOR**	0.0358731	1.00408
**A730041H09RIK**	0.0466123	1.00408
**BANK1**	0.0710409	1.00408
**LOC384469**	0.00626151	1.00406

**AI842396**	0.0623226	1.00406
**TAS2R129**	0.0642622	1.00406
**UGT2B5**	0.0643938	1.00406
**CES7**	0.00560057	1.00405
**LOC100047007**	0.0170538	1.00405
**6330514O11RIK**	0.0412415	1.00405
**FUT11**	0.0450958	1.00405
**2810048G17RIK**	0.0710421	1.00405
**1110033F04RIK**	0.00460167	1.00404
**LOC229958**	0.0421716	1.00404
**LOC242377**	0.0117489	1.00403
**A830027N12RIK**	0.0382202	1.00403
**LOC383383**	0.045522	1.00403
**SRP19**	0.0727186	1.00403
**EG626305**	0.0819941	1.00403
**LOC330474**	0.00249524	1.00402
**KCND3**	0.0281621	1.00402
**NTN2L**	0.043926	1.00402
**LOC383494**	0.0528848	1.00401
**TCF1**	0.0620548	1.00401
**LOC381129**	0.0813443	1.004
**SCL0001874.1_222**	0.0346116	1.00399
**SCL0003052.1_338**	0.0125444	1.00398
**MMP7**	0.0545845	1.00398
**OLFR1416**	0.06271	1.00398
**SERPINA1C**	0.00833577	1.00397
**BUB3**	0.0245687	1.00397
**6430590A10RIK**	0.00310773	1.00396
**E030001K11RIK**	0.0262262	1.00396
**DDX56**	0.0581545	1.00396
**PABPNL1**	0.0128541	1.00395
**0610038F15RIK**	0.0252871	1.00395
**TOLLIP-PENDING**	0.0377965	1.00394
**LOC381420**	0.0398257	1.00394
**WDR66**	0.0106836	1.00393
**SMTNL1**	0.0447409	1.00393
**CSTL1**	0.0475462	1.00393
**GAS2**	0.00879374	1.00392
**OOG4**	0.0199163	1.00392
**2410080I02RIK**	0.0373515	1.00392
**1700041I07RIK**	0.0672531	1.00392
**LOC239556**	0.0217285	1.00391
**SEZ6**	0.0819942	1.00391
**CNBP**	0.0632044	1.0039
**RAI3**	0.0598405	1.00389
**V1RE9**	0.0641056	1.00389
**RAB2B**	0.0827943	1.00389
**LCE1M**	0.00220902	1.00388
**SIM1**	0.0172718	1.00388
**MJ-7000-176_2342**	0.0402782	1.00388
**ANAPC11**	0.0676049	1.00388
**4933427G17RIK**	0.0757093	1.00388
**OLFR630**	0.0281882	1.00387
**0610035N01RIK**	0.0527268	1.00387
**A330088F01RIK**	0.0747181	1.00387
**TELO2**	0.0765847	1.00387
**KIF11**	0.0366694	1.00386
**1700017D01RIK**	0.0425587	1.00386

**LOC385054**	0.0587604	1.00386
**LOC271709**	0.0261441	1.00385
**ELL2**	0.0471052	1.00385
**NPTXR**	0.0631726	1.00384
**D130098J21RIK**	0.00809442	1.00383
**LOC100047053**	0.0695519	1.00383
**2510025K24RIK**	0.0709102	1.00383
**D6MIT97**	0.0399305	1.00382
**FUT7**	0.0146676	1.00381
**CTRB1**	0.00464216	1.0038
**ESRRBL1**	0.0466219	1.0038
**A930029O05RIK**	0.0727495	1.0038
**L3MBTL**	0.0833767	1.0038
**SCL000877.1_11**	0.00662479	1.00379
**SLC7A12**	0.02812	1.00378
**LOC382001**	0.0311116	1.00378
**E230013J01RIK**	0.0360744	1.00378
**B430320O11RIK**	0.00556684	1.00377
**C030003M13RIK**	0.0553337	1.00377
**6230412O07RIK**	0.0553626	1.00377
**SCL0002531.1_14**	0.00442142	1.00376
**PM20D1**	0.0293588	1.00376
**LOC674008**	0.00625863	1.00375
**LOC381804**	0.0108813	1.00375
**RBKS**	0.0460135	1.00375
**EG432985**	0.0759399	1.00375
**TYW3**	0.00614016	1.00374
**PHLPPL**	0.0225343	1.00374
**MADCAM1**	0.0431036	1.00374
**SCL0001333.1_125**	0.00537982	1.00373
**8030481M12RIK**	0.044536	1.00373
**4732485D01RIK**	0.0670356	1.00373
**LOC385615**	0.0801035	1.00373
**H2-T3**	0.00610559	1.00372
**LOC100045284**	0.0295372	1.00372
**RBM22**	0.0634216	1.00372
**1110049N09RIK**	0.0150417	1.00371
**EXOSC9**	0.0367922	1.00371
**CPT1B**	0.0393093	1.00371
**LOC330134**	0.0529709	1.00371
**D430029B19RIK**	0.0139308	1.0037
**LOC384585**	0.0251304	1.0037
**TRIM38**	0.0587338	1.00369
**LOC383503**	0.0700611	1.00369
**1700030J15RIK**	0.0737918	1.00369
**OLFR955**	0.00233127	1.00368
**OLFR794**	0.0127356	1.00368
**ADAMTSL3**	0.0183519	1.00368
**LOC676929**	0.0471372	1.00368
**1700055M20RIK**	0.0713016	1.00368
**D430034A07RIK**	0.0609179	1.00367
**STXBP3A**	0.0839699	1.00367
**VSX2**	0.036486	1.00366
**LOC381051**	0.0499275	1.00366
**LOC382867**	0.0771555	1.00366
**D130039D18RIK**	0.0403471	1.00365
**1500010C09RIK**	0.0638253	1.00365
**LOC331453**	0.0719782	1.00365

**LOC384412**	0.0303222	1.00364
**TICAM1**	0.0388569	1.00364
**OLFR706**	0.0454898	1.00364
**SPINK10**	0.0530194	1.00364
**LOC381198**	0.0691656	1.00364
**KLK1B26**	0.0749852	1.00364
**LOC382669**	0.00223429	1.00362
**OLFR1346**	0.035299	1.00362
**TGM6**	0.0473342	1.00362
**LOC384357**	0.0705217	1.00362
**KRT2**	0.0731067	1.00362
**BC042761**	0.080169	1.00362
**RAD51C**	0.00378255	1.00361
**IGSF5**	0.0608633	1.00361
**2010309J24RIK**	0.00620025	1.0036
**4932407I05**	0.0164258	1.0036
**B230362M20RIK**	0.0043624	1.00359
**LOC100046278**	0.00247547	1.00359
**OCM**	0.0484347	1.00358
**4930594M22RIK**	0.0532732	1.00358
**CAR6**	0.067741	1.00358
**CEACAM18**	0.0693365	1.00358
**ZFP612**	0.0768054	1.00358
**OTTMUSG00000000514**	0.0498662	1.00357
**LOC386154**	0.0602014	1.00357
**SCL000460.1_17**	0.06425	1.00357
**GM414**	0.0226482	1.00356
**MOR**	0.0604752	1.00356
**OLFR148**	0.0708917	1.00356
**OLFR474**	0.0675022	1.00355
**FBLN5**	0.0376346	1.00354
**KIF2A**	0.00553602	1.00353
**KLRA5**	0.00724827	1.00353
**LOC386379**	0.0311752	1.00353
**AI987692**	0.0433738	1.00353
**LOC671564**	0.0477086	1.00353
**D930040M24RIK**	0.0580352	1.00353
**LOC329055**	0.0817963	1.00353
**LOC100048112**	0.0788444	1.00352
**SCL0070954.1_214**	0.000655083	1.00351
**OLFR1182**	0.0282484	1.00351
**9230110C19RIK**	0.0429981	1.00351
**PCDH10**	0.0668244	1.00351
**E230026C15RIK**	0.0756238	1.00351
**LOC385977**	0.0805067	1.00351
**D930049F02RIK**	0.0347099	1.0035
**SEC8**	0.0363792	1.0035
**D730040F13RIK**	0.065144	1.0035
**NAAA**	0.05409	1.00349
**SLC19A3**	0.0626016	1.00349
**FGF9**	0.0681769	1.00349
**LOC381792**	0.0723545	1.00349
**1700023C21RIK**	0.0296173	1.00348
**LOC238447**	0.0551147	1.00348
**4930448I06RIK**	0.00465881	1.00347
**A630064F15RIK**	0.0349509	1.00347
**LOC381447**	0.0463211	1.00347
**GK5**	0.00342538	1.00346

**NAIF1**	9.21E-05	1.00345
**PADI3**	0.0694316	1.00345
**4931432E15RIK**	0.0188359	1.00344
**B130052F17RIK**	0.0495222	1.00344
**OLFR895**	0.0844573	1.00344
**LRP5**	0.0241465	1.00343
**LONRF2**	0.0494313	1.00343
**D630048A15RIK**	0.0616899	1.00343
**A530017D12RIK**	0.0806857	1.00343
**THAP2**	0.0226808	1.00342
**LOC381700**	0.0740016	1.00342
**SLC22A6**	0.0776208	1.00342
**LOC382358**	0.00718225	1.00341
**PLCD4**	0.0218155	1.00341
**LOC381977**	0.0573609	1.00341
**4930470H18RIK**	0.0137039	1.0034
**9130221D24RIK**	0.0340141	1.0034
**MS4A12**	0.037651	1.0034
**LOC384216**	0.0759756	1.0034
**HOXC10**	0.0466356	1.00339
**A630065D16RIK**	0.0489745	1.00339
**A930021K20RIK**	0.0518282	1.00339
**A430110N23RIK**	0.057073	1.00339
**TSGA13**	0.0254354	1.00338
**LOC381466**	0.040253	1.00338
**MDC1**	0.0469144	1.00338
**LOC382465**	0.04866	1.00338
**G630065C19RIK**	0.0846834	1.00338
**SAMD11**	0.0381626	1.00337
**SNORD22**	0.0501433	1.00337
**ARVCF**	0.0748622	1.00337
**NLRP9C**	0.0770665	1.00337
**PRRXL1**	0.00651601	1.00336
**A430105C13RIK**	0.0513226	1.00336
**LOC383047**	0.0766711	1.00336
**OLFR1513**	0.0767474	1.00336
**MKIAA1294**	0.0310201	1.00335
**1700096C12RIK**	0.0470596	1.00335
**1700014B07RIK**	0.0293369	1.00334
**FPR1**	0.0723094	1.00334
**2900060F21RIK**	0.0315308	1.00332
**SLC15A1**	0.0577757	1.00332
**LOC245069**	0.0579873	1.00332
**ZFP7**	0.00846134	1.00331
**OLFR1239**	0.0137064	1.00331
**ALDH1B1**	0.04342	1.00331
**6530409L22RIK**	0.00274285	1.0033
**LOC332175**	0.0157487	1.0033
**8430427K15**	0.0354671	1.0033
**AU014947**	0.0581238	1.0033
**5830411O09RIK**	0.0678698	1.0033
**LOC195281**	0.00748611	1.00329
**LOC384421**	0.00569844	1.00329
**KIF27**	0.0159663	1.00329
**D3WSU161E**	0.0264303	1.00329
**LOC384293**	0.0268939	1.00329
**8430406I07RIK**	0.0452061	1.00329
**INSR**	0.0530454	1.00329

NARG2	0.0558955	1.00328
2900005I04RIK	0.00326318	1.00327
4930451E10RIK	0.00893007	1.00327
OPTC	0.02431	1.00327
LOC382821	0.0427748	1.00327
LOC631071	0.0513202	1.00327
D330045A20RIK	0.0756963	1.00327
DEFB10	0.0132863	1.00326
OLFR381	0.0320056	1.00326
CLRN3	0.0111124	1.00325
EG240055	0.0301504	1.00325
LOC238564	0.0616227	1.00325
OLFR1463	0.00269857	1.00324
KCNK10	0.0254927	1.00324
DLEC1	0.0273282	1.00324
LOC380720	0.0448154	1.00324
AADACL3	0.0684578	1.00324
FCRH3	0.0788089	1.00324
ARL10A	0.0796979	1.00324
SPEER4F	0.0829338	1.00324
METTL10	0.00823876	1.00323
4933403G17RIK	0.0127708	1.00323
LOC268781	0.0134127	1.00323
4930474N05RIK	0.0249413	1.00323
C530048O03RIK	0.046972	1.00323
LOC382715	0.0581035	1.00323
PLSCR3	0.0612839	1.00323
TPTE	0.0259162	1.00322
RBL2	0.0403374	1.00322
TAAR7D	0.0609154	1.00322
6430598J10RIK	0.0810248	1.00322
VNN1	0.0839453	1.00322
LOC237403	0.0176126	1.00321
LOC381269	0.0440611	1.00321
APC7-PENDING	0.0541988	1.00321
LRRC69	0.00919606	1.0032
LOC100044443	0.0184194	1.0032
SFXN2	0.060343	1.0032
OLFR186	0.0364938	1.00319
TGM5	0.0419063	1.00319
V1RE3	0.0767419	1.00318
TTC9	0.0191091	1.00317
SPRR2J	0.0609854	1.00317
LASS3	0.0636428	1.00317
ZFP526	0.0250022	1.00315
LOC386398	0.0489099	1.00315
LOC330630	0.0823148	1.00315
OA1	0.0458371	1.00314
AK5	0.0489703	1.00314
PLN	0.0656491	1.00314
CD19	0.0671159	1.00312
9845300_2519	0.0830624	1.00312
9930039A11RIK	0.0558579	1.00311
LOC637754	0.0713194	1.0031
TTRAP	0.0357796	1.00309
REFBP2	0.0396603	1.00309
LOC240879	0.0662485	1.00309
RAD21	0.0676197	1.00309

**4930529I22RIK**	0.0826473	1.00309
**GNAL**	0.0836829	1.00309
**GYPA**	0.0630301	1.00308
**4930544M13RIK**	0.000164289	1.00307
**PNN**	0.0269905	1.00307
**TEP1**	0.0549351	1.00307
**LOC384769**	0.0675106	1.00307
**C630030K01RIK**	0.0736874	1.00307
**CLCN5**	0.0681423	1.00306
**A930103B21RIK**	0.0276608	1.00305
**SSFA1**	0.0741959	1.00305
**LOC100040962**	0.0326223	1.00304
**NAALADL1**	0.044607	1.00304
**2310021H06RIK**	0.0831672	1.00304
**OLFR112**	0.0240858	1.00303
**THSD2**	0.0370747	1.00303
**GATA5**	0.080805	1.00303
**1700036D21RIK**	0.0224153	1.00302
**STAG3**	0.0404577	1.00302
**CD300LG**	0.083347	1.00302
**LOC382008**	0.0203829	1.00301
**KCNA4**	0.037185	1.00301
**IGHV10S3_AF064446_IG_HEAVY_VARIABLE_10S3_9**	0.062814	1.00301
**PDE6A**	0.0170239	1.003
**LOC329240**	0.0175809	1.003
**5730419A02RIK**	0.026117	1.003
**FABP1**	0.0542088	1.003
**CES6**	0.0806743	1.003
**BC030307**	0.00268813	1.00299
**APBA2**	0.0205195	1.00299
**LOC385308**	0.061384	1.00299
**A930014D06RIK**	0.0653706	1.00299
**A430091G03RIK**	0.0203413	1.00298
**LOC330441**	0.0744127	1.00298
**1810011H11RIK**	0.0140092	1.00297
**4930556B16RIK**	0.0188252	1.00297
**OLFR883**	0.0215048	1.00297
**LOC383530**	0.0661897	1.00297
**KCNJ4**	0.0790195	1.00297
**SCFD2**	0.00622898	1.00296
**LOC384537**	0.0306014	1.00296
**C030019P07RIK**	0.0415892	1.00296
**1110006G14RIK**	0.0155137	1.00295
**1190020E20RIK**	0.0166506	1.00295
**V1RI5**	0.0294288	1.00295
**ZFP759**	0.040095	1.00295
**DMC1**	0.0686524	1.00295
**D1ERTD622E**	0.0711518	1.00295
**MYOD1**	0.0723168	1.00295
**F830048M02RIK**	0.0676255	1.00294
**LACTB2**	0.0188497	1.00293
**3110052M02RIK**	0.0286893	1.00293
**0610008F07RIK**	0.0526044	1.00293
**TEX22**	0.0592133	1.00293
**SENP5**	0.0652863	1.00293
**REX2**	0.0770347	1.00293
**1700013N18RIK**	0.0437316	1.00292
**LOC384616**	0.0804339	1.00292

**9430038G21RIK**	0.00229539	1.00291
**C230060L03RIK**	0.0282884	1.00291
**LOC383123**	0.0448433	1.00291
**D930018F03RIK**	0.00684435	1.0029
**OLFR1423**	0.0504189	1.0029
**KLHL11**	0.0431559	1.00289
**LOC328277**	0.083856	1.00289
**LOC100041729**	0.0290165	1.00288
**CHRM2**	0.0342631	1.00287
**A530006F08RIK**	0.0440159	1.00287
**2610109H07RIK**	0.0479134	1.00287
**A930104D05RIK**	0.0584331	1.00287
**LOC381790**	0.0303302	1.00286
**6530403M18RIK**	0.0184368	1.00285
**JAK1**	0.0654716	1.00285
**9626096_327_RC**	0.0257459	1.00284
**EPO**	0.0459427	1.00284
**PROCR**	0.0670525	1.00284
**2810046O15RIK**	0.0711477	1.00284
**POP1**	0.0722499	1.00284
**4930560E09RIK**	0.0812479	1.00284
**LOC382372**	0.0441865	1.00283
**OLFR1465**	0.0467221	1.00283
**FCRL5**	0.00375724	1.00282
**9629553_2023_RC**	0.0452006	1.00282
**HEATR3**	0.0499182	1.00282
**B230369F24RIK**	0.0191081	1.00281
**4933429E06RIK**	0.0263699	1.00281
**DNM3**	0.0414149	1.00281
**ORC1L**	0.0528632	1.00281
**MKL2**	0.0615158	1.00281
**D030055F01RIK**	0.0744671	1.00281
**MJ-1000-77_495**	0.0092454	1.0028
**DMPK**	0.055735	1.0028
**A830015D01RIK**	0.0473577	1.00279
**B430319F04RIK**	0.0176315	1.00278
**RESP18**	0.0736674	1.00278
**LRP1B**	0.0761777	1.00278
**CGB**	0.0809553	1.00278
**MPV17L**	0.00492281	1.00277
**LOC381992**	0.0376398	1.00277
**BC057022**	0.0215446	1.00276
**B130010N03RIK**	0.0394672	1.00276
**OLFR1289**	0.0586441	1.00276
**TUBGCP6**	0.00473198	1.00274
**LOC381452**	0.0201323	1.00274
**OLFR678**	0.0536572	1.00274
**GM749**	0.0536869	1.00274
**LOC240779**	0.00670592	1.00273
**2410089E03RIK**	0.026813	1.00273
**ACCN5**	0.0389459	1.00273
**OLFR401**	0.0418139	1.00273
**D630024B06RIK**	0.06074	1.00273
**OTTMUSG00000008911**	0.0825421	1.00273
**LOC218165**	0.0223311	1.00272
**TMPRSS11A**	0.0843766	1.00272
**LOC385037**	0.0788412	1.0027
**F830030F15RIK**	9.09E-05	1.00269

**AGTR2**	0.0384311	1.00269
**OTTMUSG00000005300**	0.00358615	1.00268
**CCDC77**	0.0100193	1.00268
**LOC546233**	0.0165813	1.00268
**GM378**	0.0717724	1.00268
**5730575G16RIK**	0.0751181	1.00268
**LOC229571**	0.0339347	1.00267
**TUBAL3**	0.0196643	1.00266
**USHBP1**	0.0395916	1.00266
**1810044B20RIK**	0.0419282	1.00266
**MJ-3000-106_2479**	0.0505745	1.00266
**SVA**	0.000806191	1.00265
**NPY5R**	0.0728333	1.00265
**C230059B01RIK**	0.0110764	1.00264
**AW555814**	0.0171433	1.00264
**HIST1H2AA**	0.0238146	1.00264
**G430046L24RIK**	0.0848629	1.00264
**3110001I22RIK**	0.00557256	1.00263
**LOC665432**	0.0401802	1.00263
**OLFR1441**	0.0762791	1.00263
**V1RG6**	0.00414284	1.00262
**TULP1**	0.0339846	1.00262
**1700108F19RIK**	0.0772581	1.00262
**LOC384457**	0.00470617	1.00261
**LOC211095**	0.0388662	1.00261
**COL9A3**	0.0695791	1.00261
**IGK-V**	0.0756577	1.00261
**F830018F06RIK**	0.0761352	1.00261
**4921535G17RIK**	0.000720944	1.0026
**F730046H10RIK**	0.0461902	1.0026
**NANOS3**	0.0525821	1.0026
**SYT2**	0.0527075	1.0026
**B430203M17RIK**	0.057317	1.0026
**OLFR1222**	0.0101007	1.00259
**LOC668030**	0.047158	1.00259
**NCOA1**	0.0475975	1.00259
**GUCY2E**	0.00655092	1.00258
**SERPINA10**	0.00634366	1.00258
**SLC6A13**	0.0237807	1.00258
**RHOX3F**	0.0565505	1.00258
**HRH2**	0.0750477	1.00258
**ADCY1**	0.077168	1.00258
**LOC100047710**	0.0192994	1.00257
**PRIM1**	0.0764276	1.00257
**2310046K01RIK**	0.0823422	1.00257
**LOC382815**	0.056659	1.00256
**LOC383111**	0.0799707	1.00256
**9330171B01RIK**	0.0135314	1.00255
**B930088D18RIK**	0.0236362	1.00255
**LOC218298**	0.0399872	1.00255
**SCL000959.1_2**	0.0532484	1.00255
**LY6F**	0.0567695	1.00255
**OLFR203**	0.053186	1.00254
**ABCB9**	0.0666021	1.00254
**TMEM7**	0.067609	1.00254
**G630039M15RIK**	0.00907281	1.00253
**CACNG3**	0.011505	1.00253
**E030037J05RIK**	0.0262573	1.00253

**4930542C12RIK**	0.0300936	1.00253
**LOC232577**	0.0330862	1.00253
**A830007N09RIK**	0.00497499	1.00252
**TDRD1**	0.0072045	1.00252
**HCLS1**	0.0585509	1.00252
**JSRP1**	0.0696412	1.00252
**5930400O16RIK**	0.0197656	1.00251
**D030040J03RIK**	0.0253118	1.00251
**PIGY**	0.0493914	1.00251
**9630020E24RIK**	0.0806815	1.00251
**PCDHA2**	0.000870571	1.0025
**WFDC13**	0.0586405	1.0025
**NECL1-PENDING**	0.0646007	1.0025
**ATAD5**	0.0708734	1.0025
**ISOC1**	0.0778367	1.0025
**A230068D05RIK**	0.0820264	1.0025
**LOC434960**	0.00382465	1.00249
**E030004P15RIK**	0.0140395	1.00249
**LIMS1**	0.0184154	1.00249
**COLQ**	0.0139315	1.00248
**ARSI**	0.0173697	1.00248
**SF3B1**	0.0181399	1.00248
**TC10L-PENDING**	0.0520194	1.00248
**LOC639558**	0.0754088	1.00248
**PTDSS1**	0.0778954	1.00248
**TACR3**	0.0063067	1.00247
**TRPM8**	0.0104032	1.00247
**LOC268939**	0.0269362	1.00247
**A830091E24RIK**	0.0462256	1.00247
**LOC383033**	0.053612	1.00247
**OLFR1259**	0.0729347	1.00247
**6330579M01RIK**	0.0760145	1.00247
**9627521_3631_RC**	0.0776476	1.00247
**BCL6B**	0.00730462	1.00246
**1700008P02RIK**	0.0232497	1.00246
**SPIN4**	0.0316847	1.00246
**CD209A**	0.022263	1.00245
**2810003K23RIK**	0.0564301	1.00245
**HAMP2**	0.0598869	1.00245
**A130002I06RIK**	0.0614313	1.00245
**NLRP4A**	0.0634848	1.00245
**1200011M11RIK**	0.0534724	1.00244
**TGDS**	0.0653744	1.00244
**LOC238599**	0.0172725	1.00243
**MAPKSP1**	0.0392559	1.00243
**LOC382243**	0.056789	1.00243
**2400002C23RIK**	0.0595725	1.00243
**LOC100040384**	0.0660379	1.00243
**LOC100045176**	0.078765	1.00243
**C430048L16RIK**	0.00272106	1.00242
**ZBED3**	0.0069992	1.00242
**EG629678**	0.0469283	1.00242
**C530024P05RIK**	0.0518613	1.00242
**AA987161**	0.0824663	1.00242
**B830017H08RIK**	0.00569506	1.00241
**TPX2**	0.0519286	1.00241
**9130204K15RIK**	0.0112537	1.0024
**PNMA5**	0.0461401	1.0024

C230081H03RIK	0.0521138	1.0024
6720467J09RIK	0.0574617	1.0024
LOC230602	0.0646909	1.0024
LONRF3	0.0688536	1.00239
LOC383460	0.0802795	1.00239
6030490I01RIK	0.0495916	1.00238
6720402K17RIK	0.0528149	1.00238
4921504I02RIK	0.0652999	1.00238
APOL2	0.0662711	1.00238
LOC672946	0.0809725	1.00238
BUB1	0.00215764	1.00237
LOC100045795	0.00151204	1.00237
2410127L17RIK	0.084093	1.00237
5730466H23RIK	0.065583	1.00236
CPNE3	0.0689064	1.00236
IKZF3	0.0774696	1.00236
BC053393	0.0244956	1.00235
DHRS13	0.0532759	1.00235
LOC385653	0.0609501	1.00235
CMKBR2	0.0644026	1.00235
E130317O18RIK	0.00868023	1.00234
EG545963	0.00866591	1.00234
MINA	0.0112931	1.00234
ZFYVE28	0.0346863	1.00234
OPN1SW	0.0155358	1.00233
OLFR765	0.0567828	1.00233
2310061I04RIK	0.0590777	1.00233
UTP18	0.079415	1.00233
OLFR1156	0.0801182	1.00233
LOC209112	0.0188407	1.00232
LOC386513	0.0108507	1.00231
LOC383541	0.0116799	1.00231
LOC383674	0.0321224	1.00231
ZFP346	0.0348202	1.00231
SCL0001951.1_12	0.000328181	1.0023
4933414E15RIK	0.0279069	1.0023
MAP3K5	0.0497664	1.0023
A330072G01RIK	0.0545621	1.0023
LOC574405	0.0405966	1.00229
5330425C20RIK	0.065221	1.00229
4933413B09RIK	0.0713877	1.00229
MAPK7	0.0302427	1.00228
E130119H03RIK	0.0442549	1.00228
2410018I08RIK	0.0617566	1.00228
B830017A01RIK	0.0635837	1.00228
OLFR635	0.0127489	1.00227
LOC545952	0.0300769	1.00227
UBXN11	0.0410629	1.00227
8430437N05RIK	0.0429951	1.00226
EG546250	0.0484532	1.00226
1700042B14RIK	0.0431182	1.00225
LOC232974	0.0835345	1.00225
A730020L20RIK	0.00818594	1.00224
ADH6A	0.0708073	1.00224
TLR12	0.00215026	1.00223
CHRDL2	0.0210908	1.00223
METTL7A1	0.0516319	1.00223
3110040M04RIK	0.0731397	1.00223

**OLFR575**	0.0844491	1.00223
**PRDM16**	0.00555665	1.00222
**LOC100041576**	0.0608032	1.00222
**V1RB10**	0.000382378	1.00221
**F830036K18RIK**	0.0312987	1.0022
**ARHGAP5**	0.0146415	1.00219
**D630033O11RIK**	0.0399921	1.00219
**A630026F04**	0.0523959	1.00219
**NUDCD1**	0.0606752	1.00219
**LOC100047028**	0.0823885	1.00219
**SLC14A2**	0.0080513	1.00218
**LOC666363**	0.0108743	1.00218
**MTERFD3**	0.0221278	1.00218
**BB075781**	0.0396334	1.00218
**SCL000896.1_70**	0.0516299	1.00218
**LASS6**	0.00618366	1.00217
**LOC100040159**	0.00343279	1.00217
**2610303G11RIK**	0.0172476	1.00217
**AKR1C14**	0.0244727	1.00216
**OLFR508**	0.0281122	1.00216
**LOC240089**	0.0405498	1.00216
**RASSF8**	0.0165109	1.00215
**4932411G08RIK**	0.042602	1.00214
**B930075B08RIK**	0.0670581	1.00214
**DSCAML1**	0.0263223	1.00213
**KCNJ14**	0.0808914	1.00212
**NWD1**	0.0263726	1.00211
**LOC384830**	0.0620699	1.00211
**EG631624**	0.0825467	1.00211
**LOC236053**	0.000375109	1.0021
**AGR3**	0.0141936	1.0021
**A830021K08RIK**	0.0671358	1.0021
**LOC384162**	0.00420933	1.00209
**IGK-V1**	0.0806074	1.00209
**1700030F18RIK**	0.0559028	1.00208
**4930579N16RIK**	0.0323074	1.00207
**SYNJ1**	0.0375116	1.00207
**A530031I11RIK**	0.0371552	1.00206
**1700024B05RIK**	0.0472513	1.00206
**PLCL2**	0.0486477	1.00206
**B020031M17RIK**	0.0744004	1.00206
**LOC383443**	0.0780673	1.00206
**CCDC102A**	0.0633745	1.00205
**MSX2**	0.0192936	1.00204
**B230397F11RIK**	0.00598047	1.00203
**CLDN22**	0.0326256	1.00202
**OLFR30**	0.0580729	1.00202
**C630017J20RIK**	0.0303082	1.00201
**LOC384322**	0.0410013	1.00201
**KCND1**	0.0587977	1.002
**LOC385089**	0.0134921	1.00199
**ZFP157**	0.0210781	1.00199
**4933440M02RIK**	0.040662	1.00199
**2810401C16RIK**	0.0103947	1.00198
**PRL6A1**	0.0450994	1.00198
**2610510H03RIK**	0.0662967	1.00198
**2410095B20RIK**	0.04982	1.00197
**A530079E22RIK**	0.0663451	1.00197

**JMJD4**	0.0796613	1.00197
**D130079A10RIK**	0.0122907	1.00196
**LOC383428**	0.0134702	1.00196
**LOC214456**	0.0194873	1.00195
**LOC381014**	0.0380549	1.00195
**TAS2R137**	0.0657714	1.00194
**AMBP**	0.0509287	1.00193
**RGN**	0.0786722	1.00193
**SLC10A2**	0.0814355	1.00193
**DHRS7C**	0.0167898	1.00192
**NCF2**	0.029038	1.00192
**HFE2**	0.0504078	1.00192
**OLFR207**	0.0216088	1.00191
**LOC385904**	0.0235851	1.00191
**ABPZ**	0.036139	1.00191
**OTX3**	0.0365912	1.00191
**OLFR744**	0.0485106	1.00191
**IGHV1S57_D13200_IG_HEAVY_VARIABLE_1S57_245**	0.0364933	1.0019
**4930447A16RIK**	0.0602531	1.0019
**TTC32**	0.0804591	1.0019
**UROC1**	0.0391628	1.00189
**4930521I23RIK**	0.0525756	1.00189
**GPR111**	0.0034103	1.00188
**ART1**	0.035185	1.00188
**PPT2**	0.0844177	1.00188
**SCL0002978.1_3**	0.0753743	1.00187
**OLFR98**	0.0254551	1.00186
**4933406B17RIK**	0.0257338	1.00185
**6430526O11RIK**	0.0455409	1.00185
**LOC630242**	0.0786652	1.00185
**4930564O18RIK**	0.0243655	1.00184
**LOC385200**	0.0397662	1.00184
**OLFR1179**	0.0399056	1.00184
**GRAP**	0.0637506	1.00184
**SCL0002292.1_30**	0.0669792	1.00183
**D930020B18RIK**	0.0807148	1.00183
**GM1968**	0.0595181	1.00182
**NEU4**	0.074002	1.00182
**IL18BP**	0.0119496	1.00181
**LOC277139**	0.0199163	1.00181
**SPOCK1**	0.0223282	1.00181
**ADA**	0.0417173	1.00181
**FOXO4**	0.0681219	1.0018
**LOC380925**	0.0501155	1.00179
**CAP2**	0.0216486	1.00178
**OLFR582**	0.0302327	1.00178
**EG381070**	0.0710572	1.00178
**SCL0002657.1_5**	0.0813222	1.00178
**OLFR527**	0.0184579	1.00177
**OPALIN**	0.0277819	1.00177
**MARCH1**	0.0461951	1.00177
**FGA**	0.0517221	1.00177
**D130002C14RIK**	0.0616836	1.00177
**IL8RB**	0.0448789	1.00176
**B230112C05RIK**	0.000141379	1.00175
**9530084K20RIK**	0.0583435	1.00175
**LOC230461**	0.0217334	1.00174
**LOC624071**	0.0632104	1.00174

**LOC640196**	0.0661032	1.00174
**WFDC15B**	0.025543	1.00173
**EG664876**	0.00587434	1.00172
**OLFR1107**	0.0591003	1.00172
**SKAP1**	0.0362839	1.00171
**ASB4**	0.0468018	1.0017
**LOC386018**	0.0525029	1.00169
**NEDD4B**	0.0673986	1.00169
**OLFR1287**	0.0688032	1.00169
**D330017J20RIK**	0.0814812	1.00169
**ITPA**	0.0826802	1.00169
**1200017F15RIK**	0.0831988	1.00169
**OLFR1105**	0.0107253	1.00168
**ARRDC2**	0.012965	1.00168
**9830167H18RIK**	0.0552865	1.00168
**GHRHR**	0.064797	1.00168
**LOC381515**	0.0284065	1.00167
**D830015G02RIK**	0.0564464	1.00167
**OLFR599**	0.0775798	1.00166
**1600020E01RIK**	0.0253454	1.00164
**KRT80**	0.0745174	1.00163
**LOC384491**	0.0333902	1.00162
**LOC381647**	0.0442851	1.00161
**LOC215253**	0.0609674	1.00161
**OLFR498**	0.0786031	1.0016
**A730098N08RIK**	0.0452456	1.00159
**END3**	0.0703077	1.00159
**SPATA7**	0.0439484	1.00158
**1810046K07RIK**	0.000704605	1.00157
**LOC384681**	0.0392451	1.00157
**LOC384659**	0.00347147	1.00156
**RNASE12**	0.0724107	1.00155
**PEX5L**	0.0615644	1.00154
**4921530G03RIK**	0.077569	1.00154
**9330119M13RIK**	0.0482996	1.00153
**ZFP324**	0.0704037	1.00153
**LOC100040701**	0.0137636	1.00152
**GM1070**	0.0402281	1.00152
**LOC546629**	0.061499	1.00152
**ELOVL3**	0.0658992	1.00152
**D130059C03RIK**	0.0691179	1.00152
**TLR11**	0.00120731	1.00151
**NBN**	0.0409114	1.00151
**FUCA2**	0.0673969	1.00151
**LOC381645**	0.0672556	1.0015
**GM1698**	0.0774949	1.0015
**OLFR1186**	0.0246791	1.00148
**E330018D23RIK**	0.046862	1.00148
**4930415F15RIK**	0.0685325	1.00148
**D030065J16RIK**	0.0460859	1.00147
**ITGP**	0.0610164	1.00147
**LOC100048579**	0.0453494	1.00146
**1700065I17RIK**	0.0576854	1.00145
**HEPHL1**	0.0481121	1.00144
**MORN5**	0.0487381	1.00144
**4932418E24RIK**	0.0775168	1.00144
**DIAP3**	0.0518247	1.00143
**1700039E15RIK**	0.0836751	1.00143

**B230333P14RIK**	0.0197587	1.00142
**ITIH5**	0.0108668	1.00141
**SLC27A5**	0.0748009	1.0014
**A830035I23RIK**	0.0195353	1.00139
**LOC213014**	0.0189999	1.00138
**LOC384839**	0.0241915	1.00138
**MGC60742**	0.0493045	1.00138
**LOC383067**	0.0164791	1.00137
**LOC675815**	0.0545299	1.00137
**4933404G15RIK**	0.0741884	1.00137
**PSG26**	0.0247074	1.00134
**MAST1**	0.0380787	1.00134
**DEAR1**	0.0605591	1.00134
**AI427809**	0.0485321	1.00133
**1700108K13RIK**	0.0289571	1.00132
**LIN28B**	0.0465485	1.00131
**SIT1**	0.0674802	1.00131
**AFP**	0.0778517	1.0013
**LOC381267**	0.0719723	1.00129
**LOC230970**	0.057785	1.00128
**LZTFL1**	0.0128845	1.00127
**RNASE13**	0.0172886	1.00126
**GLRA4**	0.0688642	1.00123
**NAT8L**	0.0283944	1.00122
**2310051M13RIK**	0.0659494	1.00122
**RAB3IL1**	0.070969	1.00122
**LOC381455**	0.0541142	1.00121
**OLFR738**	0.0124232	1.0012
**A930005K01RIK**	0.0314632	1.0012
**F830010A05RIK**	0.0431003	1.00118
**L1CAM**	0.0042796	1.00117
**LOC331012**	0.00185629	1.00115
**6720485J18RIK**	0.0113107	1.00113
**9430050L06RIK**	0.0210783	1.00111
**GALNT9**	0.037699	1.00111
**E330019L22RIK**	0.0389037	1.00111
**E330027M22RIK**	0.0576588	1.00111
**2810047L02RIK**	0.0612897	1.00111
**LRP2**	0.00550401	1.00107
**ENTPD7**	0.0744101	1.00107
**KLF15**	0.0603859	1.00106
**TDPOZ3**	0.0615689	1.00106
**SEC15L1**	0.00145612	1.00105
**V1RD7**	0.0820498	1.00105
**LOC633661**	0.0263417	1.00104
**CREB1**	0.0450144	1.00104
**E430021E22RIK**	0.0674789	1.00104
**1600012F09RIK**	0.0549582	1.001
**LOC668779**	0.0639514	1.001
**LOC385292**	0.0666582	1.001
**1110034B05RIK**	0.0285768	1.00099
**CUBN**	0.0821258	1.00098
**SGIP1**	0.0350527	1.00097
**E030042M04RIK**	0.0468183	1.00092
**SLC7A2**	0.0232309	1.0009
**PIRA11**	0.0183925	1.00089
**A930038B10RIK**	0.0349019	1.00089
**SPATC1**	0.077223	1.00089

**PROP1**	0.0408963	1.00081
**RHOX11**	0.0698322	1.00079
**LOC381842**	0.002409	1.00072
**4930419B13RIK**	0.0744074	1.00072
**A430110O13RIK**	0.0283744	1.00071
**GPR152**	0.076282	1.00069
**ATOH8**	0.053088	1.00068
**HOXA9**	0.0691215	1.00065
**IPO7**	0.00931289	1.00061
**CNTNAP5C**	0.0561378	1.00055
**KREMEN-PENDING**	0.015939	1.00052
**RASL10A**	0.00624705	1.00051
**LOC385279**	0.0488531	1.00051
**LOC386560**	0.0628404	1.00051
**LOC384190**	0.0379325	1.00049
**A930029B02RIK**	0.0142421	1.00043
**COL22A1**	0.0108249	1.00038
**1700030E15RIK**	0.0653368	1.00038
SNW1	0.0623767	-1.00028
RHOX10	0.0765903	-1.00033
PDE1A	0.0598029	-1.00037
LOC627081	0.0608006	-1.00042
FRMD3	0.0790623	-1.00052
D030015B04RIK	0.0374146	-1.00059
RGP1	0.0552435	-1.00062
BC052688	0.070162	-1.0007
SGSM1	0.0115749	-1.00073
C76746	0.0318442	-1.00074
LOC100044292	0.0224022	-1.00078
A030010B05RIK	0.0386166	-1.0008
SCL0003955.1_3	0.0748365	-1.00081
BC016495	0.0761663	-1.00087
LOC385883	0.0682818	-1.00088
PHKA1	0.0660321	-1.00098
PPEF2	0.0617646	-1.00099
PNPT1	0.00224929	-1.00101
CNTF	0.0285575	-1.00101
LOC100047833	0.0568459	-1.00106
H2-Q1	0.0538218	-1.00111
4921538N17RIK	0.0843251	-1.00111
1110037J23RIK	0.052834	-1.00119
5430411J08RIK	0.0835647	-1.00123
9629812_535	0.084253	-1.00123
4930406P12RIK	0.073905	-1.00125
CRH	0.0196159	-1.00126
4933422H20RIK	0.0280174	-1.00129
MIZF	0.0653419	-1.00132
GSDMA3	0.0224894	-1.00137
COX7A1	0.0630176	-1.00141
LOC382192	0.0584037	-1.00146
LOC100040328	0.0148889	-1.0015
PTPN3	0.0280771	-1.00152
CEL	0.0168502	-1.00154
INDOL1	0.0133737	-1.00156
4930518C23RIK	0.0206616	-1.00156
A530012E19RIK	0.00375293	-1.00158
TCERG1L	0.026998	-1.00158
KERA	0.0525957	-1.00158

ZFP622	0.0810358	-1.00159
2310047M15RIK	0.0520385	-1.0016
A730027E01RIK	0.0658517	-1.00162
PTGDR	0.0146057	-1.00163
PRH1	0.0472379	-1.00164
NLRP9A	0.0205701	-1.00165
RIMS3	0.0366441	-1.00168
G630052O08RIK	0.0453283	-1.00173
IFI204	0.0567854	-1.00173
LOC100041004	0.0335521	-1.00174
RFK	0.0432338	-1.00174
ZDHHC11	0.057448	-1.00181
CAR14	0.0812771	-1.00181
CYP2B13	0.00182142	-1.00182
DNM	0.0296006	-1.00183
GPRIN2	0.0635644	-1.00184
E130208E03RIK	0.017247	-1.00187
SCL0004025.1_69	0.018761	-1.00187
B930050E02RIK	0.0216181	-1.00189
2010309E21RIK	0.0371141	-1.0019
PIGN	0.0701049	-1.00197
U46068	0.0189419	-1.00198
DONSON	0.0348318	-1.002
GIYD2	0.0362636	-1.00201
1700123A16RIK	0.0492109	-1.00201
SLC6A7	0.0245851	-1.00202
CDKN2AIP	0.0233069	-1.00203
OLFR1028	0.0613231	-1.00205
SCL0001065.1_54	0.0282062	-1.00206
EG668225	0.0469332	-1.00207
TMEM91	0.0503948	-1.00207
DFFA	0.025785	-1.0021
CD69	0.0489509	-1.0021
COPS8	0.0709171	-1.0021
DPYSL5	0.0846452	-1.0021
LOC100046143	0.0660332	-1.00211
2310040G07RIK	0.0315785	-1.00213
KIF1C	0.0427098	-1.00215
B3GALT5	0.0569464	-1.00216
PPP1R1B	0.0196519	-1.00217
4930415J05RIK	0.0790308	-1.00217
SNAI1	0.0723436	-1.00219
GPR65	0.0336277	-1.00221
LOC671947	0.0533954	-1.00222
LOC237036	0.0140262	-1.00225
LOC232867	0.017327	-1.00226
1700021K10RIK	0.0678401	-1.00228
2210008F06RIK	0.0472159	-1.00229
SCL0001533.1_63	0.0474199	-1.0023
KCNK7	0.0594473	-1.00233
GYG	0.0425249	-1.00234
IL3	0.0729855	-1.00236
2410170E21RIK	0.0408102	-1.00238
PIF1	0.0491158	-1.00239
SAA2	0.0158293	-1.0024
HK3	0.0797608	-1.00244
AGTR1B	0.0836302	-1.00245
LOC100044622	0.028787	-1.00246

CMYA5	0.053989	-1.00246
IQCF3	0.0682596	-1.00247
FIGNL1	0.0317048	-1.0025
LOC666238	0.0826006	-1.00252
3110035E14RIK	0.0155339	-1.00253
EHD2	0.0581141	-1.00253
CAMKK1	0.0774096	-1.00254
UBE2T	0.00940231	-1.00255
DSCR6	0.052738	-1.00256
3830417A13RIK	0.0192539	-1.00257
RASL11A	0.0638924	-1.00257
IGF1	0.0344616	-1.0026
OLFR62	0.0145568	-1.00262
EPOR	0.0198604	-1.00262
OLFR69	0.0701869	-1.00267
LOC385510	0.0136444	-1.00268
CORO2A	0.0769585	-1.00268
OLFR419	0.0186551	-1.00269
LIFR	0.0800355	-1.00269
1700101C01RIK	0.0016326	-1.0027
3010026O09RIK	0.0406478	-1.00272
PRX	0.0492324	-1.00273
RAB26	0.0728712	-1.00273
SIPA1	0.0828029	-1.00274
RASEF	0.0678255	-1.00276
TULP2	0.0695096	-1.00276
ABHD1	0.0108938	-1.00278
IL28B	0.0345608	-1.00278
BHMT2	0.0784471	-1.0028
A430090E18RIK	0.0581724	-1.00283
CRF-R 2	0.0739322	-1.00283
KMO	0.0432445	-1.00288
2410091C18RIK	0.0362637	-1.00296
AI118078	0.0721118	-1.00296
ZFP93	0.0142116	-1.00302
UROS	0.0724687	-1.00302
LIMD2	0.0375298	-1.00305
TMEM90A	0.0761661	-1.00305
TMEM25	0.0541001	-1.00306
CPT1C	0.03142	-1.00307
LHX2	0.0422517	-1.00309
COL5A2	0.034482	-1.00312
EAR4	0.0544469	-1.00318
FANCB	0.0774777	-1.00318
DIAP1	0.0836035	-1.00319
BTRC	0.0350831	-1.00323
D330038K10RIK	0.00851156	-1.00324
LRP11	0.0227904	-1.00326
TROAP	0.0305575	-1.00326
DPY19L4	0.048384	-1.00328
SCL0001912.1_11	0.0372051	-1.00331
BRUNOL4	0.0728682	-1.00332
TBC1D9B	0.0190188	-1.00344
GPR124	0.0572007	-1.00345
POLM	0.0618966	-1.00345
1700013F07RIK	0.0637359	-1.00346
GSDMA1	0.0380512	-1.00348
CD40	0.0653881	-1.0035

5330421K23RIK	0.0543761	-1.00351
EG633285	0.00540971	-1.00354
SLC22A17	0.0469799	-1.00355
LOC382081	0.069297	-1.00355
A230074L19RIK	0.00109633	-1.00358
FAAH	0.0657661	-1.00358
2410015C20RIK	0.032211	-1.0036
GZMK	0.0676124	-1.0036
BCORL1	0.00622623	-1.00361
LOC433632	0.0400874	-1.00363
VAMP1	0.0234327	-1.00364
B230380O10RIK	0.0376361	-1.00364
TMPRSS6	0.0100547	-1.00366
2810021O14RIK	0.044996	-1.00366
AI314976	0.0840341	-1.00367
2310033E01RIK	0.0627853	-1.00369
PGM2L1	0.077006	-1.00372
PFKFB2	0.0209589	-1.00382
PATZ1	0.0335604	-1.00383
TACR1	0.0593122	-1.00383
4631416I11RIK	0.0133556	-1.00384
A330080J22RIK	0.0211025	-1.00384
RGS2	0.0400411	-1.00384
UHRF1	0.00543776	-1.00386
LOC385181	0.0841623	-1.00387
GRIA3	0.0335991	-1.00389
UBE2I	0.0385269	-1.0039
4632412I06RIK	0.0579832	-1.0039
OLFR884	0.0252973	-1.00395
ZFP2	0.000410306	-1.00397
SCL000528.1_16	0.0559072	-1.00397
NLRP4F	0.00951122	-1.00399
CEP55	0.0658322	-1.004
2210016F16RIK	0.0429925	-1.00404
D11MOH35	0.072346	-1.00404
EHBP1	0.00767278	-1.00409
B930095G15RIK	0.0665415	-1.0041
MSH2	0.0282744	-1.00411
ATP6V1G2	0.00288787	-1.00412
ZFP687	0.0177721	-1.00412
TM6SF1	0.0030104	-1.00414
WFDC5	0.0440692	-1.00418
NMI	0.0453977	-1.0042
SIGLEC1	0.0825011	-1.00421
CLIP3	0.019813	-1.00423
5033413D22RIK	0.0586219	-1.00423
NLN	0.0145824	-1.00424
MCPH1	0.014332	-1.00427
PODXL2	0.0150544	-1.00429
C530028I08RIK	0.0253701	-1.00429
GVIN1	0.0790088	-1.00429
LOC385924	0.0849948	-1.00429
RWDD3	0.051419	-1.00432
2610029J22RIK	0.0632126	-1.00432
NOD2	0.0628097	-1.00433
HADH	0.0751766	-1.00434
NRAP	0.0810172	-1.00435
RAD9	0.045742	-1.0044

LOC100044159	0.0848706	-1.00446
2410006F04RIK	0.0473073	-1.00447
BLOC1S3	0.0480845	-1.00448
FCRLS	0.00108504	-1.00449
FBLN7	0.0556283	-1.00451
RBM14	0.0585867	-1.00451
DOK2	0.00370521	-1.00452
EVI2A	0.0194381	-1.00453
MPG	0.082975	-1.00454
3830430K15RIK	0.0147839	-1.00455
NEK3	0.0239874	-1.00455
TSGA14	0.0208892	-1.00457
SLC28A1	0.0348946	-1.00459
DDEF2	0.0355586	-1.00462
USP30	0.0120784	-1.00463
A130010J15RIK	0.0602016	-1.00463
MLXIPL	0.0701703	-1.00467
ASL	0.00445767	-1.00471
4930444P10RIK	0.0799874	-1.00471
PRODH	0.0351523	-1.00473
LOC381578	0.0105624	-1.00474
TMEM44	0.0182426	-1.00474
CEBPA	0.0190412	-1.00474
ZDHHC16	0.0585235	-1.00475
FFAR3	0.0673144	-1.00477
REC8	0.0576699	-1.00481
LOC237408	0.0665865	-1.00484
NCOA4	0.0389777	-1.00485
MARE	0.0531262	-1.00486
PLSCR2	0.00925641	-1.00489
6430573D20RIK	0.0248	-1.0049
ASB6	0.0658259	-1.00492
TRIM68	0.00584322	-1.00494
C630044O20RIK	0.0134723	-1.00494
DPP9	0.0489475	-1.00494
LOC332300	0.0498712	-1.00494
MCTS2	0.0291896	-1.00496
TNFSF8	0.0597076	-1.00496
9830002I17RIK	0.0738461	-1.00497
RMND5B	0.0339619	-1.00498
NRXN3	0.0665742	-1.00498
4833417A11RIK	0.0117462	-1.00499
LOC277236	0.0673814	-1.00499
EVC	0.0306499	-1.005
EAR2	0.0383345	-1.005
IL4RA	0.0223625	-1.00501
6430514E13RIK	0.0397314	-1.00503
AIF1	0.0212502	-1.00507
GAL3ST1	0.0291347	-1.00508
TCFE2A	0.0127238	-1.00513
NXF7	0.0393786	-1.00513
ZFP429	0.0569567	-1.00513
1810043G02RIK	0.0843917	-1.00514
LOC100047339	0.00819254	-1.00516
ZC4H2	0.0221814	-1.00517
2810032E02RIK	0.0212948	-1.00518
LOC385849	0.00364505	-1.00519
KRT71	0.0709408	-1.00519

PPP4R4	0.028568	-1.00521
2410013I23RIK	0.0473882	-1.00521
CCR5	0.0338468	-1.00526
D930028F11RIK	0.0677413	-1.00526
SELPLG	0.0064493	-1.0053
MSLNL	0.0353496	-1.0053
SDSL	0.0663107	-1.0053
BC016201	0.0547854	-1.00531
LOC223653	0.0605503	-1.00534
CD86	0.0483666	-1.00535
TMEM132E	0.031178	-1.00538
INTS9	0.0429426	-1.00538
SLCO3A1	0.0151938	-1.00539
1200015F23RIK	0.0434091	-1.0054
MED22	0.00378528	-1.00544
ZFP235	0.00953325	-1.00547
PSMD5	0.0107133	-1.00547
MFAP4	0.0351705	-1.00547
G6PC3	0.0654842	-1.00548
CD97	0.0544319	-1.0055
CHD3	0.0107497	-1.00552
GGA1	0.0586497	-1.00555
4732423C17RIK	0.037923	-1.00556
3010015F07RIK	7.56E-05	-1.00558
NUDT13	0.0527344	-1.00558
ACRBP	0.0657771	-1.00559
6330401K01RIK	0.0340824	-1.0056
LOC211870	0.053169	-1.00561
4833409F13RIK	0.011365	-1.00564
CAMK2N2	0.0418512	-1.00565
PSMD9	0.0786826	-1.00565
TRIB1	0.0820336	-1.00569
CHST5	0.053225	-1.00571
INHA	0.00631626	-1.00573
9030405D14RIK	0.0830714	-1.00573
GNG13	0.0218948	-1.00575
A930039A15RIK	0.0193853	-1.00576
MYO1B	0.0212566	-1.00577
WFIKKN1	0.0396171	-1.00578
A730054J21RIK	0.00995751	-1.00582
PTPRU	0.0444238	-1.00582
LEFTY1	0.0841559	-1.00584
4930431J19RIK	0.0162783	-1.00586
EPB4.1L1	0.0770834	-1.00588
SCL000361.1_1	0.042639	-1.0059
MAPK12	0.0108788	-1.00593
RDH5	0.0833821	-1.00593
LOC100045971	0.0048475	-1.00595
TMEM189	0.0035448	-1.00597
MCOLN2	0.0812214	-1.006
FAM173B	0.008844	-1.00603
LOC100043313	0.010292	-1.00604
LPXN	0.00579448	-1.00605
CRSP3	0.00765288	-1.00609
A930001N09RIK	0.0216196	-1.00611
ISOC2A	0.0567389	-1.00611
DNALC1	0.0589139	-1.00611
ZFP386	0.000849385	-1.00613

WNT4	0.0105039	-1.00618
WDR47	0.0330999	-1.00618
USF2	0.0293562	-1.00621
EG406223	0.0097052	-1.00622
FBXO31	0.00823823	-1.00622
LOC385256	0.0478386	-1.00623
FAIM2	0.0519895	-1.00624
SGOL2	0.00469818	-1.00626
ABHD3	0.0571697	-1.00627
ACOX3	0.0473169	-1.00628
IDUA	0.0383564	-1.00632
B230349N02RIK	0.034607	-1.00633
C130032J12RIK	0.0403738	-1.00635
MBD4	0.0543584	-1.00638
NOSIP	0.0492363	-1.00639
CDC42BPG	0.033092	-1.00646
PANK1	0.0552966	-1.00646
SCNN1G	0.0110689	-1.0065
RAB19	0.0285442	-1.00651
LIG1	0.0646636	-1.00652
2410017P07RIK	0.01269	-1.00653
TUSC1	0.0798861	-1.00655
KIFC3	0.0571116	-1.00657
HTRA3	0.0619624	-1.00659
GAS2L1	0.0565273	-1.00662
UPK2	0.067739	-1.00663
METAPL1	0.00452707	-1.00664
DYNC2H1	0.0131378	-1.00668
LOC100045439	0.00179357	-1.00675
LOC383561	0.0818662	-1.00675
ACAD10	0.0275404	-1.00676
EXOSC6	0.0370814	-1.00676
LOC100044774	0.0846993	-1.00678
SCRN2	2.35E-05	-1.00681
LOC100047603	0.0219977	-1.00681
KANK3	0.0111675	-1.00682
XRCC1	0.0187961	-1.00682
DUS3L	0.0560372	-1.00685
VPS26A	0.0317952	-1.00687
1600012H06RIK	0.0490748	-1.00687
SDCCAG3	0.0795278	-1.00688
APIP	0.0608627	-1.00689
SLC47A1	0.0652774	-1.00689
C1QTNF1	0.0386241	-1.00692
A630064P09RIK	0.0723203	-1.00692
BC024868	0.0704379	-1.00693
1700024G10RIK	0.0124293	-1.00695
SLC2A13	0.00466454	-1.00697
DTX4	0.0777624	-1.00697
GREB1	0.00845036	-1.00701
LRSAM1	0.0162467	-1.00701
MRPL49	0.0167659	-1.00703
TLCD2	0.0172901	-1.00703
1700009P17RIK	0.0533772	-1.00705
CDC42EP5	0.0823912	-1.00707
CCDC125	0.0128102	-1.00708
SEMA4G	0.0361556	-1.00709
6430598A04RIK	0.0264559	-1.0071

EG381438	0.00463489	-1.00711
PPM1M	0.0160327	-1.00711
SLC8A1	0.0450467	-1.00711
5830457O10RIK	0.069929	-1.00714
AXIN2	0.0199486	-1.00718
5730403M16RIK	0.0073737	-1.00727
TMCO3	0.00734753	-1.00733
ADARB1	0.0584929	-1.00735
PIK3AP1	0.0256613	-1.00739
H1FX	0.0768405	-1.00743
MAPK11	0.0339714	-1.00745
SH3GL2	0.00508497	-1.0075
SCL0003251.1_21	0.0156011	-1.0075
LY6G6D	0.0302654	-1.00752
SLC35E4	0.0386075	-1.00759
TMEM46	0.0284116	-1.00762
LOC385822	0.0468056	-1.00763
3830406C13RIK	0.0419265	-1.00765
PROC	0.0310708	-1.00771
GLUL	0.0819956	-1.00771
1110032E23RIK	0.0361533	-1.00773
FBXO8	0.0388057	-1.00773
GBP5	0.015175	-1.00774
CCDC84	0.0228101	-1.00774
1300018I17RIK	0.0304534	-1.00774
LOC629763	0.0730944	-1.00777
KLHDC5	0.0367233	-1.00785
PCOLCE2	0.0430541	-1.00787
FAM110B	0.00254499	-1.00788
6330407I18RIK	0.0219325	-1.0079
9430015G10RIK	0.00972834	-1.00794
TMEM166	0.0749314	-1.00797
FMO1	0.0555716	-1.00799
NCDN	0.0227054	-1.00801
SFI1	0.0319386	-1.00801
SEPT1	0.0370178	-1.00801
CPA3	0.0510712	-1.00801
2900093K20RIK	0.0794055	-1.00802
LOC100047915	0.0283359	-1.00808
RHOBTB2	0.0102445	-1.00809
2510002D24RIK	0.0391508	-1.00815
TMTC4	0.0738975	-1.00818
1190008K20RIK	0.043151	-1.00822
LY86	0.0256974	-1.00827
VPS41	0.0271802	-1.00828
GHDC	0.0132791	-1.0083
PFAS	0.012141	-1.00835
9430030N17RIK	0.0129392	-1.00835
2410004N11RIK	0.0396999	-1.00837
MFSD9	0.000204497	-1.00838
9430077D24RIK	0.0802481	-1.0084
IL18	0.013271	-1.00841
NGFR	0.0469854	-1.00842
OAS1B	0.0827207	-1.00842
UNC13D	0.0381549	-1.00843
GNA12	0.0806536	-1.00846
TMEM101	0.0841479	-1.00846
A930008G19RIK	0.0489143	-1.00849

KLHDC1	0.0559393	-1.00849
WNK4	0.00729891	-1.00851
CHRD	0.000948845	-1.00852
OSR2	0.016303	-1.00853
HOXC6	0.0111613	-1.00854
GNAZ	0.0242108	-1.00854
TLE2	0.0336811	-1.00855
SCL0002007.1_97	0.0604606	-1.00856
FUT10	0.0363889	-1.00858
MNS1	0.030907	-1.00859
2700046G09RIK	0.000541071	-1.0086
FCGR2B	0.010071	-1.0086
C130052O15RIK	0.0601624	-1.00861
CD83	0.00137811	-1.00866
RFC3	0.0659895	-1.00866
FGFR3	0.0195985	-1.00867
SULT2B1	0.0524356	-1.00867
SCL000205.1_10	0.0235275	-1.00868
HGFAC	0.045832	-1.00869
ATXN7L3	0.0406024	-1.00872
2210407N10RIK	0.0715055	-1.00872
AMDHD2	0.0767134	-1.00872
FRAG1	0.0417732	-1.00873
SENP7	0.0557891	-1.00873
LY6G6E	0.066342	-1.00875
DORZ1	0.043787	-1.00878
TSPAN1	0.0517669	-1.00878
FSD1	0.0102851	-1.00883
MGMT	0.0143925	-1.00886
UBXD7	0.0571094	-1.00891
GP49A	0.0102472	-1.00892
GIPC1	0.0135408	-1.00899
TACC1	0.0266933	-1.00901
CD53	0.0629515	-1.00901
4732473B16RIK	0.0445526	-1.00902
2610036L11RIK	0.0158633	-1.00903
ACSF3	0.018532	-1.00904
TUSC2	0.0441022	-1.00904
CTPS2	0.0422746	-1.00906
TAF6	0.0104991	-1.00907
MAGI1	0.0415505	-1.00908
SC5D	0.00623671	-1.00913
2310045A20RIK	0.021635	-1.00913
DTX1	0.00733672	-1.00918
PACSIN3	0.0482874	-1.00918
MRRF	0.0575332	-1.00919
3830402I07RIK	0.0791134	-1.00919
A530017D24RIK	0.0423559	-1.00922
B3GNT8	0.0769574	-1.00925
GGT1	0.0777809	-1.00925
ABCD1	0.0246762	-1.00926
U2AF1L4	0.0287279	-1.00928
ZFP821	0.0276017	-1.00932
1810005K13RIK	0.0336107	-1.00934
AMIGO1	0.0256999	-1.00937
NME4	0.00112794	-1.00939
2010001J22RIK	0.0588523	-1.00945
IL17RB	0.0367519	-1.00948

BCL11A	0.0205223	-1.00949
FAM110A	0.0215866	-1.00949
ZMIZ2	0.0806788	-1.00956
BC053749	0.0100012	-1.00957
DUSP28	0.0285893	-1.00962
ANTXR2	0.0465693	-1.00966
5730596K20RIK	0.0149828	-1.00968
FBF1	0.0651587	-1.00969
NUMBL	0.0383882	-1.0097
LOC665047	0.059788	-1.00971
DECR1	0.0171272	-1.00972
ZMAT5	0.0226471	-1.00974
CARM1	0.0442542	-1.00979
ALDH1L1	0.00675116	-1.0098
MRC1	0.0357495	-1.0098
6030495B01RIK	0.051838	-1.00982
EMILIN2	0.0839604	-1.00982
2210010N04RIK	0.0465497	-1.00984
C330018D20RIK	0.0431064	-1.00988
SPIRE2	0.048911	-1.0099
LRRC15	0.0776894	-1.00991
DBN1	0.0127045	-1.00993
PTDSS2	0.030653	-1.00993
C1QTNF4	0.0203419	-1.00998
2310009E04RIK	0.00259188	-1.01
BEX4	0.00582505	-1.01003
HSD17B11	0.0107736	-1.01005
TG737RPW	0.077176	-1.01005
LOC100046255	0.00124141	-1.0101
ITGA11	0.0531225	-1.01011
CLCN2	0.0654051	-1.01011
AP1S1	0.0813517	-1.01018
CHFR	0.0727206	-1.01026
E430002G05RIK	0.0200192	-1.01027
COVA1	0.0209963	-1.01027
ZBP1	0.000972362	-1.01029
LOC100046796	0.0717256	-1.01031
LOC100043918	0.0124328	-1.01032
CDK10	0.0640889	-1.01033
GM166	0.00724107	-1.01034
ITGA10	0.0404899	-1.01038
POLG	0.0543617	-1.01038
EFNB2	0.0633168	-1.0104
BCL2L1	0.0783978	-1.01041
TMEM121	0.0191418	-1.01043
TBC1D8	0.0156058	-1.01044
MBD5	0.0572075	-1.01044
BC018371	0.0220071	-1.01051
PPP4C	0.00994662	-1.01052
TAF11	0.047991	-1.01056
H2-DMA	0.0162065	-1.01057
CELSR1	0.0425664	-1.01058
2310005N01RIK	0.0294581	-1.01059
NGEF	0.0767438	-1.0106
CD70	0.017257	-1.01061
MYO5C	0.0378443	-1.01061
ZFP119	0.0638179	-1.01062
2510022D24RIK	0.0812491	-1.01063

LZIC	0.0425249	-1.01068
TMEM139	0.0458644	-1.01068
9030420J04RIK	0.0524879	-1.0107
ACAA1B	0.0321418	-1.01073
TCEAL3	0.0718467	-1.01073
DDX11	0.0297075	-1.01077
FASTKD1	0.0307998	-1.01079
RTN2	0.0617829	-1.01083
2810468N07RIK	0.0027565	-1.01084
STK36	0.000511176	-1.01089
FXYD2	0.0451511	-1.01089
AI464131	0.0437994	-1.0109
ATP6V0A1	0.0754412	-1.01093
CSF2RA	0.0580585	-1.01095
PEX16	0.0490865	-1.01097
ACN9	0.0732562	-1.011
FBXO36	0.0739506	-1.011
LOC100044257	0.0136856	-1.01101
ABAT	0.0265602	-1.01101
LOC100047937	0.0187894	-1.01108
4933439C10RIK	0.0106065	-1.01111
AP4M1	0.0442321	-1.01112
NEURL	0.0620845	-1.01114
FRAT2	0.0703299	-1.01116
SAT2	0.0399778	-1.01118
BAG4	0.0673171	-1.01118
2410187C16RIK	0.0187337	-1.0112
TOM1L1	0.00766484	-1.01122
HIST2H2BE	0.0295967	-1.01122
LOC384742	0.062324	-1.01123
2610036D13RIK	0.0495632	-1.01124
GCDH	0.00274417	-1.01125
BC026585	0.0293585	-1.01126
TBC1D5	0.0192544	-1.01134
C430049K18RIK	0.0106912	-1.01139
TMCO6	0.0333187	-1.01142
E230027K01RIK	0.00847906	-1.01147
NFKBIE	0.0043852	-1.01148
4930572J05RIK	0.0613021	-1.0115
SGSM3	0.0119143	-1.01154
RAB13	0.0533876	-1.01155
NQO2	0.00679495	-1.0116
TIRAP	0.00300945	-1.0116
SCL0004175.1_57	0.0518888	-1.0116
RASD1	0.0676027	-1.01161
LOC381150	0.0287179	-1.01162
LOC380947	0.00551855	-1.01163
TTYH2	0.0810621	-1.01165
RNF215	0.059178	-1.01167
PHTF1	0.0111389	-1.01168
GLIPR1	0.0557596	-1.01168
CMA2	0.0763823	-1.01169
RARG	0.0324013	-1.0117
B930045J24RIK	0.00130684	-1.01173
SRPX	0.043492	-1.01174
FCHSD1	0.0448989	-1.01176
VASP	0.00478341	-1.01177
LOC237459	0.041644	-1.0118

GTSE1	0.040403	-1.01182
OSM	0.057857	-1.01184
2900024C23RIK	0.0352817	-1.01189
NTHL1	0.0278326	-1.0119
B930004C15RIK	0.0699705	-1.01191
LOC383100	0.0365538	-1.01192
TBC1D7	0.00734334	-1.01194
NCKAP1L	0.0198928	-1.01195
ABHD8	0.0270497	-1.01195
SEPHS2	0.0828257	-1.012
SCL0002297.1_21	0.0521988	-1.01201
LST1	0.0499484	-1.01204
BMP3	0.0835603	-1.01204
SLC25A14	0.0116885	-1.01205
POLI	0.0702754	-1.0121
ABI3BP	0.0384024	-1.01211
DYNC1LI2	0.041525	-1.01211
1700054E11RIK	0.0460244	-1.01213
BC024561	0.0640622	-1.01213
LIME1	0.0792932	-1.01213
FGF13	0.0514224	-1.01214
FAM122B	0.0518406	-1.01219
RASSF6	0.000318917	-1.01223
CLEC4N	0.000731188	-1.01224
SPNB1	0.0814565	-1.01224
DOCK1	0.0347249	-1.01228
BC044804	0.0641638	-1.01229
MCF2L	0.0499996	-1.01233
ZFP771	0.0701774	-1.01233
IFT88	0.0530896	-1.01234
ICT1	0.0640621	-1.01235
D11LGP2E	0.0115901	-1.01236
SFRS16	0.0337088	-1.01237
9030607L17RIK	0.0540259	-1.01241
TOX	0.0776078	-1.01241
CMA1	0.00928649	-1.01244
OTTMUSG00000001070	0.00933236	-1.01244
AKR1C12	0.0670177	-1.01244
ATHL1	0.0527245	-1.01247
B230107H12RIK	0.0132033	-1.01248
IDS	0.0559161	-1.01253
HDAC10	0.0234886	-1.01254
IPP	0.0196219	-1.01256
PAIP1	0.069202	-1.01269
CDKL2	0.0429192	-1.01273
IKBKE	0.0815742	-1.01278
CXX1A	0.0307814	-1.0128
SLC29A4	0.0259995	-1.01283
PRTN3	0.0667024	-1.01284
B4GALT6	0.0731655	-1.01286
1700071K01RIK	0.0219301	-1.01287
LOC100044576	0.0437057	-1.01287
9130404D08RIK	0.0107694	-1.01288
WDSUB1	0.0812727	-1.01288
SYRADB	0.0819791	-1.01288
NUP133	0.0116386	-1.01289
PIK3R3	0.00180616	-1.01293
HERPUD2	0.0595367	-1.01295

PKIG	0.00657028	-1.01298
SLC4A3	0.0155771	-1.01301
MEA1	0.0675563	-1.01301
KLC1	0.0166912	-1.01303
EG620143	0.0736396	-1.01306
TSHZ2	0.00932092	-1.01309
LAYN	0.0468768	-1.01318
LOC100048436	0.00591551	-1.0132
VILL	0.0300661	-1.01321
GINS2	0.0278011	-1.01323
LOC669001	0.0541225	-1.01324
STAC	0.02862	-1.01327
HCR	0.0413445	-1.01329
ETV6	0.0787892	-1.01329
COMMD9	0.0543208	-1.01332
GLB1	0.0179377	-1.01333
ADAMTS12	0.00446756	-1.01339
ZFP521	0.023455	-1.01341
DNASE1L1	0.0316855	-1.01342
MAPK3	0.0747636	-1.01346
SBNO2	0.0531874	-1.01348
TMEM107	0.0322202	-1.0135
ATL3	0.013255	-1.01356
OGN	0.0467484	-1.01361
TMEM158	0.0829521	-1.01361
1700007P14RIK	0.0560106	-1.01362
NEO1	0.0221694	-1.01363
FAHD2A	0.00317534	-1.01364
ETV1	0.00789581	-1.01368
ALDH3B1	0.0153508	-1.01368
BC023744	0.00984464	-1.01372
NDUFB2	0.0376325	-1.01374
SLC25A10	0.0137634	-1.01376
TMEM117	0.064729	-1.01376
CGN	0.0806414	-1.01387
1700065O13RIK	0.0643379	-1.01388
INPP5E	0.0669261	-1.01389
COPG2	0.018437	-1.0139
EG624138	0.0201679	-1.01393
TMEM141	0.00490613	-1.01394
EG329521	0.0513062	-1.01395
RAC3	0.01116	-1.01401
UGT1A6A	0.0132303	-1.01404
MAP4K2	0.0458454	-1.01406
KREMEN1	0.0422038	-1.01408
PTPN12	0.00855202	-1.01409
GTRGEO22	0.0344282	-1.01415
APOBEC3	0.0220411	-1.01416
D4WSU114E	0.0500785	-1.01417
ARRB2	0.0317582	-1.01418
LTBR	0.0557738	-1.01418
NSMAF	0.00282077	-1.01419
CXX1C	0.0591013	-1.01424
LOC619973	0.0757043	-1.01424
BAHD1	0.0256984	-1.01425
TLR4	0.0622033	-1.01425
9530081N05RIK	0.076632	-1.01426
H2-DMB2	0.0652212	-1.01432

UCN2	0.020915	-1.01435
SNAI3	0.0182185	-1.01437
SP140	0.0109908	-1.01443
PIK3R2	0.0502412	-1.01444
2900006A08RIK	0.0222098	-1.01453
TWIST2	0.070795	-1.01459
SORD	0.00332965	-1.01461
PPS	0.0081687	-1.01464
4732456N10RIK	0.0216289	-1.01474
1190002F15RIK	0.0107003	-1.01475
1110059E24RIK	0.0479784	-1.01476
ADH5	0.0228036	-1.01486
CCDC114	0.0143688	-1.01487
1110067B18RIK	0.0434421	-1.01487
ACSS2	0.0722633	-1.01488
TFPT	0.0743532	-1.0149
CAR11	0.0768206	-1.01492
RAP2A	0.0012407	-1.01494
THG1L	0.0547374	-1.01495
ARFRP1	0.0485309	-1.01496
ADCY7	0.0678165	-1.01502
C030014I23RIK	0.0733514	-1.01507
LOC100044411	0.0167906	-1.01509
POU2AF1	0.0143324	-1.01511
PBX2	0.0352608	-1.01512
FRRS1	0.0680693	-1.01512
NIPSNAP3A	0.0840544	-1.01516
LOC215098	0.0783266	-1.01518
CHAF1B	0.0152028	-1.0152
INO80C	0.0419818	-1.01521
TREX1	0.0132412	-1.01522
NAPA	0.0263426	-1.01526
PRKAG2	0.00157828	-1.01529
NUDT2	0.037891	-1.01529
MPST	0.00179764	-1.01532
CMTM4	0.015576	-1.01532
IMMP2L	0.0399928	-1.01533
EG240327	0.0470597	-1.01535
5330401P04RIK	0.0565532	-1.01548
5330431N19RIK	0.035872	-1.01551
ZFP316	0.0644596	-1.01552
1500004F05RIK	0.0674716	-1.01552
NFKBID	0.041102	-1.01554
6230425C21RIK	0.0695537	-1.01557
ARL2BP	0.0597418	-1.01558
KCTD20	0.00456676	-1.0156
B230112P13RIK	0.00767653	-1.01567
PDCD6IP	0.0640967	-1.0157
MICAL1	0.00487962	-1.01572
EAR11	0.049612	-1.01572
SCNN1A	0.00809991	-1.01573
RAB3IP	0.0481219	-1.01573
FBN1	0.0522698	-1.01574
2310007F21RIK	0.0140677	-1.01578
4930402H24RIK	0.0453866	-1.01579
LOC236604	0.0634404	-1.01579
ITM2A	0.0337057	-1.01583
LARP6	0.018749	-1.01584

1700029P11RIK	0.00165341	-1.01592
COG4	0.0385386	-1.016
SOX13	0.0693809	-1.016
AP1G2	0.0335031	-1.01602
SAMD10	0.010176	-1.01605
1600029O10RIK	0.0472837	-1.01605
TIAM2	0.0814219	-1.01607
ARRDC1	0.0557164	-1.01608
TMEM54	0.0477083	-1.0161
CDKN2B	0.0622805	-1.0161
GTF3C1	0.0115659	-1.01611
2010003O02RIK	0.0144825	-1.01614
1500015G18RIK	0.0496479	-1.01621
STMN1	0.0800169	-1.01625
NHLRC1	0.00759876	-1.01629
ACO1	0.0764968	-1.01629
ASPSCR1	0.0573053	-1.01635
MAPKAPK3	0.0574112	-1.01638
KCNIP3	0.0823206	-1.01638
DLM1-PENDING	0.0111579	-1.01645
IPAS	0.0837199	-1.0165
TMEM53	0.0673593	-1.01658
TMEM184A	0.0714011	-1.01658
RGS1	0.0184683	-1.01662
MRAS	0.0572927	-1.01662
IFT140	0.00272856	-1.01664
FSCN1	0.0212439	-1.01668
SLC46A1	0.0237985	-1.01669
CML1	0.0612225	-1.0167
NCAPD3	0.0680524	-1.01674
FSTL	0.00230971	-1.01675
GANC	0.069123	-1.01677
HC	0.00311107	-1.01684
PCMTD2	0.0596849	-1.01684
IFNB1	0.0619214	-1.01686
GPS2	0.0653646	-1.01689
2810037O22RIK	0.0654055	-1.01696
ACTR1B	0.0465175	-1.01697
ETV5	0.0611206	-1.01699
LOC225927	0.0768941	-1.01699
SCL000416.1_19	0.0119166	-1.01701
ACP2	0.0535433	-1.01702
GATAD1	0.0824893	-1.01705
2610029K11RIK	0.0132938	-1.01707
C030003A18RIK	0.00925017	-1.01708
KIFC2	0.00148004	-1.01708
ABCG4	0.010585	-1.0171
MCPT4	0.0388655	-1.01714
CD164L2	0.0797798	-1.01714
LOC100045680	0.0276332	-1.01715
SPNB3	0.030813	-1.01716
SQRDL	0.074825	-1.01728
B230339H12RIK	0.00367888	-1.01729
BEX2	0.0252394	-1.01729
CALCOCO1	0.0276955	-1.01732
POPDC2	0.00824498	-1.01736
GSTT2	0.00785553	-1.01742
HEXA	0.0199611	-1.01743

ENSMUSG00000043795	0.0493397	-1.01743
FBXL17	0.0763788	-1.01744
TTC5	0.00835426	-1.01752
MRPS28	0.0114211	-1.01752
2010002N04RIK	0.0749428	-1.01755
1700021C14RIK	0.00592209	-1.0176
LOC100046844	0.0100126	-1.01767
1110029I05RIK	0.0392584	-1.01767
PPM1K	0.0761031	-1.01771
CRIPT	0.0666177	-1.01772
2310004N24RIK	0.0654011	-1.01774
WRN	0.0186451	-1.01781
GLI3	0.0395454	-1.01783
RABL5	0.0124505	-1.01789
PAK4	0.0780871	-1.01795
RTKN	0.00403409	-1.01798
LOC209281	0.0461857	-1.01799
ADAM15	0.0620903	-1.01799
MSC	0.0261805	-1.01804
DNASE2A	0.0324597	-1.01804
9030624G23RIK	0.0163827	-1.01811
A330042I21RIK	0.00523355	-1.01815
5330417C22RIK	0.0666138	-1.01816
IL3RA	0.0334283	-1.01817
FZD10	0.0513188	-1.01818
CD248	0.0676994	-1.01819
C920006O11RIK	0.0162747	-1.01823
DNAJC12	0.00461553	-1.01824
SCD2	0.0200752	-1.01829
SART1	0.0380907	-1.01831
EG245190	0.0481048	-1.01833
PCMT1	0.0535741	-1.01835
CEP63	0.0353482	-1.01839
BCL11B	0.0730894	-1.01845
PCP2	0.0325564	-1.01848
ARAF	0.0127384	-1.01853
2310022M17RIK	0.0493626	-1.01864
ARHGAP27	0.0215687	-1.01867
2310003C23RIK	0.0278978	-1.01871
NOTCH3	0.080403	-1.01871
UBE2E3	0.0763952	-1.01872
4833421E05RIK	0.0502848	-1.01876
EPB4.1L4A	0.0129297	-1.01878
VARS2	0.0254008	-1.01878
GFOD2	0.0329331	-1.0188
D030070L09RIK	0.070656	-1.01881
CITED1	0.0462598	-1.01886
CTTN	0.00208712	-1.01889
8430410A17RIK	0.0225786	-1.01891
ADCY6	0.0392831	-1.01893
TOMM34	0.0709075	-1.019
1110008L16RIK	0.0415121	-1.01903
IGFBP6	5.23E-05	-1.01911
ALDH16A1	0.0790339	-1.01911
RPS4Y2	0.0044031	-1.0192
MXD4	0.0661213	-1.01923
RAB1B	0.00653641	-1.01926
AAAS	0.025128	-1.01928

FLAD1	0.0269929	-1.01934
D230046H12RIK	0.0506802	-1.0194
ZFP467	0.00261295	-1.01943
2610301G19RIK	0.031054	-1.01948
2810001G20RIK	0.0333915	-1.01952
ST3GAL3	0.0187859	-1.01955
RTN4	0.0677145	-1.01956
PPA2	0.0648272	-1.0196
WDR22	0.0179103	-1.01961
1700113I22RIK	0.062917	-1.01973
FAM100B	0.00647803	-1.01976
INTS7	0.0280151	-1.01984
FADS2	0.0290938	-1.01984
KATNA1	0.0225958	-1.01985
DNAHC11	0.0161966	-1.01986
CCDC132	0.0363193	-1.01986
TPM3	0.0376479	-1.01986
D430028G21RIK	0.0232438	-1.01996
PLA1A	0.0370953	-1.01999
ZMYM3	0.0804524	-1.02
NUDT22	0.0823062	-1.02
OLFM1	0.0023292	-1.02001
NR1H2	0.049974	-1.02007
DHX35	0.0216284	-1.02009
AMN	0.0601942	-1.0201
ABCC5	0.0295591	-1.02012
APLP1	0.0265708	-1.02016
LOC383308	0.0083704	-1.02023
8430432M10RIK	0.00869848	-1.02024
CYHR1	0.0373519	-1.02027
FUK	0.0172164	-1.0203
PHC1	0.0345955	-1.0203
HECTD3	0.0188571	-1.02033
A2LD1	0.0541718	-1.02037
AK2	0.0666729	-1.02038
VAV2	0.0214161	-1.02045
PRSS12	0.0368346	-1.02049
SEPHS1	0.0440424	-1.02052
TGFB1	0.0054419	-1.02058
PLTP	0.016675	-1.02062
FZD2	0.0416737	-1.02064
2610207P08RIK	0.0746243	-1.02064
PPP1R9A	0.0715339	-1.02068
IPO8	0.0319888	-1.02069
CNIH	0.00296224	-1.02071
CKLF	0.0173995	-1.02072
CENTG3	0.0426932	-1.02086
SCG3	0.0126211	-1.02088
DUSP19	0.0355715	-1.02089
TIMM44	0.0590023	-1.02091
EML3	0.0327602	-1.021
5830409B07RIK	0.00532597	-1.02103
2810405K02RIK	0.0122727	-1.02106
COL1A2	0.0549995	-1.0211
RHEBL1	0.026088	-1.02115
GM1673	0.00200172	-1.0212
PPIH	0.0225958	-1.02127
HMCN1	0.00839389	-1.02131

TSPAN6	0.00566074	-1.02142
MX1	0.00955435	-1.02149
1500009L16RIK	0.0564091	-1.02151
GNPTAB	0.0300711	-1.02161
CCDC28B	0.046496	-1.02164
FNBP1	0.0601251	-1.02165
GM106	0.0126363	-1.02166
ACPL2	0.0240197	-1.02168
BAHCC1	0.0616923	-1.02168
A530050D06RIK	0.0229904	-1.02176
PYCRL	0.0400274	-1.0218
NUDT9	0.0021588	-1.02182
6030429G01RIK	0.0612751	-1.02188
2310001H17RIK	0.0181231	-1.02196
BBS9	0.0296938	-1.02196
LOC385632	0.0295377	-1.022
HDDC2	0.0263664	-1.02203
2310067B10RIK	0.0597575	-1.02209
GAS8	0.0169205	-1.02213
AI413582	0.0600941	-1.02215
CHPT1	0.0314133	-1.02222
SNX12	0.00774983	-1.02232
ASCC1	0.0227499	-1.02232
SLC27A1	0.0820692	-1.02234
COG1	0.0271128	-1.02235
AHCY	0.0170986	-1.02241
PRR12	0.0810204	-1.02241
IKBKG	0.0137861	-1.02243
TBCD	0.0293814	-1.02244
PRKCA	0.0223863	-1.0226
CYP2J9	0.0282253	-1.02265
DYRK1B	0.0535663	-1.02265
CDK9	0.0342479	-1.02275
XPC	0.0212565	-1.02279
HEPH	0.03273	-1.0228
FAM116B	0.0327943	-1.02282
B130019G13RIK	0.0646622	-1.02288
DDB2	0.0398269	-1.02289
LOC381501	0.0490117	-1.02292
5330438D12RIK	0.0773141	-1.02308
IL17RC	0.0566609	-1.0231
A630072M18RIK	0.030123	-1.02315
NIT2	0.0368617	-1.02318
NRADD	0.0399411	-1.0232
NUMA1	0.043381	-1.02322
LOC382691	0.000507643	-1.02324
6330549D23RIK	0.00359638	-1.02325
2210011G09RIK	0.0199981	-1.02329
CCL19	0.00484491	-1.02331
LSM2	0.0728882	-1.02341
DDHD2	0.0119765	-1.02342
IGSF10	0.0232085	-1.02342
UBE2L6	0.00497013	-1.02349
B230373P09RIK	0.0261427	-1.02351
4.93E+26	0.0480832	-1.02354
LOC381996	0.0350252	-1.02363
FGFR1	0.069173	-1.02364
SURF1	0.0179154	-1.02377

LOC382096	0.0752945	-1.02378
PPFIBP2	0.0538631	-1.02383
BICC1	0.0240798	-1.02388
TMEM40	0.0271698	-1.02394
EG633570	0.0769337	-1.02396
ATP6V1G1	0.0296359	-1.024
CREB3L4	0.0717111	-1.024
CBX7	0.0422653	-1.02401
ARHGEF1	0.0379516	-1.02403
CTNNBL1	0.0617633	-1.02403
LOC382707	0.0508746	-1.02405
OASL1	0.00406919	-1.02418
SLC25A45	0.00909804	-1.02419
CACNB3	0.00682008	-1.02436
9530027K23RIK	0.0505058	-1.02437
A730017D01RIK	0.0233638	-1.02439
EIF2B1	0.0190522	-1.02449
ABCA7	0.0526738	-1.02451
ZFP704	0.0634885	-1.02452
2610204M08RIK	0.00402606	-1.02454
LYZ2	0.060435	-1.02454
ADAR	0.0323368	-1.02455
9030624J02RIK	0.0263322	-1.02464
2610208M17RIK	0.0344145	-1.02465
ATG10	0.0677734	-1.02473
C1QC	0.0415296	-1.02474
C130027E04RIK	0.0672027	-1.02474
RNF113A2	0.0227389	-1.02477
ELK3	0.0662114	-1.02481
DDX5	0.0120787	-1.02482
PML	0.0468859	-1.02484
EMID1	0.0443149	-1.02485
1300011L04RIK	0.00183632	-1.02492
2310009B15RIK	0.0740415	-1.02494
IL33	0.0496044	-1.02496
MAP3K6	0.0375463	-1.025
TWF2	0.0470734	-1.02507
LMTK2	0.0736966	-1.02508
DALRD3	0.027972	-1.02513
SLC9A3R2	0.0757237	-1.02515
4930533K18RIK	0.0764402	-1.02516
ZFP710	0.00605418	-1.02519
CD1D1	0.00432292	-1.02521
SCRIB	0.0196628	-1.02525
FMNL3	0.0666773	-1.02525
MMP23	0.0127915	-1.02529
PPIL3	0.0494528	-1.0254
TBL1XR1	0.0165506	-1.02545
1810013B01RIK	0.0208122	-1.02557
TRIM34	0.0465309	-1.02558
LRRC51	0.0614631	-1.02562
TSHZ3	0.0748075	-1.02563
PLCD1	0.0534646	-1.0257
NELF	0.0815464	-1.02574
CTSF	0.00435732	-1.02576
TBC1D25	0.0318869	-1.02599
UBL4	0.0229614	-1.026
RETSAT	0.0294405	-1.02603

LOC100047260	0.0397752	-1.02604
ATP5E	0.0599656	-1.02604
MBTPS1	0.0329938	-1.02608
WIPF3	0.0353166	-1.02611
ADAMTS7	0.0508918	-1.02624
1200011O22RIK	0.0824099	-1.02628
2010110K16RIK	0.000772221	-1.02635
RAB7L1	0.00319061	-1.02637
HRSP12	0.0285349	-1.02642
D16H22S680E	0.0260082	-1.02648
SNURF	0.025807	-1.0265
TNFRSF18	0.0207804	-1.02653
SCL0003651.1_17	0.0202536	-1.02656
CEP164	0.00430198	-1.02663
TMEM218	0.0674019	-1.02663
ACSBG1	0.0404607	-1.02675
LOC380706	0.0542997	-1.02682
DGUOK	0.0575985	-1.02685
BC064033	0.05695	-1.02686
BTBD6	0.0803856	-1.02687
DACT2	0.07133	-1.02689
GPR137	0.078218	-1.02695
CCHCR1	0.0452862	-1.02706
DCXR	0.019519	-1.02722
TRP53INP2	0.0558645	-1.02725
D130064L03RIK	0.00494691	-1.02734
HIST2H2AA1	0.0400679	-1.02738
2610003J06RIK	0.078641	-1.02739
CTBP2	0.0440784	-1.02746
LOC639086	0.023032	-1.02758
HDAC6	0.0249422	-1.0276
HDC	0.0302342	-1.02761
NDUFB10	0.0594969	-1.02762
CENPT	0.0270181	-1.02763
FBXL20	0.0350956	-1.02768
ATP6V1C2	0.0652458	-1.02775
CCDC12	0.0330275	-1.02805
PRKAG1	0.0033944	-1.0281
PTGIS	0.0082062	-1.02822
CCDC5	0.0184293	-1.0283
SLC2A4	0.0230944	-1.02831
VPS26B	0.0656348	-1.02836
CADPS2	0.00704653	-1.02846
FCGR4	0.0435917	-1.02866
GMCL1	0.051655	-1.02872
EIF4B	0.06724	-1.02873
RFFL	0.00736591	-1.02881
CASZ1	0.0131219	-1.02885
A930026I22RIK	0.0324214	-1.02886
SEPT9	0.0377271	-1.0289
D930048N14RIK	0.0114191	-1.02893
SMPDL3A	0.0655732	-1.02893
WDR60	0.0189628	-1.02896
ATF3	0.0232068	-1.02899
TNFSF12-TNFSF13	0.00602897	-1.02904
FLII	0.00330068	-1.02905
MPI	0.081723	-1.02905
TRIM26	0.0321285	-1.02909

3110013H01RIK	0.0571769	-1.02917
LOC329575	0.0829585	-1.02921
MAX	0.0673307	-1.02933
BRD9	0.0756251	-1.02935
MYG1	0.00576531	-1.02939
SEC14L1	0.0141715	-1.02946
CAV2	0.028176	-1.02946
RABEP2	0.0821091	-1.02953
EFCAB4A	0.0355027	-1.02958
LYNX1	0.00714412	-1.02961
CCRK	0.0622536	-1.02966
MYCBPAP	0.00173758	-1.02971
2310079N02RIK	0.0218586	-1.02977
PADI1	0.0831618	-1.0299
AA536749	0.0330949	-1.02993
4121402D02RIK	0.00740039	-1.02997
LGSN	0.00942537	-1.02998
PFKM	0.0236621	-1.03012
NR2F6	0.0664703	-1.03015
LSR	0.0027173	-1.03016
ANG	0.0449049	-1.03016
TXLNA	0.0746073	-1.03023
NIT1	0.0325051	-1.03032
P2RX4	0.00561353	-1.03043
MCCC1	0.0700127	-1.03043
2610524H06RIK	0.00047628	-1.03045
0610037D15RIK	0.00541473	-1.03048
DEXI	0.0748524	-1.0305
SIN3B	0.0619192	-1.0307
BC051227	0.0392786	-1.03071
PALM	0.0123886	-1.03074
ZFHX3	0.0512131	-1.03075
GM684	0.00323392	-1.03078
2010111I01RIK	0.0168876	-1.03084
2510008P16RIK	0.0616829	-1.03089
1600016N20RIK	0.076047	-1.03091
CCNDBP1	0.0139241	-1.031
SMOC1	0.00872926	-1.03102
LOC230896	0.0117198	-1.03109
1190005F20RIK	0.015015	-1.03116
TSPYL3	0.000966025	-1.0312
RMND1	0.061021	-1.03122
NME7	0.0624147	-1.03142
ATP1B1	0.0397456	-1.03144
ESRRA	0.0249476	-1.03149
RFTN2	0.0387162	-1.0315
INPP5F	0.0815395	-1.0315
ZKSCAN17	0.0365209	-1.03154
D630036H09RIK	0.0399754	-1.03154
PI16	0.0715417	-1.03157
LOC383706	0.0525698	-1.03159
COL1A1	0.00793719	-1.03167
CH25H	0.0822669	-1.03177
GOLGA7	0.0179303	-1.0318
HPCAL1	0.032204	-1.03185
ACADS	0.0688272	-1.03186
MXRA8	0.0102819	-1.03188
RNASEH2A	0.0233461	-1.03188

C030003D03RIK	0.0543368	-1.03194
LOC674706	0.0728427	-1.03226
ZCCHC8	0.0223965	-1.03227
NTNG1	0.0781099	-1.03228
MRPL27	0.00123343	-1.03235
RNF135	0.0558358	-1.03248
MRPL4	0.0169447	-1.03265
FHOD1	0.0189383	-1.03265
5430437P03RIK	0.019087	-1.0327
HOMER2	0.0573289	-1.03273
GPX7	0.00256887	-1.03279
WBP1	0.00493846	-1.03288
PTMS	0.0684174	-1.03293
MAPRE2	0.0371923	-1.03298
TST	0.00884068	-1.03313
1190005N23RIK	0.0132485	-1.03319
SSH3	0.0485161	-1.0333
CTXN1	0.00131473	-1.03332
RBM47	0.0348065	-1.03335
ZFHX2	0.0133915	-1.03337
CRYL1	0.0514845	-1.03338
UBE2Q1	0.0717682	-1.03341
ZCCHC18	0.0504445	-1.03344
GUSB	0.050433	-1.03348
MEN1	0.0245823	-1.03358
ORAI1	0.0829096	-1.03377
TSC22D4	0.0643575	-1.03388
2310021P13RIK	0.00178817	-1.03396
ARHGEF16	0.0428511	-1.03398
WDR6	0.019884	-1.03399
TPD52L1	0.0684481	-1.03406
CPXM1	0.0458631	-1.03407
UBXN6	0.0217666	-1.03412
IAH1	0.00202541	-1.03417
LOC100045019	0.0790236	-1.0343
EG245297	0.0218455	-1.03434
ASB13	0.0425337	-1.03439
LAD1	0.00789714	-1.03444
SLC29A3	0.0177259	-1.03461
LOC217602	0.0764588	-1.03462
MANBA	0.0501192	-1.03485
OLFML3	0.0111416	-1.03489
0610009B22RIK	0.0310938	-1.03493
BLMH	0.0456187	-1.03494
PPT1	0.0016431	-1.03519
OPLAH	0.0200742	-1.03521
PCBD2	0.0111907	-1.03534
MARCH2	0.0781715	-1.03554
H2-T10	0.000912068	-1.03555
ATP13A2	0.0513016	-1.03564
C1S	0.000127628	-1.03569
RNUXA	0.0156955	-1.03577
AP2A2	0.0364197	-1.0358
CCDC80	0.016842	-1.03584
AUP1	0.0431825	-1.03585
SNN	0.00151066	-1.03592
RAB4A	0.0130303	-1.03594
MLP	0.0152525	-1.03602

RNF4	0.0849241	-1.03603
DOCK6	0.0127812	-1.03606
STRN4	0.0357056	-1.03608
PNPLA1	0.00365119	-1.0362
ENTPD2	0.0190361	-1.03626
IFI35	0.038908	-1.03627
MCM4	0.08131	-1.03633
B4GALT1	0.0190671	-1.03634
GM561	0.061687	-1.03634
SLC39A4	0.0828091	-1.03636
NUB1	0.0309688	-1.03637
BC018462	0.081919	-1.03637
ZFP787	0.0182081	-1.0364
MLKL	0.0268911	-1.0364
SHD	0.0141424	-1.03642
NFATC4	0.02461	-1.03656
BCKDK	0.0195662	-1.0366
1200016B10RIK	0.00477529	-1.03665
LOC100043919	0.0335792	-1.03666
B3GNT1	0.0687679	-1.03668
LOC383125	0.0587046	-1.03669
D12ERTD553E	0.0117687	-1.0367
ZFP672	0.0118153	-1.0367
CLCA3	0.00376904	-1.03673
LETMD1	0.00533903	-1.03677
PIN4	0.00398112	-1.03677
MAPK8IP1	0.0219324	-1.03683
ERCC2	0.00133606	-1.03685
2610307O08RIK	0.0201238	-1.03692
LOC669658	0.012754	-1.03693
INSL6	0.01011	-1.03695
RCE1	0.0127496	-1.03704
MED25	0.0511166	-1.03706
CHURC1	0.0135835	-1.03724
WNK2	0.00162923	-1.03727
1110012L19RIK	0.0201462	-1.03734
ADD1	0.0298727	-1.03734
0610011F06RIK	0.00207604	-1.03736
FAM171A2	0.0138621	-1.0375
CRAMP1L	0.0610683	-1.03754
RAP1GAP	0.0333192	-1.03759
LMAN2L	0.015774	-1.03763
USP3	0.0138451	-1.03765
LGALS4	0.0359426	-1.03768
SMARCC2	0.0628575	-1.03769
CTSA	0.0694735	-1.03779
TMEM41A	0.0195747	-1.0378
DPT	0.0178893	-1.0379
PEX5	0.0809533	-1.0379
LOC383775	0.0148689	-1.03799
LANCL1	0.00283905	-1.03801
DYNC2LI1	0.0507559	-1.03818
CBX6	0.0799539	-1.03818
1810014F10RIK	0.0610605	-1.03828
4930455C21RIK	0.0129266	-1.03832
IFI30	0.0620912	-1.03834
GPT1	0.00823698	-1.03841
ANKRD50	0.0624217	-1.03841

EFNA4	0.000294684	-1.03848
PLSCR1	0.0498745	-1.0385
PRKAR2B	0.0844562	-1.03857
PRDX3	0.060224	-1.03858
STX1A	0.0346222	-1.03866
TMEM19	0.00798294	-1.03872
ACADL	0.0218752	-1.0389
MRPL9	0.0127516	-1.03898
LOC100046039	0.04268	-1.03898
WIF1	0.0615928	-1.03899
ST13	0.0489967	-1.03906
PROM2	0.0149958	-1.03918
LOC100047486	0.0796562	-1.03932
FAM13B	0.0582688	-1.03933
PYGB	0.0380427	-1.03935
A730063M14RIK	0.0820032	-1.03935
SCNN1B	0.0226792	-1.03951
CAPSL	0.0284158	-1.03956
INTS10	0.0744602	-1.03966
PDE6D	0.0147952	-1.03972
F8A	0.0175435	-1.03974
TMEM179B	0.0673561	-1.03978
2010007H12RIK	0.0163744	-1.03989
STAP2	0.0179647	-1.03993
1110059G02RIK	0.0389489	-1.03995
STAT1	0.0316277	-1.03997
AI987712	0.021944	-1.04006
TUBB2B	0.0440916	-1.04006
LSM3	0.0245307	-1.04011
PLA2G7	0.0170152	-1.04034
UBE2V1	0.00280897	-1.04037
LOC100044430	0.0122912	-1.04037
MRPL11	0.0128226	-1.04046
PALMD	0.0803531	-1.04046
LOC627317	0.0225909	-1.04055
RABL2A	0.00648849	-1.04072
PLD1	0.0506713	-1.04073
DDB1	0.00847257	-1.04078
RBM43	0.000644414	-1.04078
1110032A13RIK	0.0741695	-1.04081
LOC381215	0.0024697	-1.04085
4933436C20RIK	0.0245402	-1.04086
CANT1	0.0156746	-1.04087
DLL1	0.0204336	-1.04089
ITGA8	0.0752634	-1.04089
SCL0001419.1_32	0.0741845	-1.04108
SLC44A1	0.0375569	-1.04109
LOC667269	0.0108941	-1.04112
GTPBP3	0.016267	-1.04137
BCL2L11	0.0492013	-1.04138
HNRPLL	0.0613109	-1.04138
ALDH3A2	0.0773993	-1.04149
4931406C07RIK	0.0117593	-1.04152
NT5C3L	0.0255876	-1.04154
BRF1	0.0493322	-1.04183
LOC381889	0.00385262	-1.04185
RGS16	0.0445082	-1.04185
IGBP1	0.0483142	-1.04193

D030051N19RIK	0.0465771	-1.04195
GSTZ1	0.0346228	-1.04196
ZFP523	0.0395109	-1.04198
TNFRSF11B	0.0761753	-1.04208
SMPDL3B	0.0191986	-1.04217
CLIP2	0.0393517	-1.0422
KHDRBS1	0.000575607	-1.04236
TTC39A	0.0697792	-1.04238
TM7SF3	0.0516379	-1.0425
KIF5C	0.0109829	-1.04262
CDKN1C	0.0389144	-1.04266
RELL1	0.0731685	-1.04267
SLC25A20	0.0472647	-1.04278
FBLN2	0.0378878	-1.04287
PYGO2	0.0318337	-1.04289
NAV1	0.0479408	-1.04298
MAEA	0.0197133	-1.04299
GTF3A	0.0120076	-1.04301
1110012D08RIK	0.0247234	-1.04301
F630025I20RIK	0.0403938	-1.04309
LOXL1	0.00103047	-1.04315
LOC100043126	0.0553967	-1.04319
TGM1	0.0493207	-1.04339
COL8A1	0.0705681	-1.04354
VPS37D	0.0628093	-1.04361
ECM1	0.0605029	-1.04367
2010011I20RIK	0.0660547	-1.0437
MGAT4B	0.0837839	-1.04374
PGLS	0.0169574	-1.04384
2310040A07RIK	0.0302199	-1.0439
MEGF8	0.0438644	-1.04406
CCND3	0.0575981	-1.04413
LOC100040353	0.0707177	-1.04419
SMARCA1	0.0743466	-1.04419
HAGHL	0.013017	-1.0443
SYNGR2	0.061285	-1.0443
EG384179	0.0629909	-1.04431
LARP1	0.0641995	-1.04451
RBM38	0.022868	-1.04497
LOC667776	0.0284065	-1.04498
LOC100043822	0.0261008	-1.04527
9430016H08RIK	0.00940127	-1.0455
ARF5	0.0452054	-1.0455
EPS15	0.0566656	-1.04551
BCL2A1B	0.0764635	-1.04551
RASGRP4	0.0486662	-1.04553
SSBP4	0.0506778	-1.04557
SIRT3	0.0114705	-1.04558
UGT1A6B	0.0365477	-1.04561
ECHS1	0.0425714	-1.04576
BC006779	0.0143017	-1.04584
H2-DMB1	0.0161293	-1.04586
KCNMB1	0.0272232	-1.04609
LOC545208	0.0281361	-1.04628
AI451557	0.00335483	-1.04636
C1QB	0.0557399	-1.04636
CDK2	0.00813795	-1.04637
MRPL48	0.0191885	-1.04644

LCMT1	0.0153068	-1.04656
MAPK14	0.014809	-1.04657
ILVBL	0.00551202	-1.04669
EIF4E3	0.0623201	-1.04695
TMEM79	0.00777767	-1.04706
LOC333331	0.0301329	-1.04712
HES1	0.0293397	-1.04715
NID1	0.0312	-1.04723
FAM110C	0.0220113	-1.04732
MACROD1	0.0450449	-1.04732
GIPC2	0.0486914	-1.04769
PQLC3	0.0490101	-1.04777
2510010F15RIK	0.0367448	-1.04784
OSMR	0.0116328	-1.04786
RABL4	0.0122241	-1.04805
NDUFB5	0.00705662	-1.04835
FAM125A	0.0532755	-1.04842
LOC225897	0.0619763	-1.04843
B930041F14RIK	0.0489505	-1.04845
LOC100048376	0.0129577	-1.04849
LONP2	0.0435231	-1.04861
4930504E06RIK	0.0564841	-1.04863
ZDHHC12	0.0443049	-1.04866
TEF	0.0808091	-1.04868
GSTT1	0.0384821	-1.04872
LOC668837	0.0687501	-1.04876
AP4S1	0.0218484	-1.04882
D930015E06RIK	0.00638198	-1.04884
LAGE3	0.0115757	-1.04884
DLK2	0.0462941	-1.04888
NDN	0.00126984	-1.04919
RSU1	0.0748825	-1.04936
ROBLD3	0.00710707	-1.0495
CRYAB	0.0725008	-1.04954
H2-EB1	0.0261962	-1.04955
PLCB3	0.00431778	-1.04969
1810020D17RIK	0.0166925	-1.0498
TRAPPC3	0.0126148	-1.04983
COMMD10	0.0160886	-1.04988
NDUFS2	0.0294122	-1.05001
RAB4B	0.0809031	-1.0501
2310045N01RIK	0.0128856	-1.05012
CHD8	0.0783674	-1.05014
TANK	0.000165494	-1.05033
PLCG2	0.0284466	-1.05033
2900057K09RIK	0.033169	-1.0504
D930017K21RIK	0.0141869	-1.05047
2300009A05RIK	0.0146943	-1.05048
YIPF1	0.0524456	-1.05049
CTSZ	0.0650015	-1.05049
ENPP5	0.0141142	-1.05063
SHISA2	0.00764579	-1.05087
TCF3	0.001111	-1.05096
PEF1	0.0743775	-1.05097
LOC621823	0.000753484	-1.05099
2410008K03RIK	0.0803929	-1.051
D130059O18RIK	0.0298416	-1.05102
AI316807	0.0463136	-1.0511

FANCE	0.00766749	-1.05111
CTNNBIP1	0.00842236	-1.05116
TTC13	0.0764819	-1.05132
LOC383826	0.0300531	-1.05138
KAT2A	0.0168812	-1.05143
PGCP	0.034285	-1.05146
TAPBP	0.022803	-1.05156
ZFP313	0.0708339	-1.05157
ZNHIT1	0.00759211	-1.05161
SCL0001647.1_23	0.00200582	-1.05163
COL6A3	0.00757365	-1.05176
HTATIP2	0.0420955	-1.05179
BAD	0.0125914	-1.0518
DDX58	0.00792892	-1.05193
RNF167	0.00348813	-1.05199
LOC100042179	0.000596803	-1.05205
SEMA4A	0.0289301	-1.05211
IFIT2	0.0408376	-1.05221
B3GNTL1	0.0454579	-1.05245
5031439G07RIK	0.0395233	-1.05251
WASF1	0.0277503	-1.05266
C2CD3	0.00391702	-1.05268
TMEM55A	0.0463219	-1.0527
RHOG	0.0550397	-1.05273
TMEM134	0.0057973	-1.05298
TMEM98	0.0418772	-1.05304
PLEK2	0.0743312	-1.0534
COL4A6	0.0697455	-1.05354
LOC100044702	0.00638826	-1.05359
LPL	0.00175125	-1.05365
LOC100044221	0.0207282	-1.05369
PPAP2C	0.00645621	-1.05403
H2-Q6	0.0373387	-1.05414
IGF2R	0.0817895	-1.05422
D930046M13RIK	0.0734949	-1.05425
ZFP664	0.0209904	-1.05427
TPSAB1	0.046161	-1.05433
SAMHD1	0.044674	-1.05445
AKR1B8	0.0298513	-1.05451
LOC100045612	0.051305	-1.05463
NKD2	0.0195417	-1.05481
MTVR2	0.0510435	-1.05507
GSTP2	0.0668905	-1.0551
PSMD10	0.02511	-1.05518
POLD4	0.0363471	-1.05525
RDM1	0.0454188	-1.05542
VIM	0.0691575	-1.05543
2810423O19RIK	0.0744923	-1.05544
LOC100048301	0.000359065	-1.05551
ARPC1A	0.0695778	-1.05569
NDUFB9	0.0122151	-1.05581
SSBP3	0.0815292	-1.05582
CYP4F13	0.00805794	-1.05599
LPCAT3	0.0216184	-1.05608
MRPS16	0.0292974	-1.05608
MTCH2	0.0420926	-1.05659
THEM5	0.0294923	-1.0567
2410025L10RIK	0.0124849	-1.05694

1110058L19RIK	0.0199695	-1.05698
TMEM29	0.00354523	-1.05723
PLEC1	0.0224994	-1.05728
TOMM40L	0.00606784	-1.05733
GDI1	0.0454373	-1.05749
PLD3	0.0364891	-1.05776
TAP1	0.00772912	-1.05778
CTSK	0.0135162	-1.05824
SOSTDC1	0.0847459	-1.05826
ADCK5	0.00809635	-1.05827
DHX58	0.002365	-1.05828
IRF2BP1	0.0753683	-1.05842
5930416I19RIK	0.0566674	-1.05845
PJA1	0.0205653	-1.05847
BCAS1	0.0123201	-1.05849
PAOX	0.00920195	-1.05855
LOC100045280	0.00455432	-1.05867
ZFP579	0.00316146	-1.05869
ERBB2	0.00867587	-1.05899
STAT5A	0.00996975	-1.05911
CMTM6	0.0766755	-1.05924
AU022508	0.0608846	-1.05931
MAPBPIP-PENDING	0.0294346	-1.05936
KCTD10	0.0583994	-1.05936
ALG14	0.0336551	-1.05939
HBP1	0.0286148	-1.05945
TSPAN17	0.0449069	-1.05956
LOC545487	0.0183907	-1.05962
ABHD11	0.0419518	-1.06015
PRKACA	0.0139169	-1.06027
BACE1	0.00242574	-1.0603
GDPD1	0.0263152	-1.0604
COPS5	0.0451265	-1.06045
SLC24A6	0.0350183	-1.06056
4933428A15RIK	0.0777461	-1.06066
ELOF1	0.071232	-1.06076
TAZ	0.0197499	-1.06077
CIRBP	0.000105524	-1.06084
MMP15	0.063826	-1.06086
D14ERTD449E	0.0314008	-1.06106
SPNA2	0.0121456	-1.06111
SENP3	0.070992	-1.06116
DEF6	0.00513652	-1.06122
EG433865	0.0127388	-1.06149
3110009E18RIK	0.0375234	-1.06195
2410003K15RIK	0.0442033	-1.06196
SIDT2	0.0158187	-1.06201
TCN2	0.0541166	-1.06229
BIK	0.023377	-1.06283
IGFALS	0.0624434	-1.06283
PACS1	0.078018	-1.06289
SMARCA2	0.0423969	-1.06293
D9ERTD392E	0.0293024	-1.06294
EPS8	0.0231065	-1.06313
ZFP282	0.044592	-1.0633
STX4A	0.00197006	-1.06334
HMG20B	0.0428421	-1.06348
TMEM106C	0.0773326	-1.06369

RNASET2	0.0088851	-1.06387
KLC4	0.010489	-1.06425
HSPE1	0.00715351	-1.06444
CPT2	0.03199	-1.06444
MANBAL	0.0565236	-1.06454
1500001M20RIK	0.00251964	-1.06457
SEPP1	0.0548988	-1.06461
MRPL34	0.0060495	-1.06475
MFAP2	0.0234605	-1.0649
RIT1	0.0742751	-1.06494
PKP3	0.0195939	-1.06498
RNF114	0.0565906	-1.06511
TRIP6	0.0104478	-1.06515
YKT6	0.0773385	-1.06532
ACBD4	0.0450313	-1.06536
GALNTL4	0.0178211	-1.06541
TMPRSS13	0.0272545	-1.06541
SNHG11	0.034745	-1.06547
THTPA	0.029296	-1.0655
2300002D11RIK	0.0462847	-1.06565
POLRMT	0.0750481	-1.06597
CAB39L	0.00768705	-1.06604
ELL	0.0547469	-1.06634
ATP5F1	0.0656277	-1.0664
QARS	0.0699193	-1.06692
H6PD	0.00493822	-1.06702
ARD1	0.0169456	-1.06706
MYO18A	0.0475863	-1.06736
CNN2	0.0131633	-1.06741
SEPN1	0.0417144	-1.06749
IRF3	0.0655778	-1.06752
PSME2	0.00890873	-1.06765
TMEM63A	0.0507545	-1.06777
H2-AA	0.0255274	-1.0679
SH3GLB2	0.020907	-1.06807
SCL0002507.1_236	0.0268067	-1.06831
SCT	0.0420544	-1.06859
CCDC23	0.0810667	-1.06869
ZDHHC4	0.0198244	-1.06883
2410018G20RIK	0.0108578	-1.06889
TMEM176A	0.0573683	-1.06893
CXCL10	0.00432227	-1.06902
M6PRBP1	0.0732857	-1.06922
TRAPPC1	0.00307137	-1.06926
LLGL2	0.0639525	-1.06947
EG216185	0.046652	-1.0696
TCIRG1	0.0444776	-1.06965
2200001I15RIK	0.0783613	-1.06988
X99384	0.0107314	-1.07024
LOC100046003	0.0401694	-1.07033
LOC100046616	0.00438064	-1.07051
EHMT2	0.013645	-1.07058
CCDC53	0.0280921	-1.0708
HDAC7	0.0056479	-1.07084
OAS1G	0.0162609	-1.07118
LOC638301	0.0343348	-1.0712
FAM109A	0.0388103	-1.07126
MITD1	0.0394327	-1.07128

TCTEX1D2	0.0197298	-1.07134
TCEAL8	0.0128664	-1.07145
ASS1	0.0357133	-1.07148
MRPL22	0.0279349	-1.07162
LAMC2	0.0793793	-1.07187
LOC100047012	0.0212563	-1.07203
MRPS21	0.0393808	-1.07204
GINS4	0.0395752	-1.07207
PDGFRA	0.0430158	-1.0722
ZNRD1	0.0338263	-1.07234
TRIM41	0.0261969	-1.07267
NOD1	0.0377797	-1.07276
DCTN1	0.0409515	-1.07289
NDST2	0.0679163	-1.07298
PPP1R9B	0.0477895	-1.07378
TMEM159	0.0514375	-1.07381
LSM5	0.0186282	-1.0741
GPR153	0.021607	-1.07413
NDUFS3	0.0652508	-1.07423
ACAA2	0.00551335	-1.07429
4933412E12RIK	0.0151382	-1.07452
BLOC1S1	0.0175647	-1.07469
4931426K16RIK	0.0373792	-1.07477
OXA1L	0.0474156	-1.07481
ALDH4A1	0.0246502	-1.07498
ELOVL1	0.0274522	-1.07531
1810013D10RIK	0.0797118	-1.07536
MYO9B	0.00710413	-1.07551
SCL0003781.1_22	0.0624985	-1.07582
CDK4	0.0687895	-1.07613
PKIA	0.0423574	-1.07634
CLTA	0.0332571	-1.07636
PRSS8	0.0810448	-1.07639
SLC9A3R1	0.0127529	-1.07646
RHOC	0.0280767	-1.07647
RPL30	0.0308149	-1.0765
DGKA	0.00317419	-1.07652
SUMO3	0.064907	-1.07677
B230369L08RIK	0.0804353	-1.07686
TRIB2	0.026131	-1.07699
RNF5	0.0157665	-1.07722
GNG10	0.0846376	-1.07726
LOC100048556	0.0394255	-1.0775
TNIK	0.0526439	-1.0777
AI607873	0.0183461	-1.07799
2310075M15RIK	0.00759753	-1.07807
HR	0.0458865	-1.07848
CXXC1	0.0151767	-1.07851
PIGP	0.0551299	-1.07864
ADSS	0.0201747	-1.07907
KLHL17	0.0221329	-1.07921
ARL3	0.00309371	-1.07922
CD200	0.0072231	-1.07935
HECA	0.0569194	-1.07941
4933421H10RIK	0.021764	-1.07989
SCL000292.1_12	0.0130451	-1.07993
SSNA1	0.0130108	-1.0802
POF1B	0.0276709	-1.08043

1500010J02RIK	0.0239847	-1.08049
SUCLG1	0.0162565	-1.08051
TMEM88	0.00240006	-1.0806
LOC100046744	0.0467128	-1.08087
TMC4	0.0163646	-1.08099
SORT1	0.0423751	-1.08122
ACVR2B	0.0591038	-1.08125
APOA1BP	0.0393521	-1.08128
ZFP68	0.0170758	-1.08143
DLG3	0.0666692	-1.08158
MOCOS	0.0534107	-1.08163
2310005E10RIK	0.0381636	-1.08164
DHRS1	0.0605554	-1.08174
CIB1	0.0436549	-1.08179
B230333E16RIK	0.0210666	-1.08186
OCIAD1	0.0840389	-1.08193
SUPT3H	0.0780429	-1.08216
ANKMY2	0.0127556	-1.08221
HNRNPK	0.0288973	-1.08225
LOC100047963	0.0795175	-1.08243
PIK3IP1	0.0454248	-1.08361
NACC2	0.0576753	-1.08371
TMC6	0.0158462	-1.08462
AI646023	0.0536506	-1.08473
OTTMUSG00000007855	0.0363239	-1.08477
EPDR1	0.0737755	-1.08496
CAPG	0.00626621	-1.08511
HSBP1	0.0358119	-1.08524
TAF10	0.0740144	-1.08527
RPL13A	0.00365542	-1.0853
LOC627985	0.0707973	-1.08546
LAMA5	0.0619706	-1.08558
2010107H07RIK	0.00663274	-1.08559
BAT5	0.027551	-1.08594
DGKZ	0.0658055	-1.08619
TDRD7	0.043154	-1.08638
AES	0.0391354	-1.08653
GALT	0.00951232	-1.08655
IRF7	0.0134091	-1.08687
5133401N09RIK	0.00485707	-1.08696
PLXNB2	0.0176033	-1.08708
CUTA	0.0360273	-1.08736
PRKAB1	0.0408235	-1.08736
SLC9A1	0.0360484	-1.08738
MGC18837	0.0304734	-1.08772
MED24	0.040117	-1.08774
LOC244710	0.0579216	-1.08813
1810006K21RIK	0.0389388	-1.08828
ARHGEF19	0.00294254	-1.08829
PRKRA	0.0375641	-1.08834
ITGB1BP1	0.0302872	-1.08843
COMMD4	0.0617669	-1.08859
CXCL12	0.0465719	-1.08915
LOC100047936	0.0314133	-1.08962
NRM	0.0322571	-1.08965
DAB2IP	0.0787798	-1.08983
SCP2	0.0128581	-1.08993
S100A14	0.0463312	-1.08993

MAP3K11	0.0833685	-1.08993
CTSC	0.0503492	-1.0903
STARD10	0.0378592	-1.09132
NME3	0.0114289	-1.09169
NHSL1	0.0141328	-1.09222
3110001D03RIK	0.0156157	-1.09229
ZNF512B	0.0154477	-1.09242
LUM	0.0300677	-1.09264
PARP12	0.00932992	-1.09266
MRPL30	0.0108544	-1.09281
2610027C15RIK	0.0772469	-1.09281
TLE6	0.026212	-1.09298
MUM1	0.0761772	-1.093
AGPS	0.0316137	-1.09351
PSKH1	0.045512	-1.09378
E130102H24RIK	0.00672209	-1.09383
LAPTM5	0.0846348	-1.09393
LOC100046918	0.0750436	-1.09403
IFT81	0.0764268	-1.09415
JUN	0.0642187	-1.0945
NUDT16L1	0.046831	-1.09455
TSPAN3	0.0573333	-1.09465
EI24	0.0556403	-1.09523
TSC2	0.0128503	-1.09528
C4B	0.0733341	-1.09532
D10ERTD322E	0.0327553	-1.0955
RFC2	0.0190047	-1.09556
CBR1	0.00592977	-1.09576
HMGCS2	0.00539633	-1.09592
TSEN34	0.0500029	-1.09623
MIC2L1	0.0363868	-1.09633
1300013J15RIK	0.0234557	-1.09639
VWA5A	0.00575345	-1.09653
YWHAH	0.0419067	-1.0966
KREMEN	0.0266888	-1.09692
9430029L20RIK	0.0315338	-1.09696
2700038C09RIK	0.0353453	-1.09699
HYI	0.0243154	-1.09743
2310005N03RIK	0.0388542	-1.09749
MYADM	0.0102996	-1.09786
BC014795	0.0151355	-1.0981
RTN3	0.00540401	-1.09817
RELN	0.0230936	-1.09845
HIST2H2AA2	0.0542362	-1.09856
SCAND1	0.050971	-1.09867
CARKD	0.0142295	-1.09876
ARF3	0.0096991	-1.09907
LY6D	0.0485405	-1.09908
2810410M20RIK	0.0120379	-1.09916
CORO1B	0.0720976	-1.09946
ETFB	0.0501323	-1.09971
BC002163	0.00148242	-1.10001
SORL1	0.0343138	-1.10002
GNB2	0.0757378	-1.10025
FLYWCH2	0.00268458	-1.10026
AW228700	0.000706754	-1.10035
ANO1	0.0168666	-1.10096
RPL29	0.019956	-1.10111

MAP1LC3B	0.0138979	-1.10132
H2-Q7	0.0146179	-1.10136
MAPK13	0.0617044	-1.10158
GLO1	0.081716	-1.10222
FOXA3	0.0659524	-1.10264
GNAI2	0.0669723	-1.10286
FBXW17	0.0179358	-1.10405
EG629383	0.0209187	-1.10405
LOC226017	0.00511418	-1.10433
AP3D1	0.0231829	-1.10468
FKBP8	0.0484059	-1.10584
DPP7	0.00484009	-1.10596
INMT	0.0547995	-1.10625
LY6C1	0.0377136	-1.10679
AQP9	0.0225693	-1.1068
CTNNAL1	0.00877335	-1.10739
ST6GAL1	0.0718835	-1.10754
1810035L17RIK	0.00224615	-1.10825
HNRNPH1	0.049243	-1.10866
NAT5	0.0116483	-1.10875
ACPP	0.0207487	-1.10904
COL6A1	0.0661426	-1.10937
D10ERTD610E	0.0106417	-1.10964
DGAT2	0.0607384	-1.10973
LRP4	0.0516264	-1.10989
RNASEK	0.0527943	-1.11004
FLOT2	0.0550118	-1.11005
5930412G12RIK	0.0408876	-1.11051
BC039093	0.0508915	-1.11057
CD74	0.0681813	-1.11064
RGL2	0.0430807	-1.1109
EG632248	0.0848427	-1.11109
PARP14	0.0242203	-1.11121
S100A13	0.0170262	-1.11125
2210015K02RIK	0.00589538	-1.11131
OGFR	0.0528917	-1.11145
ETV4	0.00743857	-1.11197
RPP21	0.0566298	-1.11225
FBLN1	0.0330563	-1.11248
H2AFV	0.0168765	-1.11273
SEPW1	0.00106566	-1.1129
SAMD14	0.00135816	-1.11298
NDUFB3	0.0375529	-1.11309
A230057M07RIK	0.0181594	-1.11341
GPX8	0.00279338	-1.11352
HIST1H2AK	0.0777448	-1.1139
FCF1	0.00127015	-1.11401
ULK1	0.0230928	-1.11408
AP2S1	0.050924	-1.11471
PSMB9	0.0382117	-1.11492
FIS1	0.0219693	-1.11499
MOCS1	0.00179447	-1.1151
EPS8L2	0.0256031	-1.11572
D14ERTD668E	0.00860515	-1.11606
EG384525	0.0833196	-1.11624
LAMB2	0.0276035	-1.11665
CCDC56	0.0254346	-1.11742
DNAJC7	0.0662377	-1.11788

NDUFA9	0.0820708	-1.118
MGST3	0.0475331	-1.11801
LOC100039786	0.0521249	-1.11803
NPR2	0.0490031	-1.11808
0610031J06RIK	0.0110242	-1.11826
SUPT4H1	0.0754108	-1.11856
A230050P20RIK	0.0278594	-1.11869
TMED4	0.0118589	-1.119
2310016C16RIK	0.00112863	-1.11905
KTELC1	0.0754688	-1.11932
HBB-B1	0.0782909	-1.11934
LOC100048554	0.00613143	-1.11936
LOC280205	0.0518419	-1.11972
NIPSNAP1	0.00444099	-1.11977
9530064J02	0.0119146	-1.12009
SKAP2	0.00532268	-1.12035
IRF1	0.0496625	-1.12065
LOC100046802	0.0587386	-1.12088
EFHA1	0.0542349	-1.12128
GFPT2	0.00961725	-1.12238
LOC100047173	0.0530092	-1.12244
TMEM43	0.0187514	-1.1229
CUEDC2	0.0279883	-1.12327
2700084L06RIK	0.04373	-1.12353
MIB2	0.0399431	-1.12362
IRF9	0.00279905	-1.12391
IGTP	5.80E-05	-1.12412
UBE1L	0.0474797	-1.12488
GNB1	0.0429307	-1.1251
LOC381302	0.0195186	-1.12535
GLRX1	0.0161024	-1.12562
VCAM1	0.0510336	-1.12644
RAB25	0.0383405	-1.12696
RARRES2	0.0306901	-1.12698
NDUFC1	0.0291092	-1.12778
NDUFS4	0.0017403	-1.12835
ERGIC3	0.032527	-1.13
EPB4.1L4B	0.00791352	-1.13041
PTK7	0.0265933	-1.13084
MAPKAPK2	0.0164987	-1.13086
DLG5	0.0367576	-1.13094
LOC672474	0.00371065	-1.1314
EG667977	0.064345	-1.1315
CXCR4	0.0573391	-1.13152
FURIN	0.0623403	-1.13215
TRAFD1	0.0233355	-1.13221
SNRPG	0.0587917	-1.1326
TXNDC15	0.0699422	-1.13264
ABHD4	0.0136964	-1.13266
DTX3L	0.0549694	-1.13268
GLRX	0.00568145	-1.13335
GSTM2	0.00623464	-1.13351
IPO13	0.00394008	-1.13442
CAPZB	0.00648403	-1.13458
SLC4A2	0.0479205	-1.1351
MRPL28	0.0778511	-1.13562
COMMD3	0.0663292	-1.13572
MRPL23	0.00653314	-1.13597

ATP5G2	0.00124033	-1.13688
BCAS3	0.00289342	-1.13688
NPAL2	0.0193832	-1.13716
TMEM14C	0.0836365	-1.13797
TMEM205	0.0096554	-1.13813
CHIC2	0.0131036	-1.13843
LOC547343	0.00197303	-1.1386
NFIX	0.00253046	-1.13957
NDUFS8	0.0327416	-1.13964
IIGP2	0.0068677	-1.14126
TMEM208	0.0087328	-1.14152
LOC100044395	0.0659433	-1.14188
FAM20A	0.0316479	-1.14207
ITPKC	0.0626808	-1.14238
TRAPPC2L	0.0270889	-1.14269
SPPL3	0.0207286	-1.14321
LOC381398	0.0608975	-1.14334
MARCKSL1	0.0134686	-1.14364
INPP5K	0.00322714	-1.1446
2310016M24RIK	0.0460641	-1.14575
SDF2	0.0221997	-1.14605
PLEKHG2	0.0596689	-1.14627
B4GALNT1	0.0120861	-1.14661
SDC3	0.0736286	-1.14666
GSTT3	0.0226063	-1.14689
PPP2R1A	0.0758439	-1.14815
NENF	0.0158122	-1.14845
LAMP2	0.0234253	-1.14857
CALCA	0.080934	-1.14979
SBK	0.00313968	-1.15063
CUL7	0.0297652	-1.15072
GRCC10	0.0109145	-1.15142
PHLDA3	0.0301583	-1.15183
SCOTIN	0.0186678	-1.15257
PHPT1	0.0418963	-1.15263
LTBP3	0.0402988	-1.15293
LYZ	0.064394	-1.15296
RALBP1	0.0336346	-1.15417
ADAMTS4	0.0337392	-1.15426
RNPEP	0.0360706	-1.15435
5033414D02RIK	0.000932641	-1.15443
PER1	0.050395	-1.15451
5330430P07RIK	0.0806852	-1.15455
SAV1	0.0841323	-1.15491
NDUFC2	0.0401992	-1.15585
VKORC1	0.00901795	-1.1559
DPY30	0.00405034	-1.15606
LOC380707	0.0652185	-1.15641
GYLTL1B	0.0471688	-1.15723
LOC100045981	0.02386	-1.15809
D930038J03RIK	0.00200438	-1.15839
SH3YL1	0.0825183	-1.15839
GSTM1	0.0846829	-1.15906
RAB11B	0.01635	-1.15943
CASP2	0.0144866	-1.16139
SNX10	0.00468061	-1.1615
2310009A05RIK	0.0406381	-1.16204
SH3PXD2B	0.039909	-1.16263

MRPL53	0.00478526	-1.16278
TRIM32	0.019337	-1.16331
SUV420H2	0.0172395	-1.16435
LOC623453	0.0380216	-1.16487
LOC383330	0.0480022	-1.16516
CCDC85B	0.06984	-1.16745
JUNB	0.0235439	-1.16752
AKR7A5	0.00485796	-1.16769
ITFG3	0.00413126	-1.16821
RAB3D	0.0302288	-1.16879
ANTXR1	0.0434699	-1.16882
CISH	0.0638777	-1.16887
SH3BGRL3	0.0661514	-1.16972
PRCP	0.0270754	-1.17014
RIN2	0.0421701	-1.17064
AK3	0.0322994	-1.17065
5730437N04RIK	0.00878142	-1.17086
EG665509	0.0144518	-1.17111
FBLIM1	0.0440413	-1.17132
CENPB	0.0800408	-1.1724
SERINC2	0.0345528	-1.17301
LOC331102	0.0490741	-1.17306
ZFP532	0.0540821	-1.17397
TIAM1	0.0527706	-1.17478
TPRGL	0.0273188	-1.17526
PPIC	0.04003	-1.17575
NLGN2	0.00637749	-1.17604
LOC100046056	0.0334265	-1.17649
CYBA	0.00126781	-1.17689
LOC382061	0.0473301	-1.17714
PPP1CA	0.0475253	-1.17759
EBPL	0.0673097	-1.17783
HNRPA1	0.0174285	-1.17844
BCKDHA	0.00501361	-1.17845
MRPS33	0.0677484	-1.18057
EFEMP2	4.58E-05	-1.18156
FSTL1	0.00625107	-1.18158
CCL11	0.0113542	-1.18187
C330002I19RIK	0.0367537	-1.18224
STAC2	0.0834707	-1.18231
FBXO6	0.0254639	-1.18401
CCL2	0.0198386	-1.1861
LOC668387	0.065675	-1.18618
MRPL36	0.0742614	-1.18635
1190017O12RIK	0.0503512	-1.1871
VAMP8	0.0285137	-1.18821
OBRGRP	0.00774876	-1.19031
CLK1	0.0671439	-1.19154
PRLR	0.0608918	-1.19182
LOC675440	0.0393034	-1.19197
TOMM7	0.00105916	-1.19317
9130213B05RIK	0.0798018	-1.19338
EIF3K	0.0110951	-1.19342
LOC667370	0.0153214	-1.1938
IGSF3	0.0392683	-1.19427
LOC654426	0.061597	-1.19447
CLIP4	0.0263048	-1.1972
ARF6	0.0383362	-1.19924

WFDC12	0.0181231	-1.19982
SCARA3	0.0177186	-1.20011
LOC100048346	0.0140855	-1.20039
ARL6IP5	0.0139572	-1.20142
NDUFB8	0.0526584	-1.20224
ANAPC13	0.010397	-1.20267
FOXA1	0.0296852	-1.20443
PLCH2	0.0380547	-1.20537
NSMCE1	0.0411841	-1.20641
H3F3A	0.0369099	-1.20745
BSCL2	0.0431943	-1.20791
HINT2	0.0495333	-1.20888
0610006I08RIK	0.0289641	-1.2093
MCEE	0.013349	-1.20974
ZER1	0.0191015	-1.21022
1200009O22RIK	0.00827766	-1.21105
DDAH2	0.0407902	-1.21286
GSTM5	0.0450117	-1.21315
MMP14	0.00467515	-1.21408
ABHD14B	0.0531098	-1.21466
PTX3	0.014545	-1.21484
RPL22	0.00316799	-1.2151
SCL0002617.1_582	0.0367674	-1.21612
SEPX1	0.00728059	-1.21817
ATP5D	0.0609836	-1.2183
STXBP2	0.0062296	-1.21914
PSMB8	0.00881543	-1.21944
SHFM1	0.0153525	-1.21948
1110046J11RIK	0.0214144	-1.22223
PSMB10	0.00127331	-1.22267
ACOT7	0.0163658	-1.22432
FCGRT	0.00997364	-1.22506
USP18	0.0375372	-1.22623
TMEM9	0.00785682	-1.22625
CYB5	0.0311028	-1.22806
ACADM	0.0390717	-1.23001
ECHDC2	0.0402621	-1.23076
MRPL52	0.0151295	-1.23118
TIMM8B	0.0689283	-1.23189
CCDC120	0.058327	-1.23225
LOC100047353	0.0457047	-1.23542
AOF2	0.067505	-1.23598
HMGN3	0.00652154	-1.23746
LOC100048480	0.000268215	-1.2379
LOC433546	0.0444005	-1.23961
ITGB5	0.0217527	-1.24041
IFI47	0.0621723	-1.24208
SERPINF1	0.0447638	-1.24302
CDC2L6	0.0667843	-1.24345
HIST2H2AC	0.0829542	-1.24446
KNSL5	0.0684942	-1.24716
GPSN2	0.0178312	-1.24805
PRKD2	0.0336404	-1.24815
ATP5O	0.0116971	-1.24938
NDUFA7	0.0360303	-1.25189
RNF130	0.0670934	-1.25578
CTDSP2	0.00588642	-1.25604
CYB561	0.0559283	-1.25826

ATP5J	0.0143117	-1.25939
LOC100043796	0.0625544	-1.26125
LASP1	0.0473766	-1.26321
B230343A10RIK	0.0249139	-1.26372
INPPL1	0.0276659	-1.26374
LOC100044190	0.00188983	-1.26548
DDR1	0.00834908	-1.26558
FLOT1	0.0540598	-1.26597
H2-K1	0.00746332	-1.26723
IL11RA1	0.006229	-1.26793
GBP3	0.043107	-1.26895
C4A	0.0636906	-1.26949
2210013O21RIK	0.0781221	-1.27017
TSTA3	0.021261	-1.27091
TEAD2	0.00776337	-1.27371
MYLC2B	0.0512171	-1.27429
SMARCD2	0.0335042	-1.27431
0610007C21RIK	0.0455853	-1.27567
TIMP2	0.0472278	-1.2762
FAM173A	0.00964752	-1.27664
MIDN	0.000893845	-1.28549
COL4A5	0.0748639	-1.28572
MRPS24	0.018548	-1.28646
S100A1	0.0323741	-1.28665
EIF2AK2	0.00324041	-1.28737
LOC381629	0.0337509	-1.29087
MRPL33	0.0428219	-1.29154
2810410A03RIK	0.0278632	-1.29943
RSAD2	0.0331913	-1.29986
EG626367	0.00228626	-1.30767
LGALS9	0.0612261	-1.32206
PCBP2	0.0293559	-1.32225
LOC100047261	0.0235023	-1.33035
NDUFA6	0.0220081	-1.33091
1810027O10RIK	0.00897693	-1.33209
HIST1H2AI	0.0834462	-1.33244
NDUFA3	0.0449241	-1.33291
RABAC1	0.0680262	-1.33299
LGMN	0.00747109	-1.33334
PON2	0.00797611	-1.33368
TAGLN2	0.0442164	-1.33815
COL7A1	0.029011	-1.3395
PTN	0.0537753	-1.33954
OXCT1	0.0472923	-1.34007
EG434858	0.0101027	-1.34024
0910001L09RIK	0.00116721	-1.34168
CAR13	0.0190889	-1.34746
NDFIP1	0.0233183	-1.34759
SUMO2	0.0241531	-1.35198
CMC1	0.0409094	-1.35272
LOC225134	0.0351167	-1.35382
MTDNA_ND2	0.0243839	-1.35928
PLAU	0.0589671	-1.36366
LOC654467	0.0160456	-1.3649
TAP2	0.0576183	-1.365
TSC22D1	0.0260176	-1.36563
NGFRAP1	0.0673414	-1.36846
PRICKLE3	0.0162682	-1.36862

OASL2	0.0461749	-1.37466
SPINT1	0.0660841	-1.37568
SERPING1	0.0353255	-1.37837
LOC668038	0.0483887	-1.38041
DCAKD	0.0109582	-1.38065
LOC225058	0.0188108	-1.38294
GM2A	0.0345292	-1.38327
PSENEN	0.0387218	-1.384
GPX4	0.00483176	-1.38416
ST5	0.0325158	-1.39379
LOC100038882	0.0776974	-1.39642
AXUD1	0.0645014	-1.40396
DCN	0.0342003	-1.40499
PRDX2	0.0182122	-1.40545
AQP5	0.00636805	-1.40842
PFN1	0.0439231	-1.41145
TAX1BP3	0.00169694	-1.41448
RRAS	0.0472675	-1.41476
MARCKS	0.0176637	-1.41551
ECH1	0.0276857	-1.4164
FAM115C	0.0483145	-1.41672
SAMD9L	0.0152371	-1.42435
UROD	0.0347168	-1.425
CCL7	0.0147045	-1.4348
ATP6V0E	0.0121068	-1.44387
TSPAN14	0.0576748	-1.4447
EG637748	0.0636178	-1.44605
TCEB2	0.0837233	-1.44688
MIA1	0.00601607	-1.4469
COX6C	0.0482849	-1.45012
EG433229	0.00693763	-1.45073
CALM3	0.0728138	-1.45106
RPS5	0.0311413	-1.45794
LOC219145	0.00905749	-1.46519
H2-Q5	0.0201235	-1.4705
COL4A2	0.0456583	-1.47077
CAPNS1	0.0388305	-1.47333
LOC234987	0.0071998	-1.48031
1110012O05RIK	0.0280001	-1.48047
PHLDA1	0.0498439	-1.48175
TRIM29	0.0572838	-1.48262
RPL7A	0.0340699	-1.48431
FGG	0.00232677	-1.48734
GSTK1	0.0534019	-1.4958
PSME1	0.00114146	-1.49673
MTCH1	0.0306631	-1.49798
HN1	0.0215761	-1.50468
SSPN	0.0505455	-1.50861
LOC433749	0.000396743	-1.50877
LOC100039346	0.0552927	-1.51054
SOCS3	0.083512	-1.51462
MTDNA_ND5	0.00488188	-1.52769
RNPEPL1	0.0406538	-1.53433
EG666609	0.00355329	-1.53884
SPARC	0.0533067	-1.54976
LGALS3BP	0.0207588	-1.55198
LOC100048508	0.00615294	-1.55201
MYL9	0.0828757	-1.55522

100041294	0.0189152	-1.56368
PIGX	0.0399191	-1.57031
LOC383712	0.0586596	-1.57465
EG667728	0.0499133	-1.57535
LDB1	0.0170134	-1.58321
ATOX1	0.014638	-1.58382
KRTCAP3	0.00484767	-1.59428
1110020P15RIK	0.0715319	-1.60151
EG237361	0.0129798	-1.60914
LOC665250	0.0601179	-1.6205
TIMP1	0.0246485	-1.62551
NDUFA13	0.0166957	-1.62743
PSAP	0.072197	-1.6279
APOC1	0.0330317	-1.62894
S100A6	0.0812432	-1.63097
RNASE4	0.0841745	-1.63501
LOC386067	0.0567363	-1.65199
ZFP36	0.0382521	-1.65226
DYNLL1	0.0229352	-1.66433
TSPO	0.0037096	-1.68162
LOC624662	0.0155131	-1.68561
IFIT3	0.0198449	-1.6904
CASP1	0.0829533	-1.69887
GRN	0.000353254	-1.70265
MYEOV2	0.00770659	-1.70375
ATP5J2	0.057218	-1.72347
COL9A2	0.0262752	-1.72387
RPS13	0.0108973	-1.73005
0610010I05RIK	0.00995472	-1.73384
BAG3	0.0337712	-1.73756
PLEKHB1	0.0845155	-1.73871
RNF213	0.0305429	-1.75166
LOC100045312	0.00129705	-1.76173
GAS6	0.0624218	-1.77857
EG620119	0.0322686	-1.78283
RPL27A	0.0300022	-1.7907
MMP3	0.0265405	-1.80737
EG269105	0.0602691	-1.81525
EG668850	0.0337526	-1.85327
LOC100044475	0.0128254	-1.86092
LOC100048613	0.0460621	-1.87333
COX6B1	0.0658161	-1.87602
D12ERTD647E	0.0299185	-1.88051
RPL10A	0.0654611	-1.90091
ANGPTL4	0.0648228	-1.91847
CLEC2D	0.0379417	-1.93778
LOC100045005	0.00196382	-1.94889
CST3	0.00130249	-1.95805
LOC237512	0.0591774	-1.96464
LOC433476	0.00470297	-1.98832
HIGD2A	0.0607301	-1.99145
LOC674135	0.000364794	-2.01049
CTSB	0.0290082	-2.04768
LOC381283	0.0415287	-2.0546
ITM2B	0.0327632	-2.05836
2010107E04RIK	0.00546093	-2.08265
VPS28	0.0725108	-2.18414
RPS19	0.0397315	-2.21839

PDLIM4	0.0144563	-2.22723
RPL18	0.0270854	-2.22809
APOE	0.066748	-2.24312
SPON2	0.0132116	-2.24932
EG630499	0.0151032	-2.26741
PFDN5	0.0103387	-2.32661
LOC635470	0.0455297	-2.33451
C3	0.023809	-2.33602
LGALS7	0.0259242	-2.36141
CRIP2	0.0316501	-2.36397
H2-T23	0.0122109	-2.36504
SCD1	0.038903	-2.41067
RPL31	0.0428006	-2.42236
LOC665032	0.0103116	-2.46543
H2-D1	0.00415143	-2.47236
ITGB4	0.0261876	-2.4749
IFITM1	0.0842733	-2.55243
LOC238836	0.0300136	-2.55943
LOC241621	0.00565573	-2.62223
TBCA	0.0660328	-2.65695
ATPIF1	0.0499801	-2.66791
GPX1	0.00374564	-2.69731
RPL24	0.000415249	-2.7076
PDLIM3	0.0347059	-2.71978
COX4I1	0.0265473	-2.74614
LOC270037	0.0299004	-2.7748
IFI27	0.0227096	-2.83048
LOC433745	0.00331762	-2.84877
LOC100048187	0.0129402	-2.92384
RPS10	0.00177301	-2.93887
MMP2	0.0388902	-3.04815
MGP	0.0130788	-3.14252
LOC100043192	0.025485	-3.16167
LOC546015	0.0488696	-3.29373
LOC100048037	0.0256761	-3.34347
RPS15	0.0135807	-3.42544
RBP1	0.0156459	-3.43482
MYLK	0.0734257	-3.49748
LOC668573	0.0443162	-3.50959
LOC100041864	0.067768	-3.55743
LOC100047615	0.00607289	-3.58906
EG666577	0.0409299	-3.67377
EG268795	0.0044941	-3.84391
EG432721	0.00280287	-3.84829
LOC100043209	0.0182627	-4.3151
RPL26	0.0209261	-4.31568
LOC272681	0.00487239	-4.34074
TMEM176B	0.0517817	-4.41236
RPS9	0.0067595	-4.41978
UQCRH	0.0130412	-4.42582
LY6E	0.0257215	-4.76094
LOC382885	0.01698	-5.70528
B2M	0.0149109	-5.92886
LOC623568	0.0841065	-6.22119
RPL18A	0.0470338	-6.25387
LOC381999	0.0619129	-6.49551
FXYD3	0.0545115	-7.10046
RPL35	0.0331646	-7.41324

RPL27	0.0273655	-8.09888
COL16A1	0.0359733	-8.26647
AGRN	0.0682321	-9.2063
S100A11	0.0114578	-9.48566
LY6A	0.0240223	-9.77615
LOC100047998	0.0187009	-9.97774
EG626175	0.028917	-10.0387
TRF	0.0616034	-12.3116
RPS7	0.0555259	-13.662
EG432681	0.0590724	-14.7484
RPS21	0.00932409	-16.7402
MYL6	0.0268189	-17.2723
IFITM3	0.0157297	-39.5663

Supplementary Table 2. List of genotyping primers.		
**Long range PCR primers**	**Primer sequence (5' – 3')**	**Size (bp)**
Bcl11a-5'-Fwd	GAGACAAGAACAGGTGCAAGAGTGGATT	6,435
Bcl11a-5'-Rev	CAAGGAAACCCTGGACTACTGCGCCCTA	
Bcl11a-3'-Fwd	GAAAGAACCAGCTGGGGCTCGACTAGAG	3,446
Bcl11a-3'-Rev	CAGCGAGGTCCCCTTTCTCACTAAAAAT	
**Genotyping PCR primers**	**Primer sequence (5' – 3')**	**Size (bp)**
Bcl11a-lacZ-Fwd	GCTTGCTTTGGAATATGAATGTTTG	
Bcl11a-lacZ-Rev1	CATATATGGGGTTTATGGAGTAACC	363 (Wt); 437 (Mut)
Bcl11a-lacZ-Rev2	CAAGGAAACCCTGGACTACTGCGCCCTA	
Bcl11a-cko-Fwd	TAGCTCCTGCTAGCCAGGTTTCTT	377 (Wt);
Bcl11a-cko-Rev	CGAGGCTTGCAGAAACAGAAAGAT	470 (Cko)
Bcl11a-cko-Del	CTCGAAGGGAGGTTTCGGTATTGTG	700 (Del)
Rosa26-ERT-Cre-Fwd	TGTGGACAGAGGAGCCATAAC	
Rosa26-ERT-Cre-Rev1	CATCACTCGTTGCATCGACC	500 (Wt); 300 (Mut)
Rosa26-ERT-Cre-Rev2	AAGACCCAACCAACAGCAG	
p53 exon10 Del- Rev	GAAGACAGAAAAGGGGAGGG	600 when deleted
p53 exon1 cko-Fwd	CACAAAAACAGGTTAAACCCAG	300 (Wt); 400 (cko)
p53 exon1 cko-Rev	AGCACATAGGAGGCAGAGAC	

Supplementary Table2. List of primers used for qRT-PCR analysis.	
**RT-PCR primers**	**Primer Sequence (5'-3')**
Bcl11a-Fwd	TGGTATCCCTTCAGGACTAGGT
Bcl11a-Rev	TCCAAGTGATGTCTCGGTGGT
Cyclophilin A-Fwd	CCTTGGGCCGCGTCTCCTT
Cyclophilin A-Rev	CACCCTGGCACATGAATGGTG
